# TXA11114: Discovery of an *In Vivo* Efficacious Efflux Pump Inhibitor (EPI) That Potentiates Levofloxacin Against *Pseudomonas aeruginosa*

**DOI:** 10.3390/antibiotics15040346

**Published:** 2026-03-27

**Authors:** Jesus D. Rosado-Lugo, Pratik Datta, Ahmad Altiti, Yongzheng Zhang, Jun Lu, Yi Yuan, Ajit K. Parhi

**Affiliations:** TAXIS Pharmaceuticals, Inc., Monmouth Junction, NJ 08852, USA; jrosado@taxispharma.com (J.D.R.-L.); pdatta@taxispharma.com (P.D.); aaltiti@taxispharma.com (A.A.); yzhang@taxispharma.com (Y.Z.); jlu@taxispharma.com (J.L.); yiyuan@taxispharma.com (Y.Y.)

**Keywords:** *P. aeruginosa*, RND efflux pumps, antimicrobial drug resistance, efflux pump inhibitor, indole carboxamide, *in vivo* efficacy

## Abstract

**Objectives:** Multidrug-resistant (MDR) *Pseudomonas aeruginosa* represents a major clinical challenge, driven in part by resistance–nodulation–division (RND) efflux pumps that reduce intracellular antibiotic concentrations and limit the efficacy of many antibacterial agents, including fluoroquinolones. The aim of this study was to identify and characterize TXA11114 as a small-molecule efflux pump inhibitor (EPI) capable of restoring the activity of the fluoroquinolone levofloxacin against MDR *P. aeruginosa*. **Methods:** The antibacterial activity of the TXA11114–levofloxacin combination was evaluated using minimum inhibitory concentration (MIC) assays against panels of clinical isolates. Mechanistic studies included levofloxacin accumulation assays, ethidium bromide accumulation assays, outer-membrane permeability measurements, and whole-genome sequencing of mutants with altered potentiation phenotypes. *In vivo* efficacy was evaluated in murine thigh and lung infection models, while preliminary safety and drug-like properties were assessed using cytotoxicity assays and *in vitro* ADME profiling. **Results:** The TXA11114–levofloxacin combination produced > 1 log_10_ CFU reductions in bacterial burden in murine thigh and lung infection models, exceeding the activity of levofloxacin monotherapy. TXA11114 markedly potentiated levofloxacin activity, producing substantial reductions in levofloxacin MIC values across multiple MDR clinical isolates, and also enhanced the activity of several additional efflux pump substrates, including β-lactams, tetracyclines, chloramphenicol, and trimethoprim–sulfamethoxazole. Mechanistic experiments demonstrated increased intracellular accumulation of efflux substrates without evidence of nonspecific membrane disruption, and mutations in *ompH* were associated with altered potentiation phenotypes. **Conclusions:** The TXA11114–levofloxacin combination produced significantly greater bacterial reductions than levofloxacin monotherapy in murine infection models. Levofloxacin was selected because fluoroquinolone resistance in *P. aeruginosa* is frequently driven by efflux-mediated mechanisms. While this study focused on levofloxacin potentiation, future work will evaluate additional efflux pump substrates and further define the molecular target of TXA11114.

## 1. Introduction

The Centers for Disease Control and Prevention (CDC) and the World Health Organization (WHO) recognize antimicrobial resistance (AMR) as one of the most serious threats to modern healthcare systems. According to the CDC 2019 report, AMR was responsible for approximately 1.27 million deaths globally and was associated with nearly 5 million deaths overall. Within the United States, an estimated 2.8 million AMR infections occur annually, leading to roughly 35,000 deaths [1]. More recent CDC surveillance in 2024 indicates that resistance rates increased further during the post-COVID-19 period compared with pre-pandemic levels [2]. Gram-negative pathogens are particularly difficult to treat because of their impermeable outer membrane and the frequent coexistence of multiple resistance determinants, both of which contribute to multidrug resistance and clinical treatment failure [3]. Portions of this background section build upon our prior work [4].

*Pseudomonas aeruginosa* is an opportunistic Gram-negative pathogen notable for its metabolic adaptability and intrinsic resistance mechanisms, and it is widely regarded as a paradigm organism for antimicrobial resistance. The organism is a major cause of hospital-acquired and chronic infections and remains associated with substantial morbidity and mortality worldwide [5].

In 2017, the incidence of *P. aeruginosa* infection within the Military Health System (MHS) was 30.6 cases per 100,000 persons per year, whereas drug-resistant infections (defined as resistance to ≥3 antibiotic classes) occurred at a rate of 1.6 per 100,000 individuals. Nearly half (47.9%) of infections were healthcare-associated. Importantly, no tested antibiotic achieved 100% susceptibility among *P. aeruginosa* isolates in the MHS dataset. Among drug-resistant infections identified in 2017, 5.8% were classified as multidrug-resistant (MDR), 0.1% as extensively drug-resistant (XDR), and 1.3% as possibly XDR (PXDR). Fluoroquinolones were the most frequently prescribed class (55.1%), followed by penicillins with inhibitors (16.6%) and cephalosporins (10.7%). Within the fluoroquinolone class, ciprofloxacin (33.5%) and levofloxacin (21.6%) together accounted for more than half of prescriptions associated with *P. aeruginosa* infections in the MHS, and both agents exhibited susceptibility below 90% [6].

Resistance in Gram-negative organisms arises through several complementary mechanisms, including reduced drug uptake, target modification, enzymatic inactivation, and active efflux [3,7]. Multidrug resistance in *P. aeruginosa* frequently results from the combined contribution of these mechanisms, among which the overproduction of multidrug efflux pumps plays a central role in both intrinsic and acquired resistance by actively exporting structurally diverse antibiotics from the bacterial cell [7,8,9,10,11]. Many clinically important efflux systems in Gram-negative bacteria belong to the resistance–nodulation–division (RND) family of tripartite transporters [12,13]. *P. aeruginosa* encodes numerous RND pumps that collectively contribute to its multidrug-resistant phenotype [9]. Four extensively studied systems (MexAB-OprM, MexCD-OprJ, MexEF-OprN, and MexXY-OprM) are commonly observed in clinical isolates. Increased expression of *mexB*, *mexF*, and *mexY* has been reported in approximately 27%, 12%, and 45% of evaluated clinical isolates, respectively [11]. Other resistance mechanisms also contribute to antimicrobial resistance in *P. aeruginosa*, including porin loss (e.g., OprD deficiency), which has been reported in up to ~70–80% of carbapenem-resistant isolates [14,15], as well as β-lactamase overexpression or acquisition, which occurs in approximately ~20–35% of clinical isolates depending on geographic region and clinical setting [16,17]. Additional RND transporters (MexJK, MexGHI-OpmD, MexVW, MexPQ-OpmE, MexMN, and TriABC-OpmH) may further contribute to resistance in clinical settings [18,19,20,21].

Efflux-mediated resistance can be addressed through either direct or adjunctive strategies. One approach involves developing new antibacterial agents that evade recognition by efflux systems. Alternatively, efflux pump inhibitors (EPIs) can be paired with existing antibiotics whose activity has been compromised by active extrusion. Given the limited pace of novel antibiotic discovery, pharmacological inhibition of efflux has emerged as an attractive strategy to restore the effectiveness of legacy antimicrobials [21,22]. An effective EPI is expected to block transporter function sufficiently to increase intracellular antibiotic exposure and thereby resensitize resistant bacteria [23]. The identification of EPIs with demonstrated *in vivo* activity against *P. aeruginosa* could enable clinically viable combination therapies for multidrug-resistant infections, an objective that has remained elusive. Lowering the minimal inhibitory concentration (MIC) of established antipseudomonal agents such as levofloxacin below clinical breakpoints in resistant isolates could support renewed clinical utility [24]. In susceptible populations, EPI co-administration may further suppress MIC values and reduce the emergence of resistant subpopulations. Thus, efflux inhibition represents a complementary strategy to traditional antibiotic discovery by leveraging existing drugs, potentially reducing, though not eliminating, the time and resources required relative to de novo antibiotic development.

**Figure 1:**
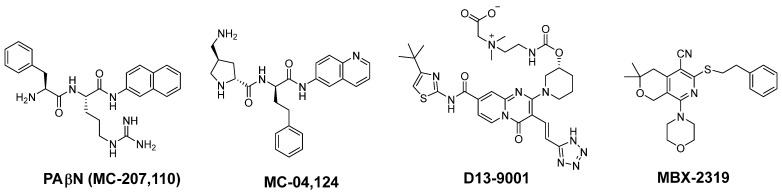
Previously known representative efflux pump inhibitors [4]. Reprinted with permission from Yuan, Y.; Rosado-Lugo, J.D.; Zhang, Y.; Datta, P.; Sun, Y.; Cao, Y.; Banerjee, A.; Parhi, A.K. Evaluation of Heterocyclic Carboxamides as Potential Efflux Pump Inhibitors in *Pseudomonas aeruginosa*. *Antibiotics*, **2021**, *11(1)*, 30. Copyright © 2021 MDPI.

Multiple chemical scaffolds have been reported to possess EPI activity (Figure 1) [25,26,27,28]. Early examples include the peptidomimetic MC-207,110 (phenylalanine-arginine β-naphthylamide, PAβN) and the related dipeptide amide MC-04,124, both originally developed by Essential Therapeutics Inc., which potentiate levofloxacin in wild-type and efflux-overexpressing *P. aeruginosa* strains [18,20]. Among non-peptide-based inhibitors, D13-9001 represents a notable pyridopyrimidine-based series designed for selective inhibition of the MexAB-OprM pump [21]. More recently, MBX-2319, a pyranopyridine derivative, has been shown to enhance the activity of ciprofloxacin, levofloxacin, and piperacillin in *Escherichia coli* [22]. However, the progress of these EPIs toward clinical use has largely stalled because of toxicity liabilities, discontinuation of development programs, or limited activity against *P. aeruginosa.* For example, PAβN and related peptidomimetic EPIs have been reported to exhibit suboptimal pharmacokinetic properties and tissue accumulation associated with renal toxicity, which contributed to their lack of clinical advancement [23,29], and MC-04,124 development ceased following the closure of Essential Therapeutics Inc. [26]. The MBX series lacks measurable efflux inhibition in *P. aeruginosa* [27], and although D13-9001 demonstrated promising preclinical efficacy, it has not yet advanced to clinical evaluation [25,28]. Accordingly, there remains a critical need for new, development-ready EPIs active against Gram-negative pathogens, particularly *P. aeruginosa.*

**Figure 2:**
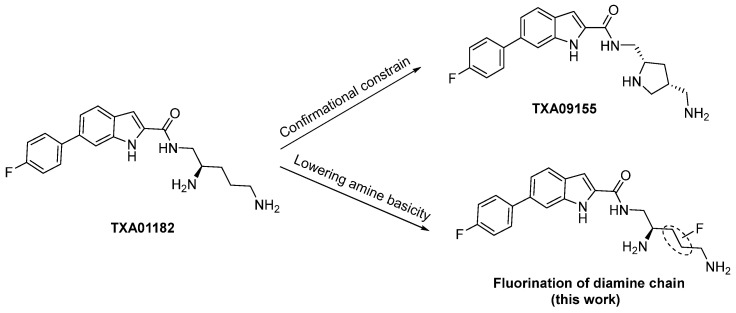
Sequential progression of TAXIS’s carboxamide EPIs.

Building on this unmet need, TAXIS Pharmaceuticals Inc. is advancing EPIs as potential first-in-class adjuvant therapies to counter efflux-mediated multidrug resistance [30]. Our earlier studies described a diaminopentanamide series and, more recently, heterocyclic carboxamide potentiators that led to the identification of TXA01182 [4] and its conformationally constrained analog TXA09155 (Figure 2) [31]. Both compounds enhanced the activity of monobactams, fluoroquinolones, sulfonamides, and tetracyclines against *P. aeruginosa* at concentrations as low as 6.25 μg/mL. In addition, TXA01182 and TXA09155 demonstrated synergy with levofloxacin against multiple MDR *P. aeruginosa* clinical isolates, with TXA09155 exhibiting greater potency.

Despite encouraging *in vitro* performance, neither TXA01182 nor TXA09155 improved levofloxacin efficacy in murine thigh or lung infection models. These findings suggested that suboptimal systemic exposure and/or intracellular sequestration may limit *in vivo* activity. Consistent with this hypothesis, both compounds exhibited relatively low maximum tolerated doses (MTDs) in mice (12.5 mg/kg and 30 mg/kg, respectively), indicating that toxicity-related exposure limits may also contribute to the lack of *in vivo* efficacy.

To address these liabilities, we introduced one or two β-fluorine substituents within the diamine linker to modulate basicity through inductive effects. This design was intended to reduce cationic amphiphilicity and lysosomal trapping, modestly increase lipophilicity to support passive diffusion, bias side-chain conformation via the β-fluoroamine gauche effect to potentially enhance pump engagement, and attenuate a predicted metabolic soft spot. Such minimal, mechanism-guided matched-pair modifications are commonly employed to translate potent *in vitro* EPIs into compounds with improved pharmacokinetic behavior and *in vivo* performance [32,33,34].

Both lead chemotypes require two amine functionalities for activity; removal of one or both amines abolishes potentiation. While basic centers often improve aqueous solubility and target engagement, highly basic or lipophilic amines can introduce developability risks, including hERG channel interaction, CYP binding, and reduced membrane permeability due to excessive ionization [35,36,37,38,39,40,41]. Moreover, compounds with cationic amphiphilic character are prone to lysosomal accumulation, particularly in tissues enriched in lysosome-dense cells [42]. Intralysosomal trapping in renal tissue has been widely associated with nephrotoxicity [43] and this liability is thought to have contributed to the discontinuation of the PAβN series during preclinical development [4,29]. These precedents highlight the need to carefully tune amine basicity in EPI pharmacophores that depend on protonatable groups for activity.

**Figure 3:**
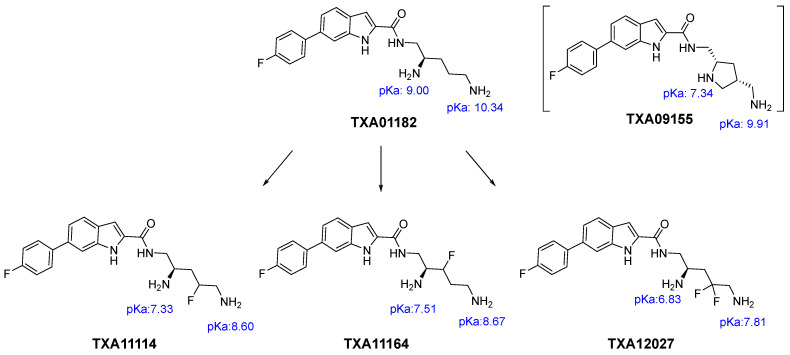
Structures of five TAXIS EPIs with or without fluorine on the side chains.

Although strong electron-withdrawing substituents (e.g., nitriles, esters, or heteroatom-rich heterocycles) can be used to reduce amine pKa, such modifications often introduce additional pharmacophoric elements that may perturb binding mode, permeability, or off-target interactions. In contrast, hydrogen-to-fluorine substitution offers a subtler electronic adjustment while largely preserving steric footprint and overall scaffold geometry. Accordingly, fluorine incorporation was explored in the TXA11114 diamine series as a strategy to fine-tune amine basicity and associated physicochemical properties without introducing new functional groups that could confound structure–activity relationships or efflux-related mechanisms (Figure 3). Importantly, fluorine substitution can influence molecular behavior through multiple mechanisms including inductive effects, conformational bias (e.g., the β-fluoroamine gauche effect), polarity changes, and local dipole modulation depending on substitution pattern and position. Consistent with this complexity, the comparative behavior of TXA11114 and TXA11164 suggests that fluorine placement within the diamine linker contributes to activity through effects that extend beyond simple pKa modulation. The present design therefore leveraged fluorination as a minimally perturbing matched-pair strategy while empirically interrogating position-dependent structure–property relationships within the series.

Fluorine is widely used in medicinal chemistry to modulate pKa, enhance intrinsic potency, and improve metabolic and pharmacokinetic properties [34,44,45]. Within our diamine side chain, the basicity of both amines can be systematically tuned through strategic placement of fluorine atoms between them. Mono- and difluoro substitution at two distinct carbon positions was therefore expected to differentially influence amine pKa depending on substitution pattern and position. The present study evaluates whether selective fluorination of the diamine linker can reduce basicity while preserving EPI activity and improving tolerability.

## 2. Results and Discussion

### 2.1. Synthesis of Fluorinated EPI Analogs

Fluorination of TXA01182 generated three analogs (Figure 3). Mono-fluorination at C-3 or C-4 and di-fluorination at C-4 afforded TXA11114, TXA11164, and TXA12027, respectively, providing a focused set to interrogate position-dependent structure–property relationships within this series. Calculated amine pKa values (ACD/LC) indicated that di-fluorination at C-4 (TXA12027) reduced basicity by more than two units relative to TXA01182, whereas mono-fluorination at C-3 (TXA11114) or C-4 (TXA11164) produced reductions of approximately 1.5 units. These matched analogs therefore enabled assessment of how fluorine placement influences both basicity and functional EPI performance.

**Scheme 1:**
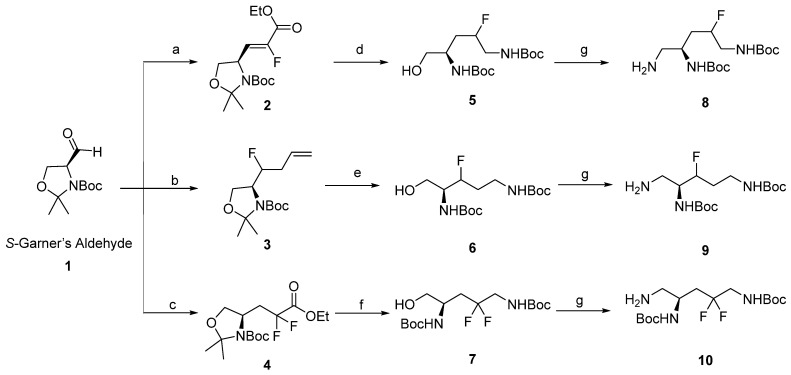
Synthesis of fluorinated triamines. Reagents and reaction conditions: (**a**) Reference [46]: triethyl 2-fluoro-2-phosphoacetate LiHMDS, THF, −40 °C-rt, 16 h, (78%). (**b**) References [47,48]: 1. Allyl bromide, activated Zn dust, THF, 50 °C, 6 h (65%); 2. DAST, DCM, −78 °C-rt, 16 h (64%). (**c**) References [50,51]: 1. BrCF_2_CO_2_Et, Zn, ultrasounds, THF, rt, 8 h.; 2. TCDI, DCE, rt, 20 h; 3. Et_3_SiH, Bz_2_O_2_, reflux 1.5 h. (**d**) 1. H_2_ gas (1 atm), Pt/C, MeOH; 2. LiBH_4_, MeOH; 3. phthalimide, Ph_3_P, DIAD, THF, 0 °C-rt (2 h); 4. NH_2_NH_2_.H_2_O, EtOH, 90 °C (2 h); 5. 4 M HCl/dioxane, MeOH, 50 °C, 2 h; 6. Boc_2_O, Et_3_N, DCM, 4 h (39% over 4 steps). (**e**) 1. K_2_OsO_4_, NaIO_4_, THF-H_2_O, 16 h, 0 °C-rt; 2. NaBH_4_, MeOH, rt (20 min); 3. phthalimide, Ph_3_P, DIAD, THF, 0 °C-rt (2 h); 4. NH_2_NH_2_^.^H_2_O, EtOH, 90 °C (2 h); 5. 4 M HCl (in dioxane), MeOH, 50 °C, 2 h; 6. Boc_2_O, Et_3_N, DCM, 4 h (68% over 7 steps). (**f**) 1. NaBH_4_, THF-MeOH (5:1), 0 °C-rt (2 h) (80%); 2. Tf_2_O, pyridine, DCM, 0 °C-rt (45 min), then phthalimide, NaH, DMF, 125 °C, 24 h (53%, over two steps); 3. NH_2_NH_2_.H_2_O, EtOH, 90 °C (2 h); 4. 4 M HCl (dioxane), MeOH, 50 °C, 2 h; 5. Boc_2_O, Et_3_N, DCM, 4 h (27% over 5 steps). (**g**) 1. phthalimide, Ph_3_P, DIAD, THF, 0 °C—rt (2 h); 2. NH_2_NH_2_^.^H_2_O, EtOH, 90 °C (2 h).

**Figure 4:**
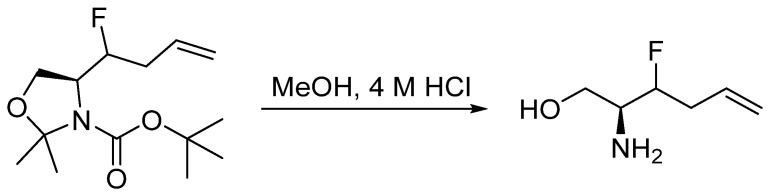
Detection of the homoallylamines diastereomeric ratio using ^1^HNMR.

To evaluate the fluorine-induced modulation on the amine pKa, binding potential, and the antibacterial activity in the proposed EPIs, we invested in synthetic protocols that allow selective vicinal fluorination of each amine moiety. Therefore, we selected the accessible intermediates **2**, **3**, and **4** (Scheme 1), all made from commercially available S-Garner aldehyde **1**. We applied the Horner–Wadsworth–Emmons reaction on aldehyde **1** according to the literature [46] using triethyl 2-fluoro-2-phosphoacetate and LiHMDS afforded alkene **2** as a 3:1 E/Z mixture in 78% yield. Hydrogenation of the double bond in **2** using Pt/C followed by a sequence of LiBH_4_ reduction of the ester moiety, Mitsunobu amination of the resulting alcohol, and finally, protecting group modifications afforded alcohol **5** in 39% over four steps. The allylation of Garner’s aldehyde **1** using allyl bromide and activated Zn dust at 50 °C affords the corresponding *erythro* and *threo* homoallyl alcohols as a 1:1 mixture [47,48], which was converted to homoallyl fluoride mixture **3** at −78 °C by nucleophilic fluorination using DAST in 64% [49]. Cleavage of the acid-labile-protecting groups in a small sample of mixture **3** revealed that the diastereomeric ratio of the fluorinated derivatives is 4:1 based on the ^1^HNMR analysis (See Supporting Information in Supplementary Materials; Figure 4). Oxidative cleavage of the double bond in **3** with NaIO_4_/K_2_OsO_4_.H_2_O gave the corresponding aldehyde, which was reduced using NaBH_4_. The resulting alcohol was then used to introduce the distal amine moiety as phthalimide using Mitsunobu amination. Functional group modifications furnished the fluorinated protected diamine alcohol **6** in 68% yield with fluorine atom installed vicinal to the proximal amine group at C3. A subsequent Mitsunobu amination followed by hydrazine cleavage of the corresponding phthalimide furnished the triamine derivatives **8** and **9** in 75% and 53% yield over two steps, respectively. The difluoro ester derivative **4** [50,51] was reduced by NaBH_4_ in the THF/MeOH to give the corresponding alcohol in 80% yield; functionalizing the resulting alcohol using the Mitsunobu reaction was unfruitful. Alternatively, the alcohol was converted to the corresponding triflate, which was subsequently subjected to Gabriel amine synthesis using sodium phthalimide at 125 °C, which afforded the desired compound in a 53% yield over two steps. Functional group transformations on the resulting phthalimide provided alcohol **7** in 27% yield over five steps. The same amination protocol used in the previous two examples was applied to intermediate **7** to accomplish the amine **10** in 80% with difluoride at C4, vicinal to the distal amine moiety.

**Scheme 2:**
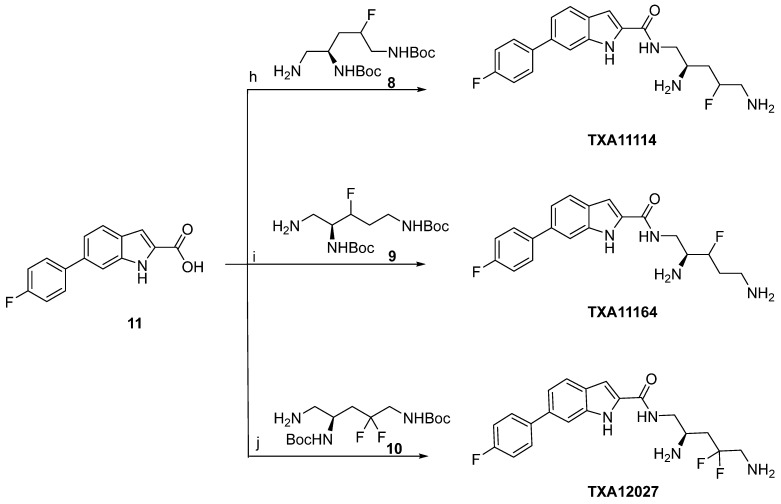
Synthesis of EPIs containing fluorinated side chains. Reagents and conditions: (**h**) 1. Amine **8**, EDC, HOBt, DMF. 2. 4 M HCl (in dioxane) (78%). (**i**) 1. Amine **9**, HATU, DMF, Et_3_N, 20 min., 2. 95% TFA, 2.5% Et_3_SiH, 2.5% H_2_O. 64%, over two steps). (**j**) 1. Amine **10**, HATU, DMF, Et_3_N, 20 min. 2. 95% TFA, 2.5% Et_3_SiH, 2.5% H_2_O (85%, over two steps).

Having the desired fluorinated amine intermediates in hand, we turned our attention to finishing the synthesis of target EPIs (Scheme 2). Accordingly, amines **9** and **10** were used to synthesize TXA11164 and TXA12027 in 64% and 85%, respectively, via HATU-mediated coupling with acid **11** [4] followed by Boc deprotection using TFA. TXA11114 was synthesized by coupling amine **8** to acid **11** in 78% using EDC/HOBt followed by Boc deprotection using 4 M HCl solution (Scheme 2).

### 2.2. Evaluation of the Fluorinated EPIs for Their Potentiation of Levofloxacin

Once synthesized, the three fluorinated EPI analogs (TXA11114, TXA11164 and TXA12027) were first assayed for their antibacterial activities against *P. aeruginosa* ATCC 27853. They had MICs of 100, >200 and 100 µg/mL, respectively (Table 1). The potentiation abilities of these three fluorinated compounds with levofloxacin were then determined at 6.25 μg/mL and compared with two first generation EPIs: TXA01182 and TXA09155 [4,31]. Among the three EPIs, only TXA11114 could potentiate levofloxacin by 8-fold, comparable to TXA01182 and TXA09155 (Table 1). Strikingly, TXA11164 and TXA12027 failed to potentiate levofloxacin. Particularly, the inactivity of TXA11164 was surprising as the pKa values of both the amines are similar to that in TXA11114. Based on our previous findings, EPIs that are inactive in combination with levofloxacin have often remained inactive in other combinations also, prompting the exclusion of TXA11164 and TXA12027 from further analysis [4,31].

### 2.3. TXA11114 Potentiates Antibiotics with Efflux Liabilities

Given the observed potentiation of levofloxacin, TXA11114 was further evaluated in combination with additional antibiotics (Table 2). At sub-inhibitory concentrations ranging from 25 to 3.13 µg/mL (1/4 to 1/32 MIC), TXA11114 reduced the MICs of multiple agents in *P. aeruginosa* in a concentration-dependent manner. The strongest potentiation was observed with tetracyclines and the fluoroquinolone levofloxacin. Levofloxacin and minocycline MICs decreased 8- and 16-fold, respectively, while doxycycline and cotrimoxazole each exhibited 8-fold potentiation. Chloramphenicol also showed notable potentiation (>4-fold reduction). β-lactam antibiotics were moderately affected, with MIC reductions primarily in the 2- to 4-fold range. In contrast, the activities of gentamicin and imipenem were unchanged in the presence of TXA11114 under the conditions tested. Although gentamicin can be transported by the MexXY efflux system, the absence of potentiation in this assay is not sufficient to draw conclusions regarding MexXY inhibition or overall efflux specificity [52].

Compared with structural analogs TXA09155 and TXA01182, TXA11114 demonstrated broadly comparable potentiation across several antibiotic classes, although the magnitude of enhancement was compound-dependent. The lowest effective concentration of TXA11114 was 6.25 µg/mL (1/16 MIC), a sub-inhibitory level lacking intrinsic antibacterial activity, and this concentration was therefore selected for subsequent potentiation assays in *P. aeruginosa*.

### 2.4. TXA11114 Demonstrates Broad Levofloxacin Potentiation Across MDR P. aeruginosa Clinical Isolates

In a comparative study, the levofloxacin-potentiating activity of TXA11114 was evaluated alongside the well-characterized EPIs PAβN and MC-04,124 using *P. aeruginosa* clinical isolates obtained from the CDC and FDA Antimicrobial Resistance Isolate Bank and the Walter Reed Army Institute of Research (WRAIR) [18,20]. At 6.25 µg/mL, TXA11114 reduced the levofloxacin MIC by ≥8-fold in approximately 85% of CDC isolates. Under the conditions tested, PAβN (50 µg/mL) potentiated ~54% of isolates, whereas MC-04,124 produced minimal MIC shifts (Table 3). While PAβN has been widely studied in laboratory strains, its activity against the CDC/FDA *P. aeruginosa* panel has been reported only sparingly, limiting direct cross-study comparisons.

TXA11114 exhibited *in vitro* activity against MDR clinical isolates that was broadly comparable to that of its non-fluorinated analog TXA01182 [4] and its constrained analog TXA09155 [31]. When evaluated against 20 highly MDR isolates from the Multidrug-resistant Organism Repository and Surveillance Network (MRSN) at WRAIR [53], 65% of isolates were shifted to levofloxacin susceptibility in the presence of TXA11114, whereas levofloxacin alone showed MIC values ≥ 8 μg/mL. Across this panel, TXA11114 produced 2- to 32-fold MIC reductions (Table 4).

Genotypic analysis identified *nalC*-G71E and *mexR*-V126Q substitutions in several isolates. These mutations have previously been reported to confer derepression of the MexAB-OprM efflux pump and are commonly associated with increased efflux activity in *P. aeruginosa* [54,55,56,57,58]. In the present study, efflux pump expression was not directly measured; rather, the presence of these mutations was interpreted based on their established association with MexAB-OprM overproduction in the literature. In contrast, strains harboring stacked QRDR mutations (e.g., *gyrA*-T83I with *parC/parE* variants) generally exhibited more modest potentiation [59,60,61]. Notably, TXA11114 also enhanced levofloxacin activity in some isolates lacking canonical efflux-associated mutations, suggesting that additional resistance determinants or regulatory effects may contribute to the observed phenotype.

Overall, TXA11114 potentiated levofloxacin in >90% of the combined CDC/FDA and WRAIR MDR isolate sets and restored susceptibility to levofloxacin at or below the EUCAST breakpoint (≤2 μg/mL) in approximately 60% of strains. While the magnitude of potentiation varied by resistance genotype, these data support the continued evaluation of TXA11114 as an efflux-targeting adjuvant for fluoroquinolone-resistant *P. aeruginosa.*

### 2.5. TXA11114 Does Not Affect Outer- and Inner-Membrane Integrity in P. aeruginosa

**Figure 5:**
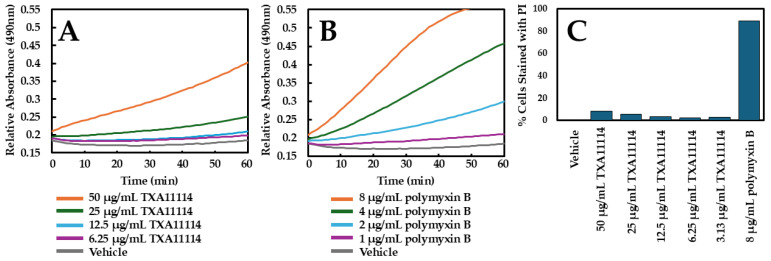
*P. aeruginosa* ATCC 27853 outer- and inner-membrane permeabilization studies with TXA11114. TXA11114 dose response effect on bacterial outer- and inner-membrane was determined. Polymyxin B was used as positive control. NCF hydrolysis (**A**,**B**) was used as read out for outer-membrane activity while inner-membrane activity was determined by measurement of PI fluorescence (**C**) using flow cytometry. Experiments were repeated three times to ensure reproducibility.

The significant toxicity of previously identified EPIs has prevented their clinical use. So, understanding the TXA11114 mechanism of action (MoA) was of paramount importance to eliminate off-target effects that may arise from other activities besides efflux pump inhibition. Historically EPIs are known to augment the action of traditional antibiotics by compromising the integrity of bacterial membranes, allowing increased drug accumulation. One of the major concerns of earlier EPIs was their ability to disrupt bacterial membranes. Two approaches were taken to investigate if membrane disruption played a role in the potentiation of antibiotics seen in Table 2 by TXA11114: (1) a flow cytometry-based propidium iodide (PI) assay to monitor inner-membrane permeabilization, and (2) a nitrocefin (NCF) assay to monitor outer-membrane permeabilization. NCF is a chromogenic cephalosporin that changes from yellow to red when the amide bond in the β-lactam ring is hydrolyzed by a β-lactamase. The rate of hydrolysis in intact cells is slow, as it is limited by the rate of diffusion of periplasmic β-lactamase across the outer membrane. However, in the presence of an agent that permeabilizes the outer membrane, the rate of hydrolysis will increase. The impact of TXA11114 on NCF hydrolysis is minimal at concentrations below 25 μg/mL, suggesting that TXA11114 does not interact with the outer membrane of *P. aeruginosa* at these concentrations (Figure 5A). This result is comparable to what was previously reported for TXA01182 and TXA09115 [4,31], suggesting that the incorporation of the fluorinated side chain of TXA11114 did not introduce membrane disruption properties to the compound. Polymyxin B was used as a positive control (Figure 5B) to establish a benchmark for robust outer-membrane permeabilization, whereas PAβN (while reported to exhibit modest outer-membrane effects) was not included, as the objective of this assay was to detect gross membrane disruption rather than subtle permeability changes associated with efflux inhibition. In the PI assay, log-phase *P. aeruginosa* cells were mixed with various concentrations of TXA11114 for 1 h at 37 °C while shaking followed by the addition of PI. Cells with intact membranes exclude PI and remain non-fluorescent, while cells with compromised membrane integrity allow PI to enter and bind to DNA, resulting in fluorescence [62]. TXA11114 did not disrupt the bacterial inner membrane below concentrations of 50 μg/mL compared with water and polymyxin B as vehicle and positive controls, respectively (Figure 5C).

### 2.6. TXA11114 Blocks Efflux of Ethidium Bromide and Levofloxacin

**Figure 6:**
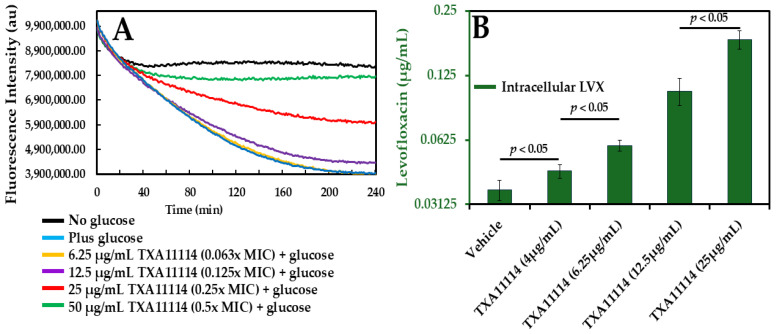
*P. aeruginosa* efflux inhibition by TXA11114. TXA11114 concentration-dependent blockade of EtBr efflux (**A**) and accumulation of levofloxacin in live cells (**B**). Experiments were done in triplicates. Data are presented either as a representative value (**A**) or as a mean value with standard deviations (**B**).

The ability of TXA11114 to inhibit bacterial efflux was studied using two quantitative assays. In the first assay, the efflux of ethidium bromide (EtBr) by *P. aeruginosa* cells is studied in the presence of different concentrations of TXA11114. Type strain *P. aeruginosa* ATCC 27853 cells were incubated with EtBr to allow for intracellular accumulation and treated with carbonyl cyanide 3-chlorophenylhydrazone (CCCP) to inhibit active efflux. When bound to intracellular bacterial DNA, EtBr fluoresces brightly, while any unbound EtBr outside bacterial cells exhibits little or no fluorescence. Following removal of extracellular CCCP, the addition of glucose reactivates EtBr efflux, which can be monitored in real time as a concentration-dependent decrease in fluorescence in the presence of TXA11114. As seen in Figure 6A, the fluorescence intensity increased proportionally with the increasing concentration of TXA11114, indicating intracellular accumulation of EtBr and supporting a role in efflux inhibition by TXA11114. Notably, TXA11114 shows reduced apparent activity in the EtBr accumulation assay when tested at 6.25 µg/mL. While this assay is well-suited to demonstrate intracellular EtBr accumulation in the presence of compounds that inhibit efflux, it requires a substantially higher bacterial inoculum (approximately 1000-fold greater than that used in the broth microdilution MIC assay). This elevated inoculum shifts the minimal potentiation concentration (MPC) of TXA11114 from 6.25 to 12.5 µg/mL, thereby explaining the lack of EtBr accumulation at the lower concentration. In contrast, levofloxacin potentiation is evaluated under standard broth microdilution MIC conditions using a substantially lower bacterial inoculum. Supplemental Table S5 illustrates this inoculum-dependent MPC shift for TXA11114 using a bacterial density 2.5-fold higher than that recommended for broth microdilution MIC testing in combination with levofloxacin.

The second assay measures levofloxacin accumulation inside *P. aeruginosa* following treatment with varying concentrations of TXA11114 [31], providing more direct evidence of its effectiveness as an EPI in live bacterial cells. *P. aeruginosa* DA7232 harboring mutations in DNA gyrase (*gyrA*-T83I) and topoisomerase IV (*parC*-S80L) was used for this study since it is highly resistant to levofloxacin [63]. Moreover, the levofloxacin MIC in this strain goes from 256 µg/mL to 1 µg/mL in the presence of 6.25 µg/mL TXA11114, making this strain ideal for the study. In this assay, *P. aeruginosa* DA7232 cells were incubated with levofloxacin in the presence or absence of TXA11114 to permit intracellular accumulation. After washing and membrane permeabilization, intracellular levofloxacin accumulation was monitored by following the changes in levofloxacin fluorescence. TXA11114-treated cells showed a marked increase in intracellular levofloxacin accumulation compared with vehicle-treated controls (Figure 6B). This assay, previously described for similar applications [64,65], demonstrated that TXA11114 promotes levofloxacin accumulation in a concentration-dependent manner, consistent with its role as an efflux inhibitor.

### 2.7. TXA11114 Shows No Effect on the Inner-Membrane Potential and Cellular ATP Content

**Figure 7:**
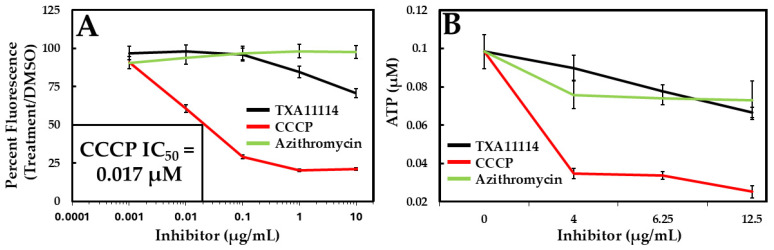
Bacterial membrane potential was assessed by measuring fluorescence changes in the membrane potential indicator DiOC_2_(3) (**A**). Cellular ATP pool was measured by luminescence-based assay (**B**). Experiments are done in triplicates; data are presented as mean value with standard deviations.

RND efflux pumps require an intact proton motive force across the inner membrane to mediate drug efflux [63]. Accordingly, collapse of the membrane potential could indirectly disable efflux activity and result in apparent antibiotic potentiation. To determine whether TXA11114 dissipates the membrane potential of *P. aeruginosa*, membrane polarization was assessed using the fluorescent probe 3,3′-diethyloxacarbocyanine iodide (DiOC_2_(3)) [66]. DiOC_2_(3) accumulates in cells with polarized membranes, undergoing a concentration-dependent shift from green to red fluorescence due to dye stacking; membrane depolarization prevents this shift [67]. As shown in Figure 7A, TXA11114 did not induce significant membrane depolarization over the concentration range tested (0.01–20 µM; 20 µM = 7.32 µg/mL), indicating that collapse of the membrane potential is unlikely to be responsible for antibiotic potentiation. CCCP and azithromycin were included as positive and negative controls, respectively. A modest increase in apparent depolarization was observed at TXA11114 concentrations above ~10 µg/mL; however, this effect was not accompanied by membrane disruption as assessed by the propidium iodide (PI) assay, which showed no permeability changes below 50 µg/mL (Figure 5C). This discrepancy likely reflects a methodological limitation of the DiOC_2_(3) assay in Gram-negative bacteria. Specifically, EDTA treatment is required to transiently permeabilize the outer membrane to permit DiOC_2_(3) access to the cytoplasmic membrane (see Methods). While necessary for probe uptake, EDTA can sensitize cells to secondary perturbations and may exaggerate subtle fluorescence changes at supraphysiological compound concentrations. Importantly, no depolarization was observed at concentrations relevant to antibiotic potentiation, supporting the conclusion that TXA11114 does not act through membrane depolarization under physiologically relevant conditions. 

Further, a deviation from normal membrane function can affect the activity of respiratory components and diminish ATP synthesis. Thus, an indirect approach to assess a drug’s membrane effect is by monitoring cellular ATP levels after treatment. To exclude ATP depletion as a TXA11114 MoA, bacterial ATP levels were evaluated three hours post-treatment. As shown in Figure 7B, *P. aeruginosa* treatment with TXA11114 did not result in ATP depletion. In contrast, a significant depletion of ATP was observed in CCCP-treated cells, compared to the untreated control. Azithromycin was used as a negative control.

### 2.8. TXA11114 Potentiates Levofloxacin in Efflux Pump Overexpressed Strains

Next, the ability of TXA11114 to inhibit *P. aeruginosa* mutants overexpressing MexAB-OprM was evaluated. Loss of function mutations in *nalB* resulting in the overexpression of MexAB-OprM have been identified in *P. aeruginosa* clinical isolates and are associated with resistance to cephalosporins, fluoroquinolones and aminoglycosides [68,69,70,71,72,73,74]. In theory, an EPI should reverse the antimicrobial susceptibility lost in bacteria-overexpressing efflux pumps. Thus, the TXA11114 EPI activity was studied using antibiotics that display efflux liabilities in this RND efflux pump (Table 5). The level of antibiotic potentiation seen with TXA11114 in the MexAB-OprM-overproducing strain (K1455) was 2- to 4-fold higher than the parent strain (K767). In agreement with being an EPI, TXA11114 did not significantly potentiate any antibiotic in the in MexAB-OprM-deficient strain (K1119). Finally, imipenem, which is not the substrate of RND efflux pumps in *P. aeruginosa*, was not potentiated by TXA11114 in any of the strains tested. Notably, TXA11114 potentiation of levofloxacin activity in efflux overexpression mutants were comparable to non-fluorinated EPI TXA01182 and constrained EPI TXA09155 [4,31].

### 2.9. TXA11114 Prolonged the Levofloxacin Post-Antibiotic Effect

Antimicrobials exhibit a post-antibiotic effect (PAE) after their removal and introduce a growth delay on bacterial cultures when compared to the untreated condition. An exponentially grown *P. aeruginosa* ATCC 27853 was treated with 1× MIC of levofloxacin in the presence and absence of 6.25 µg/mL of TXA11114 for 1 h. After treatment, drug concentrations were reduced by diluting cultures 50-fold, and bacterial growth was monitored by measuring optical density. When compared to untreated control, bacterial cultures treated with levofloxacin alone displayed a PAE of 4.9 h, whereas the TXA11114–levofloxacin combination-treated bacteria displayed a PAE of 6.3 h. Thus, the combination prolonged the levofloxacin PAE by 1.4 h. The prolonged PAE of the combination is consistent with EPI-driven levofloxacin accumulation within the cell and could aid in determining the optimum dosing frequency of the antibiotic by ensuring properly spaced dosing intervals.

### 2.10. TXA11114–Levofloxacin Combination Has Undetectable Level of Resistance in Drug-Sensitive and Drug-Resistant Strains

In addition to reducing the levels of intrinsic resistance, a potent EPI is also expected to significantly reverse acquired resistance as well as decrease the frequency at which antimicrobial resistance evolves. A resistance study for the TXA11114–levofloxacin combination was investigated in drug-sensitive (ATCC 27853) and drug-resistant (AR-0232, AR-0248 and AR-0249) *P. aeruginosa* strains. For this study, levofloxacin was tested at 4× its MIC (determined in the absence of EPI), while TXA11114 was evaluated at a fixed concentration of 6.25 µg/mL. We found that the combination resulted in undetectable levels of spontaneous levofloxacin resistance against 10^10^ CFU/mL bacterial inputs. On the contrary, standalone levofloxacin resulted in significant levels of resistance from the same bacterial input (10^10^ CFU/mL, Table 6). The undetectable levels of resistance highlight the clinical utility of these combinations to limit the selection of spontaneous resistant mutants, particularly in cystic fibrosis patients infected with *P. aeruginosa*, where patients are colonized by hypermutable strains that persist for years [75]. In future frequency-of-resistance studies, the levofloxacin concentration in the combination arm will be normalized to the EPI-shifted MIC to ensure comparisons are performed at equivalent multiples of the effective MIC.

### 2.11. Genetic Study of TXA11114 Resistance in P. aeruginosa

To gain insight into potential cellular targets involved in TXA11114 activity and its role in levofloxacin potentiation, *P. aeruginosa* ATCC 27853 was exposed to increasing concentrations of TXA11114, either alone or in combination with levofloxacin (Table 7). When exposed to TXA11114 alone, no resistant colonies emerged at 4× MIC (400 µg/mL). At 1× MIC (100 µg/mL), resistant colonies appeared at a frequency of 1.8 × 10^−6^. Whole-genome sequencing of three representative mutants (EPIR32, EPIR36, EPIR38) revealed independent nonsense mutations in *ompH*, which encodes a homolog of the outer-membrane chaperone Skp [76]. Resistance to TXA11114 did not confer cross-resistance to other antibiotics (Supplemental Table S1). In fact, the *ompH* mutants displayed increased susceptibility to multiple antibiotics, including levofloxacin, ceftazidime, tigecycline, doxycycline, meropenem, and amikacin, consistent with the role of Skp/OmpH in maintaining outer-membrane protein homeostasis [77,78]. Notably, the increased susceptibility to levofloxacin and doxycycline mirrored the potentiation observed in the wild-type strain treated with sub-inhibitory TXA11114 concentrations (Table 2). To directly test whether OmpH contributes to the synergistic effect of TXA11114 with levofloxacin, MIC assays were performed using the *ompH* mutants. Unlike the parent strain, these mutants no longer exhibited TXA11114-mediated potentiation of levofloxacin (Supplemental Table S2), suggesting that OmpH function is required for the observed synergy. In *E. coli*, the periplasmic chaperone Skp interacts with more than 30 envelope proteins and exhibits genetic interactions with *acrD*, a component of the AcrAD-TolC efflux system [79,80,81,82]. OmpH is a homologous periplasmic chaperone that binds newly synthesized, unfolded outer membrane proteins (OMPs) and delivers them to the outer membrane for assembly by the BAM machinery, thereby preventing aggregation and facilitating proper membrane insertion [83,84,85]. Given the substantial sequence similarity (62%) between *E. coli* AcrD and *P. aeruginosa* MexB, it is plausible that OmpH plays a conserved role in the transport of efflux pump components prior to their functional assembly in the outer membrane. Under this model, OmpH function would precede and enable effective efflux activity, providing a mechanistic basis for the observed OmpH dependence of TXA11114–levofloxacin synergy. Alternatively, TXA11114 may directly perturb OmpH function itself, indirectly impairing efflux capacity by disrupting efflux pump delivery, assembly or stability. While accumulation and genetic studies support efflux modulation as a key contributor to TXA11114 activity, the precise molecular target remains unresolved. The requirement for OmpH is consistent with both direct, OmpH-dependent interference with efflux pump function and indirect inhibition through the disruption of OmpH-mediated outer-membrane transport or the folding of efflux components. Although further validation is required to distinguish between these possibilities, these findings collectively indicate that OmpH plays a supporting role in the mechanism underlying TXA11114–levofloxacin synergy and warrant further mechanistic investigation.

### 2.12. TXA11114 Promotes Bacterial Killing by Levofloxacin

**Figure 8:**
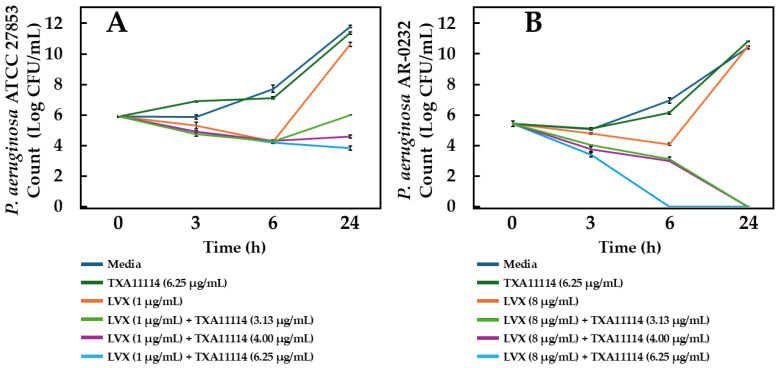
Time killing kinetics of levofloxacin alone or in combination with different doses of TXA11114 against *P. aeruginosa* type strain ATCC 27853 (**A**) and clinical isolate AR-0232 (**B**). Data are presented as mean values with standard deviations derived from technical triplicates.

In addition to assessing the potentiation activity of TXA11114 *in vitro,* its potentiation of a minimally bactericidal concentration of levofloxacin (1X-MIC) was probed against *P. aeruginosa* ATCC 27853 and AR-0232 with time-kill studies. Figure 8 shows time-kill curves with levofloxacin alone or combined with TXA11114. By itself, TXA11114 had no effect on the growth of *P. aeruginosa* strains at 6.25 µg/mL (dark green curve). TXA11114 enhanced levofloxacin killing kinetics in a concentration-dependent manner (light green, purple and light blue curves). Across both strains tested, TXA11114 enhanced levofloxacin killing kinetics relative to levofloxacin alone, with AR-0232 exhibiting ≥ 3-log_10_ reductions and near-complete eradication at higher TXA11114 concentrations by 24 h, whereas ATCC 27853 showed more modest but reproducible reductions. These results suggest that the killing kinetics for the TXA11114/levofloxacin combination are faster than those of levofloxacin alone, similar to what was reported previously for the TXA01182/levofloxacin and TXA09155/moxifloxacin combinations [4,31].

### 2.13. TXA11114 Has Good Physiochemical and ADME Properties

Along with its microbiological evaluation, TXA11114 was assessed for its physicochemical and *in vitro* ADME properties. In general, TXA11114 complies with Lipinski’s Rule of Five, suggesting favorable physicochemical characteristics for oral bioavailability, and it exhibits high aqueous solubility (>145 µM at pH 7.4). Table 8 summarizes the *in vitro* ADME-T (absorption, distribution, metabolism, excretion, and toxicity) properties of TXA11114, including plasma protein binding, stability in plasma and microsomes, solubility, lipophilicity, and CYP450 inhibition profiles. TXA11114 showed good plasma stability across four species (human, dog, rat, and mouse), with half-lives exceeding 2 h. Microsomal stability was also favorable in both human and rat liver microsomes, with half-lives exceeding 1 h, suggesting low susceptibility to rapid metabolic degradation. Plasma protein binding ranged from 98 to 99% across the four species tested. TXA11114 exhibited minimal inhibition of cytochrome P450 isoforms, with IC_50_ values greater than 100 µM for CYP1A2, CYP2C19, CYP2C9, and CYP2D6, and 63 µM for CYP3A4. For comparison, the CYP2D6 and CYP3A4 IC_50_ values for TXA09155 were lower than those for TXA11114, suggesting a higher potential for CYP-related liabilities for TXA09155 and an improved CYP safety profile for TXA11114. In CellTiterGlo™ cytotoxicity assays using HEK293T and A549 cells, no cytotoxicity was observed for TXA11114 at concentrations up to 20 µM, the highest concentration tested; therefore, IC_50_ values could only be reported as >20 µM. While testing at higher concentrations was not performed in these initial screens, future studies will be required to more precisely define the *in vitro* therapeutic index of TXA11114.

Overall, the *in vitro* ADME profile of TXA11114 indicates a favorable balance of physicochemical and metabolic properties consistent with further preclinical development. The compound combines high aqueous solubility at physiological pH (~146 µM) with moderate lipophilicity (cLogP 2.34), suggesting a favorable balance between solubility and membrane permeability. Its high plasma and microsomal stability supports the potential for adequate systemic exposure *in vivo*. Although TXA11114 shows high plasma protein binding (98–99%), this level of binding is common among lipophilic antibacterial agents and may contribute to prolonged circulation time. Importantly, the minimal inhibition of major CYP isoforms indicates a low risk of clinically relevant drug–drug interactions. Collectively, these findings support the suitability of TXA11114 as a lead compound for further antibacterial optimization.

### 2.14. TXA11114 Has a Low Cardiotoxicity Potential

Unwanted binding to voltage-gated ion channels in eukaryotic cells can lead to significant side effects due to their role in pharmacokinetics and pharmacodynamics [86]. To see how the strategic placement of fluorine in the TXA11114 diamine side chain affected the activities on the three ion channel targets (voltage-gated sodium: Nav1.5 (peak), voltage-gated potassium: hERG, and voltage-gated calcium: Cav1.2) was assayed using Eurofins’s QPatch electrophysiological platform (Table 9). As shown in Table 9, inhibition levels of Nav1.5 and Cav1.2 channels were lower in TXA11114 than TXA09155, suggesting that fluorine addition reduces unwanted binding to these channels. The inhibition of hERG by both EPIs was comparable. These results indicate that TXA11114 did not exhibit overt ion channel liabilities within the concentration range tested. However, because the upper bound of the cardiotoxicity assessment does not fully exceed the *in vitro* potentiation exposure window, these data should be considered preliminary and not yet sufficient to define a therapeutic safety margin. Additional concentration-ranging safety studies will be required to more fully characterize the cardiotoxicity risk profile of TXA11114.

### 2.15. TXA11114 Shows No Evidence of Acute Nephrotoxicity in an Initial Rat Screen

Because cationic molecules with lysosomotropic properties can accumulate in renal tissue, nephrotoxicity represents a potential liability for EPI chemotypes containing protonatable amines [43]. To obtain an initial assessment of renal safety, blood urea nitrogen (BUN) and serum creatinine (CRE) were measured following TXA11114 administration.

BUN and CRE analyses were conducted by SRI International (Menlo Park, CA, USA). Adult male Sprague Dawley rats (n = 3 per group) received TXA11114 (10 mg/kg, IV, QD), and blood samples were collected 24 h after dosing. As shown in Table 10, mean BUN and CRE values following TXA11114 administration (15.3 and 0.22 mg/dL, respectively) were comparable to historical normal male rat values (16 and 0.28 mg/dL) and showed no treatment-related elevations in this short-term assessment.

These findings indicate that TXA11114 did not produce detectable changes in standard clinical chemistry markers of renal injury under the conditions tested. This initial study was designed as an early hazard screen to identify overt renal safety signals. Future studies will expand this assessment through evaluation at multiple dose levels, larger group sizes, extended observation periods, and the incorporation of kidney histopathology and systemic exposure measurements. Pharmacokinetic characterization will also be integrated to enable exposure–toxicity relationship analyses.

The 10 mg/kg dose was selected as a conservative exploratory dose consistent with the compound’s intended low-micromolar adjuvant use. Additional repeat-dose and tissue exposure studies are planned to further define the renal safety profile of TXA11114.

### 2.16. TXA11114 and Levofloxacin Have Complementary Pharmacokinetic Profile

**Figure 9:**
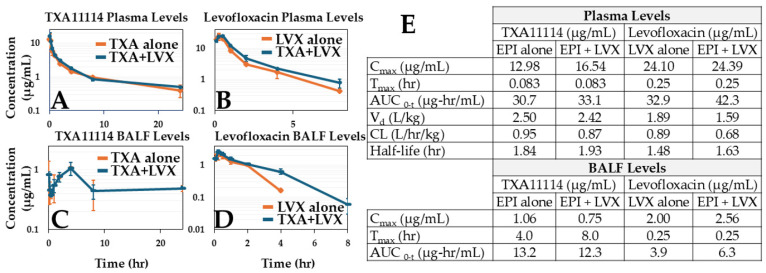
Pharmacokinetic studies of TXA11114. (**A**) Plasma concentration-time profiles of TXA11114 following intravenous (IV) administration (30 mg/kg) alone or in combination with levofloxacin. (**B**) Plasma concentration-time profiles of levofloxacin following subcutaneous (SC) administration alone or in combination with TXA11114. (**C**) Bronchoalveolar lavage fluid (BALF) concentration-time profiles of TXA11114 following IV administration alone or in combination with levofloxacin. (**D**) BALF concentration-time profiles of levofloxacin following SC administration alone or in combination with TXA11114. (**E**) Pharmacokinetic parameters and urea-based BALF dilution correction used for exposure analysis. TXA11114 was administered intravenously (IV) and levofloxacin subcutaneously (SC). Data represent mean concentrations ± SD from three mice per time point. BALF concentrations reflect recovered lavage fluid and were not corrected to epithelial lining fluid.

To maximize pharmacodynamic (PD) benefit, the pharmacokinetics (PK) of an efflux pump inhibitor (EPI) should be compatible with, but not necessarily identical to, those of its partner antibiotic. To assess whether TXA11114 exhibits PK properties compatible with levofloxacin, plasma and bronchoalveolar lavage fluid (BALF) concentrations were determined for each compound when administered alone or in combination in a *P. aeruginosa* lung infection mouse model (Figure 9A–D). In plasma, TXA11114 exposures were comparable when administered alone or with levofloxacin, indicating no evidence of a drug–drug interaction. At a 30 mg/kg dose, TXA11114 achieved peak plasma concentrations (C_max_) of 12.98–16.54 µg/mL at 0.083 h, with total plasma exposure (AUC) of 30.7–33.1 µg·h/mL, a volume of distribution of 2.42–2.50 L/kg, plasma clearance of 0.87–0.95 L/h/kg, and an elimination half-life of 1.84–1.93 h (Figure 9E). Similarly, plasma PK parameters for levofloxacin were comparable when administered alone or in combination with TXA11114, with C_max_ values of 24.1–24.39 µg/mL at 0.25 h and AUC values of 32.9–42.3 µg·h/mL. In contrast to plasma, BALF PK profiles differed markedly in both kinetics and magnitude. BALF concentrations represent analysis of recovered lavage fluid and were not corrected for epithelial lining fluid dilution (based on urea measurements; Figure 9E). TXA11114 exhibited delayed peak concentrations in BALF (C_max_ = 0.75–1.06 µg/mL at 4 h) and lower overall exposure (AUC = 12.3–13.4 µg·h/mL) relative to plasma, reflecting slower pulmonary distribution and prolonged retention. Levofloxacin BALF exposures were likewise lower and temporally distinct from plasma, with C_max_ values of 2.0 and 2.56 µg/mL and AUC values of 3.9 and 6.3 µg·h/mL when administered alone or in combination, respectively. BALF exposure of TXA11114 following a single 30 mg/kg dose appeared sub-therapeutic relative to *in vitro* activity (C_max_ = 1.06 µg/mL), suggesting that higher or repeated dosing may be required to achieve pharmacologically relevant lung concentrations. Importantly, co-administration did not adversely affect the plasma or pulmonary exposure of either compound. Together, these data demonstrate that TXA11114 and levofloxacin exhibit distinct but complementary PK profiles across plasma and lung compartments, and that combination dosing does not perturb the exposure of either agent, supporting further optimization of TXA11114 dosing strategies.

### 2.17. TXA11114 Has Improved Acute Toxicity Profile

With overall acceptable microbiological, physiochemical, ADME, toxicity and complementary pharmacokinetic characteristics, the next task was to evaluate TXA11114’s *in vivo* efficacy in a *P. aeruginosa* infection model. Thus, the safety and tolerability of TXA11114 was determined to find an optimum dosing capability. The maximum tolerated dose (MTD) of TXA11114 was assessed in female mice (CD-1, 6–8 weeks old) at the University of North Texas Health Science Center (UNTHSC). TXA11114 was administered IV to groups of three female mice. Mice were administered TXA11114 in the range 30–240 mg/kg (IV bolus). The first (30 mg/kg) dose level was administered and the mice were observed for any effects (including respiration, piloerection, startle response, skin color, injection site reactions, hunched posture, ataxia, salivation, lacrimation, diarrhea, convulsion, death and others if observed) for 10 and 90 min before proceeding to the next higher dose. As doses are tolerated (not resulting in effects such as mortality, convulsions, or other severe morbidity), they are increased. Survival and general observations as to the tolerability of the administered dose started immediately and continued for a period of 48 h after each dose was recorded. Following this dosing regimen, an MTD for TXA11114 was determined to be greater than 60 mg/kg and less than 90 mg/kg without causing any acute toxicity. For comparison, the non-fluorinated EPI TXA01182 has an MTD of 12.5 mg/kg while the constrained EPI TXA09155 has an MTD of 30 mg/kg. While *in vitro* potency remains comparable across these compounds, fluorine substitution in the diamine side chain resulted in a marked improvement in tolerability, increasing the MTD by more than 2- to approximately 5-fold relative to the non-fluorinated analogs.

### 2.18. TXA11114 and Levofloxacin Combination Is Efficacious in Murine Thigh and Lung Infection Models

**Figure 10:**
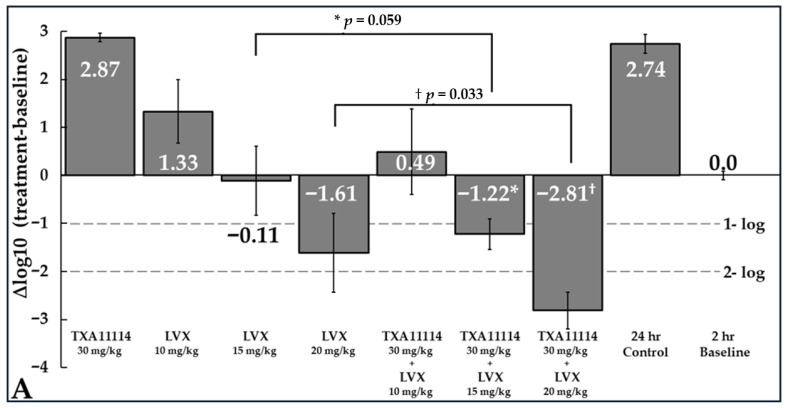

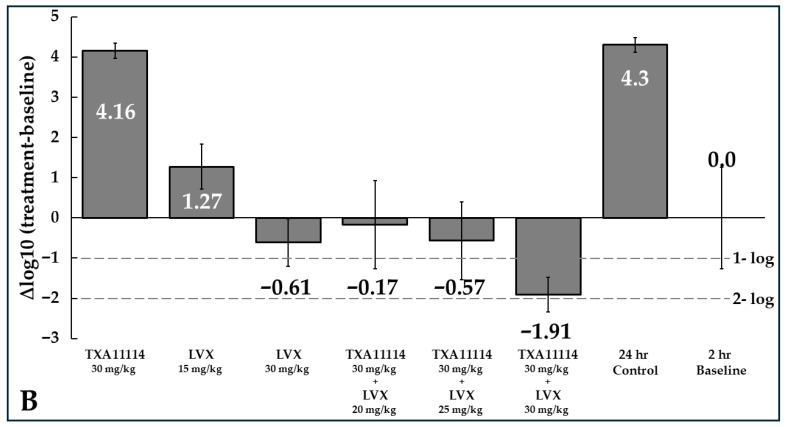
*In vivo* efficacy in murine thigh and lung infection models. (**A**) Efficacy of TXA11114/levofloxacin combination murine thigh infection model: In this model, mice were inoculated intramuscularly (IM) in the right thigh with 5.75 log_10_ CFU of *P. aeruginosa* ATCC 27853. Pretreatment control was determined 6.08 log_10_ CFU at 2 h (baseline). Then, 24 h after infection, the mean bacterial thigh titers reached 8.82 log_10_ CFU at 24 h (untreated control), confirming successful colonization. Mice were treated 2 h after infection with vehicle, TXA11114 (fixed 30 mg/kg, IV, Q6H), levofloxacin alone (10, 15, and 20 mg/kg, SC, Q6H) or in combination for 24 h. CFU/thigh was determined at 24 h after infection. Relative changes in CFU with treatment to baseline were plotted. Five mice were used per group. Tukey’s multiple comparisons test was used after an ANOVA to determine which specific pairs of group means were statistically different. * *p* < 0.05; † *p* < 0.01 (vs. control). (**B**) *In vivo* efficacy of TXA11114/levofloxacin in murine lung model. In the lung infection model, mice were inoculated intranasally with 4.83 log_10_ CFU of *P. aeruginosa* ATCC 27853. Pretreatment bacterial load in lung was 4.59 log_10_ CFU/lung determined 2 h. after infection (baseline). Bacterial load was increased to 8.89 log_10_ CFU at 24 h post-infection in the untreated control set. Mice for each group were treated with vehicle, TXA11114 (30 mg/kg, IV, Q6H), levofloxacin alone (30 mg/kg, SC, Q6H) or in combination for 24 h. Post-treatment, bacterial lung burden was determined 24 h after infection and changes in bacterial counts are represented relative to baseline. Data are represented as the mean ± standard deviation (SD), within an experimental group. A *p* value < 0.05 was considered statistically significant. Five mice were used per group. Tukey’s multiple comparisons test was used after an ANOVA to determine which specific pairs of group means were statistically different.

The translation of *in vitro* efflux pump inhibition into *in vivo* efficacy remains a key hurdle in EPI development. To evaluate whether TXA11114 can enhance levofloxacin activity *in vivo*, we assessed the TXA11114–levofloxacin combination in neutropenic murine thigh and lung infection models using *P. aeruginosa* ATCC 27853. Infection procedures and dosing regimens are described in the Methods section. Briefly, mice were infected intramuscularly (IM, thigh model) or intranasally (IN, lung model), and bacterial burdens in the infected tissues were quantified at 2 h post-infection (baseline) and 24 h post-infection in vehicle- and drug-treated groups. In the thigh infection model, TXA11114 alone (30 mg/kg, IV, QID) had no impact on bacterial burden, showing increases comparable to untreated controls at 24 h. Levofloxacin monotherapy at 10, 15, and 20 mg/kg (SC, QID) produced dose-dependent reductions in bacterial counts. When combined with TXA11114 (30 mg/kg, IV, QID), the antibacterial effect of levofloxacin was enhanced. At 20 mg/kg (SC, QID), levofloxacin alone reduced bacterial counts by 1.61 log_10_ CFU relative to baseline, whereas the TXA11114–levofloxacin combination achieved a 2.81 log_10_ CFU reduction (*p* = 0.033), corresponding to an additional 1.2 log_10_ CFU kill. A similar enhancement was observed at 15 mg/kg levofloxacin (SC, QID), though the difference narrowly missed statistical significance (*p* = 0.059, Figure 10A). In the lung infection model, TXA11114 alone (30 mg/kg, IV, QID) did not reduce bacterial burden. Levofloxacin monotherapy (30 mg/kg, SC, QID) reduced lung CFU by 0.61 log_10_; in contrast, co-administration with TXA11114 resulted in a 1.91 log_10_ CFU reduction at 24 h post-infection, representing an additional 1.3 log_10_ CFU kill compared with levofloxacin alone (Figure 10B).

Together, these results demonstrate that TXA11114 enhances the *in vivo* antibacterial activity of levofloxacin in both thigh and lung infection models, yielding > 1-log_10_ CFU greater reduction than levofloxacin alone at clinically relevant exposure levels.

MDR *P. aeruginosa* remains a major clinical challenge due largely to intrinsic and acquired resistance mechanisms such as RND-family efflux pumps, which reduce intracellular antibiotic concentrations and contribute to treatment failure for multiple drug classes, including fluoroquinolones. In this study, we describe the discovery and characterization of TXA11114, a fluorine-substituted indole carboxamide that potentiates the activity of levofloxacin against *P. aeruginosa*. TXA11114 restored fluoroquinolone susceptibility across diverse MDR clinical isolates, producing 4- to 32-fold reductions in levofloxacin MIC values in many strains. These results suggest that efflux-mediated resistance remains a dominant contributor to fluoroquinolone treatment failure and that pharmacological inhibition of efflux pathways may represent a viable strategy to restore antibiotic activity.

Mechanistic studies support an efflux-related mode of action for TXA11114. Ethidium bromide accumulation assays demonstrated increased intracellular dye retention in the presence of TXA11114, consistent with the inhibition of active efflux, while outer-membrane permeability assays did not indicate nonspecific membrane disruption. Whole-genome sequencing of mutants with altered potentiation phenotypes identified mutations in *ompH*, indicating that this gene is required for the observed synergy. Together, these findings suggest that TXA11114 enhances intracellular antibiotic accumulation through modulation of pathways associated with drug efflux.

Importantly, the potentiation observed *in vitro* translated into meaningful efficacy *in vivo*. In murine thigh and lung infection models, the TXA11114–levofloxacin combination produced substantially greater bacterial reductions than levofloxacin monotherapy. In addition, the combination improved bactericidal activity and suppressed the emergence of resistant subpopulations *in vitro*. These findings are particularly notable because many previously reported efflux pump inhibitors have failed to demonstrate meaningful activity in animal infection models due to limited potency, toxicity, or inadequate exposure.

Preliminary physicochemical and ADME profiling further suggests that TXA11114 possesses properties compatible with continued development. The compound exhibited favorable aqueous solubility, moderate lipophilicity, metabolic stability in plasma and microsomes, minimal inhibition of major CYP isoforms, and no detectable cytotoxicity in mammalian cell lines at the concentrations tested. Together, these characteristics indicate that TXA11114 avoids several liabilities that have historically hindered efflux pump inhibitor development.

Despite these encouraging findings, several limitations should be noted. Although the genetic and accumulation data support an efflux-related mechanism, the present study does not establish the direct molecular target of TXA11114. The requirement for *ompH* function suggests an important role for this protein in the potentiation phenotype, but it remains unclear whether TXA11114 directly engages OmpH or whether OmpH acts as a functional determinant within a broader efflux-related pathway. Future studies employing target engagement approaches such as photo-affinity labeling combined with proteomic enrichment or CETSA-based assays will help clarify the molecular interactions underlying TXA11114 activity.

In summary, TXA11114 represents a promising EPI that restores fluoroquinolone activity against MDR *P. aeruginosa*. By increasing intracellular antibiotic accumulation, enhancing bactericidal activity, and suppressing resistance emergence, TXA11114 provides a potential strategy to rejuvenate existing antibiotics against high-priority Gram-negative pathogens. Collectively, this study provides the first demonstration that the TXA indole carboxamide scaffold can function as an *in vivo*-active EPI capable of restoring fluoroquinolone efficacy against MDR *P. aeruginosa*. Further mechanistic studies and the optimization of pharmacokinetic properties will help define the therapeutic potential of this compound class as adjunctive antibacterial agents.

## 3. Materials and Methods

### 3.1. Bacterial Strains, Media, and Reagents

*P. aeruginosa* ATCC 27853 was obtained from the American Type Culture Collection (ATCC). *P. aeruginosa* multidrug-resistant isolates were obtained from the CDC and FDA Antibiotic Resistance Isolate Bank. *P. aeruginosa* DA7232 (*gyrA*-T83I, *parC*-S80L) was a kind gift from Prof. Dan I Andersson, Uppsala University, Uppsala, Sweden and has been characterized elsewhere [51]. *P. aeruginosa* strains K767 (WT), K1455 (*mexAB-oprM* overexpressed), K2415 (*mexXY-oprM* overexpressed) and K3698 (*oprM*∆) were obtained from Prof. Keith Poole, Queen’s University, Kingston, ON, Canada and have been characterized elsewhere [9,10]. Bacterial cells were grown in cation-adjusted Mueller Hinton (CAMH) media, brain heart infusion (BHI) broth or tryptic soy agar (TSA) plates all obtained from Becton, Dickinson, and Company (BD, Franklin Lakes, NJ, USA). Aztreonam, ceftazidime, moxifloxacin, levofloxacin, minocycline, tigecycline, chloramphenicol, nitrocefin and imipenem were purchased from TOKU-E (Bellingham, WA, USA). Azithromycin was purchased from Tokyo Chemical Industry (Portland, OR, USA). Cotrimoxazole was purchased from Toronto Research Chemicals (Toronto, ON, Canada). Doxycycline and polymyxin B were purchased from Sigma-Aldrich (St. Louis, MO, USA). Ethidium bromide (EtBr) and glucose were purchased from VWR (Radnor, PA, USA). MC-04,124 and TXA01182 were synthesized at TAXIS Pharmaceuticals. Carbonyl cyanide 3-chlorophenylhydrazone (CCCP) was purchased from Enzo Life Sciences (Farmingdale, NY, USA). DiOC_2_(3) and propidium iodide were obtained from Thermo Fisher Scientific (Waltham, MA, USA).

### 3.2. Determination of Minimum Inhibitory Concentration (MIC)

MIC assays for potentiation of antimicrobial activity against *P. aeruginosa* were performed as described previously [4,31].

### 3.3. Flow Cytometry Assay for Permeabilization of Inner Cell Membranes to Propidium Iodide (PI)

A flowcytometry assay used for assessing potential inner membranes permeabilization of *P. aeruginosa* bacterial cells to PI was conducted using the LIVE/DEAD BacLight Kit from Invitrogen (Waltham, MA, USA). Briefly, log-phase *P. aeruginosa* ATCC 27853 bacterial cells grown in BHI broth were diluted 5-fold in PBSM (1X PBS, 1xMgCl_2_) to an approximate concentration of 6.5 × 10^7^ CFU/mL. The bacteria were aliquoted into tubes and mixed with TXA11114 at concentrations ranging from 1 to 1/8th times the MIC (50 to 6.25 μg/mL). Water alone was used as a solvent control. Polymyxin B was used as a positive control. Intracellular PI fluorescence was detected by flow cytometry using a CytoFlex (Beckman Coulter Inc., Brea, CA, USA). The 488 nm laser was used for excitation, with the PC5.5 and FITC channels being used for emission. For each sample, the fluorescence of 10,000 individual bacterial cells was measured, and the percent of cells that stained positive for PI fluorescence was calculated.

### 3.4. Nitrocefin Cellular Assay for Outer Cell Membrane Permeabilization

Assessment of outer-membrane permeabilization in *P. aeruginosa* was carried out as described previously [4,31].

### 3.5. Assessment of Ethidium Bromide (EtBr) Efflux Inhibition

Efflux of EtBr from *P. aeruginosa* in the presence of TXA11114 was carried out as described previously [4,31].

### 3.6. Levofloxacin Accumulation Assay

*P. aeruginosa* DA7232 was grown to an OD_600_ of 0.6 and treated with levofloxacin (64 μg/mL = 1/4th MIC) alone or in combination with TXA11114 at sub-inhibitory concentrations (1/4th, 1/8th and 1/16th MIC). The bacterial culture samples were treated on ice for 15 min, centrifuged, washed with PBSMG (1X PBS, 1 mM MgCl_2_, 100 mM glucose) and then resuspended in 1 mL of glycine-HCl buffer (pH 3.0) overnight for cell lysis, followed by centrifugation. The fluorescence of 100 µL of supernatant was read at 490 nm following a 355 nm excitation in a SpectraMax iD5 spectrophotometer (Molecular Devices, San Jose, CA, USA). Fluorescence values were converted to levofloxacin concentrations using a standard curve generated from serial 10-fold dilutions of known levofloxacin concentrations.

### 3.7. Membrane Polarization Assay

*P. aeruginosa* membrane polarization assays were conducted as described previously [4,31] using strain ATCC 27853. Briefly, mid-exponential phase (OD_600_ of 0.5) *P. aeruginosa* was pelleted by centrifugation at 3000× *g* for 10 min at room temperature. Cells were then resuspended in 1× PBS, treated with 10 mM EDTA for 5 min and then centrifuged at 300× *g* for 10 min to remove EDTA. EDTA-treated cells were pelleted and resuspended to an OD_600_ of 1.0 in assay resuspension buffer [4,31]. A 6 mM DiOC_2_(3) stock in DMSO was added to cells for a final concentration of 30 µM. DiOC_2_(3)-loaded cells were then added to a 96-well black bottom microplate for a final volume of 200 µL. TXA11114 or control compounds were added to the bottom of the well of the microplate prior to the addition of the DiOC_2_(3)-loaded cells. After 15 min incubation at 37 °C in dark, DiOC_2_(3) fluorescence was recorded using the SpectraMax iD5 spectrophotometer (Molecular Devices, San Jose, CA, USA) using 450 nm excitation. Red fluorescence intensity was recorded at 670 nm emission.

### 3.8. Determination of Intracellular ATP Levels

*P. aeruginosa* intracellular ATP levels were determined as described previously [4,31] using an ATP determination kit (Invitrogen, Life Technologies, Carlsbad, CA, USA). Bacterial culture strain ATCC 27853 was grown to the mid-log phase (OD_600_ = 0.7), washed, and resuspended in the same volume of PBSM (1X PBS, 1mM MgCl_2_). The bacterial culture was treated with sub-inhibitory concentrations of compounds (1/8th, 1/16th and 1/25th MIC) for 3 h at 37 °C. After treatment, the cells were lysed in chloroform which was subsequently removed by boiling at 80 °C. The persistent ATP from cell lysate was measured in a 96-well black flat-bottom plate by measuring the luminescence. Luminescence was converted to concentration from standard curve. A culture treated with CCCP (12.5, 6.25 and 4 μg/mL) and azithromycin (12.5, 6.25 and 4 μg/mL) was included as positive and negative controls. Error bars represent the standard deviation of triplicates.

### 3.9. Determination of Frequency of Resistance (FoR) Levofloxacin Resistance in P. aeruginosa

Frequency-of-resistance studies were carried out as described previously [4,31].

### 3.10. Identification of Mutation by Whole-Genome Sequencing

DNA extraction, library preparation and whole-genome sequencing of *P. aeruginosa* parent strains ATCC 27853 and DA7232, and single isolates EPIR32, EPIR36 and EPIR38, was performed by CD Genomics (New York, NY, USA) using Illumina. Illumina sequencing reads were mapped using the published genome of *P. aeruginosa* ATCC 27853 (GenBank accession number CP011857), or the published genome of *P. aeruginosa* PAO1 (parent strain of DA7232) as reference genomes with the BWA-MEM tool from the Galaxy web platform (https://usegalaxy.org/, accessed on 1 November 2020 [31]. Variations in the genomes between resistant strains and parent strains were identified using the LoFreq tool from the same platform with the version available at the time. Sequencing data have been deposited in a public repository, and accession numbers will be provided upon manuscript acceptance.

### 3.11. Time-Kill Studies

Time-kill studies were carried out as described previously [4,31] with the following changes. When indicated, levofloxacin was added to the prepared bacterial suspensions once at the MIC (2 μg/mL). TXA11114 was added to bacterial suspensions at 1/16th, 1/25th, and 1/32nd times the MIC (6.25 μg/mL, 4 μg/mL, and 3.125 μg/mL, respectively).

### 3.12. Determination of Post-Antibiotic Effect (PAE) by Measuring Turbidity

Exponentially grown *P. aeruginosa* ATCC27853 was incubated with levofloxacin (1× MIC) alone, and combined with 6.25 μg/mL of TXA11114 for 1 h at 37 °C [87]. Treated cultures suspensions were diluted 50-fold to eliminate any drug carry over. Prior to growth resumption, untreated cell concentration was adjusted to equal treated samples to minimize difference in inoculum size. An aliquot of 200 µL was loaded to a 96-well flat-bottom microtiter plate in duplicate sets and optical density was measured periodically at 15 min intervals. PAE was derived from PAE = T_50_ − C_50_, where T_50_ and C_50_ are the time in hours required for the drug-treated and untreated cultures, respectively, to reach a value of OD_600nm_ corresponding to 50% of the final absorbance reached by an untreated control [84].

### 3.13. Ion Channel Method

The automated whole-cell patch-clamp (Qpatch HT) technique is used to record depolarizing currents, hNav1.5 and hCav1.2, and repolarizing potassium currents, hERG. Cells Recombinant HEK-293 cells stably transfected with human Nav1.5 cDNA, Recombinant HEK293 cell line expressing the human Cav1.2 (L-type voltage-gated calcium channel, hCav1.2 α1C/β2a/α2δ1, and recombinant CHO-K1 cells stably transfected with human hERG cDNA are used separately in each of these assays. The cells are harvested by Accutase and maintained in Serum-Free Medium at room temperature before assay. On the instrument, the cells are pipetted into each well of a 48-well plate in external solution. Test concentration stock solution is prepared in DMSO at 300× the final assay concentrations, and stored at −20 °C until the day of assay. On the day of the assay, an aliquot of the stock solution is thawed and diluted into external solution to make final test concentrations. A final concentration of 0.33% DMSO is maintained for each concentration of the assay compounds and controls. The assay is conducted at room temperature. Recording conditions hNav1.5 Peak current Onset and steady state block of peak Nav1.5 current are measured using a pulse pattern, repeated every 5 sec, consisting of a hyperpolarizing pulse to −120 mV for a 200 ms duration, depolarization to −15 mV amplitude for a 40 ms duration, followed by a step to 40 mV for 200 ms and finally a 100 ms ramp (1.2 V/s) to a holding potential of −80 mV. hCav1.2 currents are evoked following a 100 ms pulse to −60 mV followed by a 50 ms pulse to +10 mV before returning to the holding potential of −90 mV. This paradigm is delivered three times once every 20 s. Cells are held at −90 mV with a 5 s pulse to −60 mV every 20 s for a total of 120 s between each set of three pulses. The cell is held at −80 mV. Then the cell is depolarized to +40 mV for 500 ms and then to −80 mV over a 100 ms ramp to elicit the hERG tail current. This paradigm is delivered once every 8 s. The Extracellular Solution (control) is applied first and the cell is stabilized in the solution for 5 min. Then the test compound is applied from low to high concentrations sequentially on the same cell. The cells are incubated with each test concentration for 5 min. Reference compounds Tetracaine Nifedipine and E-4031 are tested concurrently for hNav1.5, hCav1.2 and hERG, respectively, at multiple concentrations to obtain an IC_50_ value.

The amplitude of the sodium current is calculated by measuring the difference between the peak inward current on stepping to −10 mV (i.e., peak current) and the leak current. The sodium current is assessed in vehicle control conditions and then at the end of each five (5)-minute compound application. Individual cell results are normalized to the vehicle control amplitude and the mean ± SEM is calculated for each compound concentration. These values are then plotted and estimated IC_50_ curve fits are calculated. The maximum inward current elicited on stepping to +10 mV for 50msec from −60 mV is measured. Compound interaction is assessed by dividing post-current amplitude (at the end of each three (3)-minute compound application) by the vehicle control amplitude and the mean calculated for each compound concentration. These values are then plotted and estimated IC_50_ curve fits are calculated. The percent inhibition of the hERG channel is calculated by comparing the peak (hERG tail) current amplitude before and after application of the compound (the current difference is normalized to vehicle control values).

### 3.14. Pharmacokinetic Evaluation of TXA11114 Alone and in Combination with Levofloxacin in a Murine P. aeruginosa Lung Infection Model

The lung PK levels of TXA11114 and levofloxacin were evaluated at the University of North Texas Health Science Center (UNTNHS). The *P. aeruginosa* strain UNT034-1 (ATCC 27853) was used for infection studies. Female CD-1 mice (6–8 weeks old; 22 ± 2 g) were obtained from Envigo Laboratories (Indianapolis, IN, USA). All procedures were conducted in accordance with protocols approved by the University of North Texas Health Science Center (UNTHSC) Institutional Animal Care and Use Committee (protocol IACUC-2021-0003). Neutropenia was induced by intraperitoneal (i.p.) injection of cyclophosphamide (Cytoxan) at 150 mg/kg on day 4 and 100 mg/kg on day 1 before infection. Mice were anesthetized by i.p. injection of ketamine (40 mg/kg) and xylazine (6 mg/kg) and intranasally inoculated with 0.05 mL of the prepared *P. aeruginosa* suspension by placing droplets onto the nares and allowing inhalation. Following inoculation, mice were returned to cages for recovery. At 2 h post-infection, mice received a single dose of TXA11114 intravenously (30 mg/kg), levofloxacin subcutaneously (30 mg/kg), or both agents in combination (30 + 30 mg/kg). At predetermined time points (0.083, 0.25, 0.5, 1, 2, 4, 8, and 24 h), groups of three mice were euthanized by CO_2_ asphyxiation. Blood was collected by cardiac puncture into tubes containing EDTA or serum separator gel, centrifuged at 4 °C, and plasma or serum was harvested within 30 min of collection. Bronchoalveolar lavage fluid (BALF) was collected to determine drug concentrations at the site of infection. Briefly, 1.2 mL of sterile 0.9% NaCl was instilled into the lungs via the trachea and gently withdrawn. The epithelial lining fluid (ELF) dilution factor was determined by the blood urea nitrogen (BUN) method. Plasma and BALF samples were stored at −80 °C until analysis. Drug concentrations were quantified by LC-MS/MS using a Surveyor HPLC system coupled to a TSQ Quantum Ultra triple-quadrupole mass spectrometer (Thermo Scientific). Separation was achieved on a Phenomenex Kinetex PFP column (50 × 2.1 mm, 2.6 µm) with a mobile phase gradient of water (0.1% formic acid, A) and acetonitrile (0.1% formic acid, B) at 0.5 mL/min. The gradient proceeded from 10% to 90% B over 4 min, held for 1 min, and re-equilibrated to initial conditions. The column oven was maintained at 30 °C. Detection was performed in positive-ion SRM mode monitoring the transitions *m*/*z* 373.2 → 238.0 (TXA11114), *m*/*z* 361.7 → 318.2 (levofloxacin), and *m*/*z* 331.8 → 288.1 (ciprofloxacin, internal standard). Calibration curves were matrix-matched in blank mouse plasma (0.19–100 µg/mL for TXA11114 and 0.78–100 µg/mL for levofloxacin) and BALF (0.19–25 µg/mL and 0.78–25 µg/mL, respectively). Samples (50 µL plasma or BALF) were spiked with 10 µL of internal standard in methanol, precipitated with 150 µL acetonitrile, vortexed, sonicated for 10 min, and centrifuged at 14,500 rpm for 5 min. A 100 µL aliquot of the supernatant was transferred to autosampler vials, and 10 µL was injected for analysis. Concentration-time data were analyzed using noncompartmental methods (PK Solutions v2.0; Summit Research Services, Montrose, CO, USA). Parameters including area under the concentration-time curve (AUC_0_-t), maximum concentration (C_max_), time to C_max_ (T_max_), apparent volume of distribution (V_d_), systemic clearance (CL), and terminal half-life (t_½_) were calculated. Data are presented as means ± standard deviation (SD) for three animals per time point.

### 3.15. Evaluation of EPI–Levofloxacin Combination Efficacy in Murine Infection Models

The efficacy of TAXIS EPIs in combination with levofloxacin was evaluated at the University of North Texas Health Science Center (UNTNHS) using established murine thigh and lung infection models of *P. aeruginosa* ATCC 27853 (UNT034-1). Female CD-1 mice (6–8 weeks old; 22 ± 2 g) were obtained from Envigo Laboratories, quarantined for at least 3 days, and housed 5 per cage in accordance with the approved IACUC protocol (IACUC-2017-0049). Neutropenia was induced by intraperitoneal (IP) administration of cyclophosphamide (Cytoxan) at 150 mg/kg on day 4 and 100 mg/kg on day 1 prior to infection.

#### 3.15.1. Thigh Infection Model

Mice were infected intramuscularly (IM) in the right thigh with 0.1 mL of bacterial suspension containing approximately 1 × 10^5^–10^6^ CFU/mL. Two hours post-infection, test articles were administered either intravenously (IV; EPIs) or subcutaneously (SC; levofloxacin). At 2 h or 24 h post-infection, mice were euthanized by CO_2_ asphyxiation, and thighs were aseptically excised and placed in 2 mL sterile phosphate-buffered saline (PBS) on ice. Tissue homogenization was performed using a Polytron PT10-35 (12 mm aggregate, Kinematica, Malters, Switzerland).

Homogenates (200 µL) were serially diluted 10-fold in sterile PBS across 96-well plates, and 8 µL aliquots were spotted onto BHI-charcoal agar, BHI containing 4 µg/mL levofloxacin (to assess resistance development), and citrate agar (for *P. aeruginosa* isolation). Plates were incubated overnight at 37 °C, and colonies were enumerated the following day.

#### 3.15.2. Lung Infection Model

For the lung infection model, mice were anesthetized by IP injection of 0.15 mL of a ketamine (40 mg/kg) + xylazine (6 mg/kg) mixture and inoculated intranasally (IN) with 0.05 mL of a 4 × 10^8^ CFU/mL bacterial suspension applied dropwise to the external nares. Two hours post-infection, mice received test articles IV (EPIs) or SC (levofloxacin). At 2 h or 24 h post-infection, mice were euthanized by CO_2_ asphyxiation, and lungs were aseptically removed, placed in 2 mL sterile PBS, and homogenized using the same procedure described above. Homogenates were serially diluted and plated (8 µL spots) onto BHI-charcoal and citrate agar, incubated overnight at 37 °C, and colonies were counted the following day.

Prior to combination testing, a dose-ranging study with levofloxacin alone was performed to identify a suboptimal or minimally efficacious dose for use in combination efficacy studies.

### 3.16. ADME Studies with TXA11114 (Conducted by Sai Life Sciences Ltd.)

#### 3.16.1. Aqueous Solubility

The aqueous solubility of TXA11114 was evaluated in system solution (SS) at pH 7.4. Known concentrations of TXA11114, prepared as DMSO stock solutions, were spiked into SS and incubated for 6 h at room temperature with constant shaking (250 rpm). Caffeine and diethylstilbestrol (DES) were used as positive controls. A 20 mM DMSO stock solution of TXA11114 and control compounds was diluted 100-fold in 1 × SS to achieve a final concentration of 200 µM, with the final DMSO concentration maintained at ≤1% to minimize solvent effects on solubility. Aliquots (500 µL) of diluted test and control compounds were dispensed into 96-deep-well plates in triplicate, sealed, and incubated as described above. Following incubation, 300 µL samples were withdrawn and filtered using 0.22 µm filter plates. Optical density at λ_max was measured using a UV spectrophotometer. Solubility (µM) was calculated using the formula:

Aqueous solubility (µM) = 2 × (OD_sample/OD_reference) × (reference concentration, 33.4 µM).

#### 3.16.2. Cytochrome P450 Inhibition

Inhibition of CYP1A2, CYP2D6, CYP2C9, CYP2C19, and CYP3A4 by TXA11114 was assessed in human liver microsomes by measuring the inhibition of probe substrate metabolism. Phenacetin, bufuralol, diclofenac, S-mephenytoin, and midazolam were used as probe substrates for CYP1A2, CYP2D6, CYP2C9, CYP2C19, and CYP3A4, respectively. Corresponding metabolites (acetaminophen, OH-bufuralol, 4-OH diclofenac, 4-OH mephenytoin, and 1-OH midazolam) were quantified by LC-MS/MS. Selective inhibitors (fluvoxamine (CYP1A2), quinidine (CYP2D6), sulfaphenazole (CYP2C9), ticlopidine (CYP2C19), and ketoconazole (CYP3A4)) were used as positive controls. Test and control compounds, prepared in DMSO at various concentrations, were spiked into 2× microsomal reaction mixtures, and 50 µL aliquots were transferred to individual wells. Probe substrates (25 µL, 4× concentration) were added to the respective wells and pre-incubated for 5 min at 37 °C. Reactions were initiated by the addition of 25 µL NADPH (4×) and incubated for 5 min (CYP3A4), 10 min (CYP1A2, CYP2D6, CYP2C9), or 20 min (CYP2C19). Reactions were quenched with 100 µL ice-cold acetonitrile containing glipizide as an internal standard. Plates were centrifuged at 4000 rpm for 15 min, and 100 µL supernatants were analyzed by LC-MS/MS in MRM mode. Percent activity was calculated as the ratio of metabolite peak area in the presence of test compound relative to the DMSO control, multiplied by 100. Percent inhibition was calculated as 100 − percent activity. IC_50_ values were determined using GraphPad Prism (version 5.02).

#### 3.16.3. Microsomal Stability

The metabolic stability of TXA11114 was evaluated in liver microsomes from human (HLM), dog (DLM), rat (RLM), and mouse (MLM). Test compound disappearance over time was monitored by LC-MS/MS. Verapamil (HLM and RLM) and imipramine (DLM and MLM) were used as positive controls. Microsomal incubations were prepared in 50 mM potassium phosphate buffer (pH 7.4) containing microsomes at a final concentration of 0.25 mg/mL. TXA11114 and control compounds (1 mM DMSO stocks) were spiked into the incubation mixtures to achieve a final concentration of 1 µM. Aliquots (70 µL) were transferred to 96-well reaction plates and pre-incubated at 37 °C for 5 min. Reactions were initiated by adding 30 µL NADPH (final concentration 1 mM). Control incubations lacking NADPH were included to assess non-enzymatic degradation. Reactions were terminated at 0, 5, 15, 30, and 60 min by the addition of 100 µL ice-cold acetonitrile containing glipizide as an internal standard. Plates were centrifuged at 4000 rpm for 15 min, and supernatants were analyzed by LC-MS/MS in MRM mode to monitor parent compound disappearance.

#### 3.16.4. Cytotoxicity

The cytotoxicity of TXA11114 was evaluated in HEK293T and A549 cell lines using the CellTiter-Glo™ assay (Promega, Madison, WI, USA). HEK293T cells (human embryonic kidney cells transformed with sheared adenovirus 5 DNA and expressing SV40 large T antigen) and A549 cells (human alveolar basal epithelial adenocarcinoma cell line; hypotriploid with a modal chromosome number of 66) were obtained from Sai Life Sciences Ltd. (Hyderabad, India). These cell lines are well-characterized and consistent with commonly used reference lines (e.g., A549, ATCC CCL-185).

TXA11114 was tested in a dose–response format with a top concentration of 20 µM using 2-fold serial dilutions in triplicate. Doxorubicin and staurosporine were included as reference controls. Cells (1200 cells/well in 27 µL complete medium) were seeded into 384-well plates and incubated overnight at 37 °C with 5% CO_2_. The following day, cells were treated with 3 µL of 10× serially diluted compounds. After a 24 h incubation, CellTiter-Glo reagent was added, and plates were gently shaken for 15–20 min at room temperature. Luminescence was measured using an EnVision multimode plate reader (PerkinElmer, Thane, India). Cell viability was calculated relative to 0.1% DMSO controls, and IC_50_ values were determined using GraphPad Prism.

#### 3.16.5. cLogP and PSA

cLogP and topological polar surface area (tPSA) were calculated in silico using standard cheminformatics algorithms.

#### 3.16.6. Protein Binding

Plasma protein binding was determined experimentally using equilibrium dialysis, with compound concentrations in plasma and buffer quantified by LC-MS/MS.

#### 3.16.7. Plasma Stability

Plasma stability was assessed by incubating the compound in plasma at 37 °C and monitoring parent compound disappearance over time by LC-MS/MS. Half-life values were calculated assuming first-order kinetics.

### 3.17. Synthetic Procedures and Characterization Data

#### General Methods

All Chemicals and solvents were used as received from the vendors without prior treatment or purifications. The analysis process was thorough and comprehensive. Unless otherwise stated, thin-layer chromatography (TLC) was done on 0.25 nm thick precoated silica gel 60 (HF-254, Whatman, Maidstone, Kent, UK). Flash column chromatography (FCC) was performed using Kieselgel 60 (32–63 mesh, Scientific Adsorbents, Atlanta, GA, USA). Elution for FCC usually employed a stepwise solvent polarity gradient correlated with TLC mobility. Chromatograms were observed under UV (short and long wavelength) light and/or were visualized by heating plates that were exposed to iodine/silica mix, dipped in a basic potassium permanganate, KMnO_4_ stain solution, dipped in phosphomolybdic acid stain solution, or dipped in alcoholic Ninhydrin solution. ^1^H NMR and ^13^C and ^19^F NMR Spectra were recorded using Mercury Varian 300 MHz instrument (300 MHz, 75 MHz, and 282 MHz, respectively, Varian, Inc., Palo Alto, CA, USA) using CDCl_3_, Pyridine-*d_5_*, MeOH-*d_4_*, or DMSO-*d_6_* solution with residual CDCl_3_, Pyridine-*d_5_*, MeOH-*d_4_*, or DMSO-*d_6_* as an internal standard. Chemical shifts are quoted in (δ, ppm) relative to the corresponding solvent peak and a coupling constant (*J*) is given in Hertz, multiplicity (s = singlet, d = doublet, t = triplet, q = quartet, m = multiplet). Data for ^13^C are reported as follows: chemical shift (δ, ppm), multiplicity (s = singlet, d = doublet, t = triplet, q = quartet, m = multiplet) and coupling constant *J* (Hertz, Hz). Low-resolution mass spectrometry was performed on the Shimadzu LC-MS system using a gradient mobile phase of 0.01% formic acid solution water/CH_3_CN. Analyses were performed using the Shimadzu technology LCMS 2020 system and the use of analytical column Phenomenex, 4.6 mm Gemini-NX 3u C18 110 A 50 × 4.6 mm). High-resolution mass spectrometry was performed on Thermo Scientific LTQ Orbitrap XL (Thermo Scientific, Waltham, MA, USA). Chromatography was performed at ambient temperature with run time = 6 min with a flow rate of 0.8 mL/min with linear gradient water (0.1% formic acid): CH_3_CN (0.1% formic acid [100:0] to water (0.1% formic acid): CH_3_CN (0.1% formic acid) [10:90] and resolved peaks detected by SPD-20AV photodiode array (PDA) Detector at 254, 280, and/or 215 nm and characterized by a low-resolution mass spectrometry instrument (Shimadzu, Kyoto, Japan) with an ESI ion source and positive mode ionization.

1. Synthesis of Intermediates **5**–**10**

Synthetic protocols for the amine intermediate **8** (di-*tert*-butyl ((4*R*)-5-amino-2-fluoropentane-1,4-diyl)dicarbamate)

To a solution of the known ester *tert*-butyl *(4S)*-4-(3-ethoxy-2-fluoro-3-oxopropyl)-2,2-dimethyloxazolidine-3-carboxylate (10.8 g, 33.8 mmol) in THF (150 mL) at room temperature was added LiBH_4_ (1.62 g, 74.4 mmol) portion-wise. The reaction mixture was stirred at room temperature overnight then quenched by the slow addition of acetone in portions and the resulting mixture was concentrated to give a residue. The residue was diluted with EtOAc and washed with H_2_O, saturated NaHCO_3_, and brine. The organic solution was dried over Na_2_SO_4_, filtered, and concentrated to give the crude product. The crude product was purified on silica gel and elution with 50% EtOAc/hexanes to afford the desired product (8.54 g, 91% yield). ^1^H NMR (300 MHz, CDCl_3_) *δ* 4.68 (dm, *J* = 54 Hz, 1H, CHF), 3.62–4.03 (m, 5H), 1.83–2.23 (m, 2H), 1.41–1.62 (m, 15H).

To a solution of the resulting alcohol from the previous step, *tert*-butyl (4*S*)-4-(2-fluoro-3-hydroxypropyl)-2,2-dimethyloxazolidine-3-carboxylate (6.36 g, 22.9 mmol) in dry THF (100 mL), was added triphenylphosphine (6.61 g, 25.2 mmol), phthalimide (3.71 g, 25.2 mmol), and DIAD (5.1 mL, 25.2 mmol) at 0 °C. The reaction mixture was stirred at 0 °C and gradually warmed up to room temperature overnight. The reaction mixture was concentrated and purified on column chromatography on silica gel using gradient elution 5–20% EtOAc/hexanes to afford the desired product (7.64 g, 82% yield) as a white solid. NMR values are listed for the major isomer ^1^H NMR (300 MHz, CDCl_3_) *δ* 7.85 (m, 2H), 7.72 (m, 2H), 4.87 (dm, *J* = 50.4 Hz, 1H, CHF), 4.02–3.67 (m, 5H), 2.25–1.89 (m, 2H), 1.53 (m, 6H), 1.45 (m, 9H). ^13^C NMR (75 MHz, CDCl_3_) *δ* 168.2, 152.5, 134.8, 132.2, 123.2, 93.3, 90.1 (d*, J* = 173.2 Hz), 80.5, 67.9, 56.1, 42.2, 36.7, 28.6, 27.8, 24.8.

To a solution of the corresponding phthalimide *tert*-butyl (4*S*)-4-(3-(1,3-dioxoisoindolin-2-yl)-2-fluoropropyl)-2,2-dimethyloxazolidine-3-carboxylate (6.3 g, 15.5 mmol) in MeOH (100 mL) was added hydrazine monohydrate (2.35 mL, 31.0 mmol). The mixture was stirred at room temperature overnight. The formed precipitate was filtered off and washed with CH_2_Cl_2_. The filtrate was concentrated and triturated with CH_2_Cl_2_. The solid was removed by filtration. The filtrate was washed with saturated NaHCO_3,_ brine and dried over Na_2_SO_4_, filtered, then concentrated and purified on silica gel column. Elution with EtOAc then 10% MeOH/CH_2_Cl_2_ with 1% NH_3_.H_2_O afforded the product (3.68 g, 86% yield) as a colorless gum. NMR values are listed for the major isomer, ^1^H NMR (300 MHz, MeOH-*d_4_*) *δ* 4.61 (dm, *J* = 50.7 Hz, 1H), 4.1 (m, 2H), 3.88 (m, 1H), 2.83 (m, 2H), 1.94 (m, 2H), 1.56 (s, 3H), 1.51 (m, 12H). ^13^C NMR (75 MHz, MeOH-d_4_) *δ* 154.0, 95.1 (d, *J* = 165 Hz), 94.5, 81.9, 69.2, 57.4, 47.2, 37.4, 28.9, 28.1, 25.0.

To a solution of the resulting amine from the previous step, *tert*-butyl (4*S*)-4-(3-amino-2-fluoropropyl)-2,2-dimethyloxazolidine-3-carboxylate (3.23 g, 11.7 mmol) in MeOH (50 mL), was added 4 N HCl solution in dioxane (11.7 mL, 46.8 mmol). The reaction mixture was stirred at 50 °C for 1 h then concentrated to give a residue. The residue was dissolved in MeOH/CH_2_Cl_2_ (10 mL/100 mL), then TEA (6.5 mL, 46.8 mmol) and (Boc)_2_O (6.4 g, 29.3 mmol) were added, stirred at room temperature for 3 h, then concentrated to afford a residue. The residue was diluted with EtOAc and washed with H_2_O, 10% citric acid, saturated NaHCO_3_, and brine. The organic solution was dried over Na_2_SO_4_, filtered, and concentrated to give a crude product. The crude product was purified by column chromatography on silica gel using 50%EtOAc/hexanes to afford the desired product (2.36 g, 60% yield) as a white solid. NMR values are listed for the major isomer, ^1^H NMR (300 MHz, CDCl_3_) *δ* 5.04 (m, 2H), 4.65 (dm, *J* = 51 Hz, 1H, CHF), 3.82 (m, 1H), 3.61 (m, 2H), 3.40 (m, 1H), 3.19 (m, 1H), 2.78 (m, 1H), 1.78-1.61 (m, 2H), 1.40 (s, 18H). ^13^CNMR (75 MHz, CDCl_3_) *δ* 156.3, 91.0 (d, *J* = 168.5 Hz), 80.0, 65.6, 49.6, 44.9 (d, *J* = 20 Hz), 34.4 (d, *J* = 19.5 Hz), 28.5.

To a solution of resulting alcohol 5 di-*tert*-butyl ((*4R*)-2-fluoro-5-hydroxypentane-1,4-diyl)dicarbamate (2.0 g, 5.90 mmol), triphenylphosphine (1.71 g, 6.54 mmol) and phthalimide (0.96 g, 6.54 mmol) in THF (30 mL) was added DIAD (1.32 mL, 6.54 mmol) at 0 °C. The reaction mixture was stirred at 0 °C then at room temperature overnight. The reaction mixture was concentrated and purified on column chromatography on silica gel using 5–20% EtOAc/hexanes to give the desired product (2.54 g, 91% yield) as a white solid. ^1^H NMR (300 MHz, CDCl_3_) δ 7.83 (m, 2H), 7.70 (m, 2H), 4.82 (m, 2H), 4.70 d (m, *J* = 48 Hz, 1H, CHF), 4.20 (bs, 1H), 3.74 (m, 2H), 3.45 (m, 1H), 3.24 (m, 1H), 1.86 (m, 2H), 1.42 (s, 9H), 1.22 (s, 9H). ^1^H NMR (75 MHz, CDCl_3_) *δ* 168.5, 156.1, 155.7, 134.1, 132.2, 123.5, 90.4 (d, *J* = 169 Hz), 79.79, 79.5, 47.0, 44.7 (d, *J* = 21.2 Hz), 42.4, 35.3 (d, *J* = 20.2 Hz), 28.5, 28.2.

**Figure 11:**
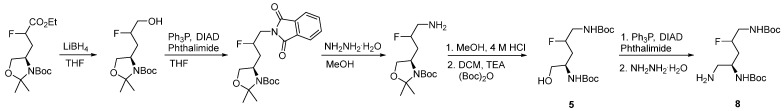
Synthetic route to access fluorinated triamine **8**.

To a solution of the resulting phthalimide from the previous step, di-*tert*-butyl ((4*S*)-5-(1,3-dioxoisoindolin-2-yl)-2-fluoropentane-1,4-diyl)dicarbamate (2.2 g, 4.73 mmol) in MeOH (40 mL), was added hydrazine monohydrate (0.72 mL, 9.46 mmol). The mixture was stirred at room temperature overnight and the precipitate formed was filtered off and washed with CH_2_Cl_2_. The filtrate was concentrated and diluted with CH_2_Cl_2_, washed with saturated NaHCO_3,_ brine and dried over Na_2_SO_4._ The organic solution was filtered and concentrated to give a crude product. The crude product was purified on silica gel column chromatography. Elution with EtOAc then 10% MeOH/CH_2_Cl_2_ with 1% NH_3_.H_2_O afforded product **8,** (Figure 11) (1.30 g, 82% yield) as a colorless gum. ^1^H NMR (300 MHz, CDCl_3_) d 4.55 (dm, *J* = 54 Hz, 1 h, CHF), 3.74 (bs, 1H), 3.25 (m, 2H), 2.65 (m, 2H), 1.86–1.66 (m, 2H), 1.44 (s, 18H).

**Figure 12:**
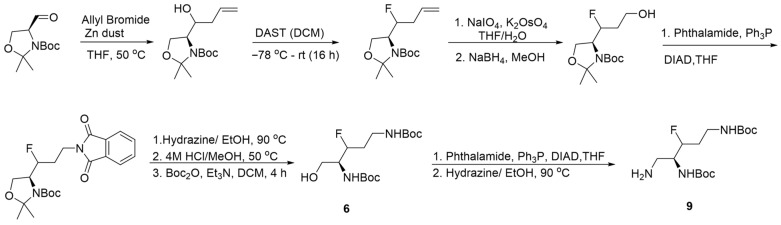
Synthetic route to access fluorinated triamine **9**.
*tert*-butyl (4*S*)-4-(1-hydroxybut-3-en-1-yl)-2,2-dimethyloxazolidine-3-carboxylate (Figure 12, step 1)

Synthetic protocols for the synthesis of amine intermediate **9** (di-*tert*-butyl ((4*S)*-5-amino-3-fluoropentane-1,4-diyl)dicarbamate) (Figure 12)

To a solution of Garner’s Aldehyde (1.2 g, 5.2 mmol) in anhydrous THF (20 mL) was added at 0 °C allyl bromide (540 µL, 6.2 mmol), activated zinc dust (400 mg, 6.2 mmol) and LiCl (260 mg, 6.2 mmol). The reaction mixture was heated to 50 °C and stirred at this temperature for another 6 h. The reaction mixture was filtered over a pad of celite, and the resulting filtrate was mixed with ethanol amine (6.2 mmol). The resulting white precipitate was filtered over a short pad of silica and the resulting filtrate was concentrated under vacuum; the resulting crude was purified using the ISCO flash chromatography system and the use of gradient elution of hexanes/EtOAc (R*_f_* = 0.19 (10% EtOAc/hexane) to give 910 mg of heavy clear oil 65% yield. The isolated alcohol was collected as a 1:1 diastereomeric mixture; the ^1^HNMR analysis shows that the diastereomeric mixture also exists as a mixture of rotamers. The listed ^1^HNMR values are for the major diastereomer/rotamer. ^1^HNMR (300 MHz, CDCl_3_) *δ* 5.82 (m, 1H), 5.06 (m, 2H), 3.89 (m, 4H), 2.14 (m, 2H), 1.42 (m, 15H). ^13^CNMR (75 MHz, CDCl_3_) *d* 153.8, 135.3, 117.3, 94.2, 80.8, 71.9, 64.4, 61.8, 37.9, 28.4, 26.6.

*tert*-butyl (4*S*)-4-(1-fluorobut-3-en-1-yl)-2,2-dimethyloxazolidine-3-carboxylate (Figure 12, step 2)

To a solution of the *tert*-butyl (4*S*)-4-(1-hydroxybut-3-en-1-yl)-2,2-dimethyloxazolidine-3-carboxylate (900 mg, 3.3 mmol) in anhydrous DCM (20.0 mL) was added DAST (890 µL, 6.6 mL) at −78 C. The reaction mixture was stirred at this temperature for 1 h then gradually warmed up to rt and stirred at this temperature overnight. The reaction mixture was then treated with sat. solution of NaHCO_3_ (30 mL) and the reaction mixture was transferred into a separatory funnel. The organic layer was washed with brine, and dried over anhydrous Na_2_SO_4_, filtered and evaporated to dryness. The crude material was purified using the ISCO flash chromatography system using gradient hexanes/EtOAc elution. The target was collected as a clear oil (575 mg, 64%). NMR analysis showed that the compound exists as a mixture of rotamers; the listed values represent the major rotamer. ^1^HNMR (300 MHz, CDCl_3_) *δ* 5.80 (m, 1H), 5.09 (m, 2H), 4.76 (dm, *J* = 48 Hz, 1H, CHF), 4.08–3.85 (m, 3H), 2.35 (m, 2H), 1.45 (m, 15H). ^13^CNMR (75 MHz, CDCl_3_) *δ* 153.0, 133.2, 118.4, 94.1, 91.6 (d, *J* = 173.3 Hz, CHF), 80.8, 63.2, 59.6, 37.0 (d, *J* = 26.7 Hz), 28.6, 27.0, 24.9. ^19^FNMR (282 MHz, CDCl_3_) *δ* −193.85 (major rotamer).

(2*S*)-2-amino-3-fluorohex-5-en-1-ol

In an effort to establish the diastereomeric ratio of the fluorinated derivative from the previous reaction, a (27.3 mg, 0.1 mmol) of the fluoro-alkene derivative produced in the previous step was treated with 4M HCl (500 µL), the mixture was stirred at 45 °C for 1 h, then the volatiles were removed under vacuum and the crude material was dissolved in THF (2 mL) and treated with activated zinc dust (20 mg, 0.3 mmol). The reaction mixture was heated at 65 °C for an additional hour, then the reaction mixture was filtered over a short pad of celite, the filtrate was evaporated to dryness, and the resulting solid was analyzed as a crude mixture to establish the ratio which found almost 4:1; the listed values are for the major isomer. ^1^HNMR (300 MHz, MeOH-*d_4_*) *δ* 5.90 (m, 1H), 5.18 (m, 2H), 4.75 (dm, *J* = 46.8 Hz, 1H), 3.94 (m, 1H), 3.67 (m, 2H), 3.23 (m, 1H), 2.51 (m, 2H).^13^CNMR (75 MHz, MeOH-*d_4_*) *δ* 134.0, 119.0, 94.0 (d, *J* = 172.7 Hz, CHF), 60.7, 56.4 (d, *J* = 21.0 Hz), 36.8 (d, *J* = 21.1 Hz).

*tert*-butyl (4*S*)-4-(1-fluoro-3-hydroxypropyl)-2,2-dimethyloxazolidine-3-carboxylate (Figure 12, step 3)

To a solution of the *tert*-butyl (4*S*)-4-(1-fluorobut-3-en-1-yl)-2,2-dimethyloxazolidine-3-carboxylate from the previous step (330 mg, 1.2 mmol) in THF (35.0 mL) was added water (15.0 mL), NaIO_4_ (775.6 mg, 3.6 mmol) and K_2_OsO_2_·2H_2_O (9.0 mg, 0.025 mmol). The reaction mixture was stirred at 0 °C and left warming gradually to rt overnight. After 16 h standing at rt, the reaction mixture was treated with saturated solution of sodium sulfite (50 mL) and stirred vigorously at rt for 2 h, then the mixture was transferred to a separatory funnel and mixed with EtOAc (50 mL); the aqueous layer was extracted with EtOAc (25 mL × 3). The combined organic layer was washed with brine (50 mL), dried over anhydrous Na_2_SO_4_, filtered, and evaporated under vacuum; the crude material was used in the next step without further purification. NMR values are listed for the crude material without purification. ^1^HNMR (300 MHz, CDCl_3_) *δ* 9.77 (m, 1H), 5.05 (dm, *J* = 45 Hz, 1H, CHF), 4.08–3.92 (m, 3H), 2.82 (m, 2H), 1.45 (m, 15H). ^13^CNMR (75 MHz, CDCl_3_) *δ* 198.6, 158.9, 94.4, 87.7 (d, *J =* 172.5 Hz, CHF), 81.2, 64.1, 59.4 (d, *J =* 27.8 Hz), 46.5 (d, *J =* 21.2 Hz), 28.5, 27.5, 24.5. The crude aldehyde from the previous step was dissolved in methanol (20.0 mL) and treated with NaBH_4_ (133.2 mg, 3.6 mmol). The reaction mixture was then stirred at rt for 30 min before it was quenched with a saturated solution of ammonium chloride. The aqueous layer was extracted with EtOAc (25 mL × 3) and the organic layer was washed with brine (25 mL), dried over anhydrous Na_2_SO_4_, filtered, and evaporated under vacuum. The resulting crude material was used in the next step without further purification. The primary alcohol was collected as clear oil (290 mg, 87% over two steps). The NMR values are listed for the major rotamer. ^1^HNMR (300 MHz, CDCl_3_) *δ* 4.81 (dm, *J* = 46.2 Hz, 1H), 4.07 (m, 2H), 3.91 (m, 2H), 3.80 (m, 2H), 1.86 (m, 2H), 1.46 (m, 15H). ^13^CNMR (75 MHz, CDCl_3_) *δ* 153.0, 94.1, 90.9 (d, *J* = 178.8 Hz, CHF), 81.1, 63.5, 59.1, 35.0 (d, *J* = 22.2 Hz), 29.8 (d, *J*= 17.7 Hz) 28.6, 27.2, 24.8. ^19^FNMR (282 MHz, CDCl_3_) *δ* −194.23 (major rotamer).

*tert*-butyl (4*S*)-4-(3-(1,3-dioxoisoindolin-2-yl)-1-fluoropropyl)-2,2-dimethyloxazolidine-3-carboxylate (Figure 12, step 4)

To a solution of the *tert*-butyl (4*S*)-4-(1-fluoro-3-hydroxypropyl)-2,2-dimethyloxazolidine-3-carboxylate (190.0 mg, 0.68 mmol) in dry THF (10 mL) was added triphenylphosphine (359.0 mg, 1.37 mmol) and phthalimide (201.0 mg, 1.37 mmol). The reaction mixture was cooled down to 0 C using an ice bath. After 15 min, a solution of DIAD (276.0 mg, 1.37 mmol) in anhydrous THF (5 mL) was introduced dropwise to the reaction mixture over 30 min. Upon complete addition of the DIAD solution, the reaction mixture was left to warm up to rt and stirred for two hours. The reaction progress was monitored by TLC or LCMS; when the reaction came to completion, the reaction mixture was treated with MeOH (5.0 mL), and the volatiles were removed under vacuum; the crude mixture was loaded to a pre-backed RediSep column and purified using flash chromatography on an ISCO machine using gradient elution of EtOAc/hexane to collect the target phthalimide 225 mg, 82% yield. The NMR analysis showed that the compound exists as a mixture of rotamers; the listed values are for the major rotamer. ^1^HNMR (300 MHz, CDCl_3_) *δ* 7.71 (m, 2H), 7.59 (m, 2H), 4.63 (dm, *J* = 46.8 Hz, 1H),3.96–3.75 (m, 5H), 1.87 (m, 2H), 1.36 (m, 12H), 1.29 (m, 3H). ^13^CNMR (75 MHz, CDCl_3_) *δ* 168.0, 152.7, 134.0, 132.0, 123.3, 93.9, 90.6 (d, *J* = 173.8 Hz, CHF), 80.6, 63.2, 59.6 (d, *J* = 24.5 Hz), 34.7, 31.0 (d, *J* = 20.4 Hz) 28.3, 27.0, 24.5. ^19^FNMR (282 MHz, CDCl_3_) *δ* −194.11 (major rotamer).

di-*tert*-butyl ((4*S*)-3-fluoro-5-hydroxypentane-1,4-diyl)dicarbamate (Figure 12, step 5)

To a solution of the *tert*-butyl (4*S*)-4-(3-(1,3-dioxoisoindolin-2-yl)-1-fluoropropyl)-2,2-dimethyloxazolidine-3-carboxylate (225 mg, 0.55 mmol) in EtOH (10.0 mL) was added hydrazine hydrate (83 µL, 1.66 mmol). The reaction mixture was heated and stirred at 90 °C for 2 h; the reaction progress was monitored by LCMS. Once the starting material was consumed entirely based on the LCMS analysis, the reaction was stopped by evaporating all volatiles under a vacuum. Then, the resulting free amine was treated with 4M HCl/MeOH (4.0 mL) to cleave the acid-labile-protecting groups. The reaction mixture was stirred at 50 °C for two hours. Then, the volatiles were evaporated entirely under a high vacuum for 2 h. The crude mixture was then suspended in DCM (20 mL) and treated with Boc_2_O (355 mg, 1.66 mmol) and Et_3_N (460 µL, 3.36 mmol). The reaction mixture was stirred at rt for three hours. Then, the reaction mixture was diluted with 20 mL of DCM and transferred into a separatory funnel; the organic layer was washed with 1 M NaOH solution (20 mL), saturated NH_4_Cl solution (20 mL), and brine (20 mL). The organic layer was dried over Na_2_SO_4_, filtered, and concentrated under a vacuum. The resulting crude material was loaded into a RediSep silica column and purified by flash chromatography using an ISCO machine and a gradient elution of EtOAc/hexane. The final compound was collected after evaporating the desired fractions as a thick, clear gummy material in a 95% yield over a three-step sequence. In some cases, the final compound solidifies on standing as white crystals. NMR analysis showed that the compound exists as a mixture of diastereomers; the listed values are for the major diastereomer. ^1^HNMR (300 MHz, CDCl_3_) *δ* 5.32 (m, 2H), 4.94 (m, 1H), 4.58 (dm, *J* = 45.9 Hz, 1H), 3.72 (m, 2H), 3.23 (m, 2H), 1.85 (m, 2H), 1.36 (m, 18H). ^13^CNMR (75 MHz, CDCl_3_) *δ* 168.4, 156.2, 156.0, 91.6 (d, *J* = 172 Hz, CHF), 79.8, 79.3, 6.7 (d, *J* = 4 Hz), 54.5 (d, *J* = 24.8Hz), 37.2, 32.1 (d, *J* = 19.7Hz), 28.4, 28.3. ^19^FNMR (282 MHz, CDCl_3_) *δ* −191.23 (major diastereomer). HRMS *m*/*z* calculated for C_15_H_30_FN_2_O_5_ [M+H] 337.2139 found 337.2146.

di-*tert*-butyl ((4*S*)-5-(1,3-dioxoisoindolin-2-yl)-3-fluoropentane-1,4-diyl)dicarbamate (Figure 12, step 6)

To a solution of the di-tert-butyl ((4*S*)-3-fluoro-5-hydroxypentane-1,4-diyl)dicarbamate (370 mg, 1.1 mmol) in dry THF (10.0 mL) was added triphenylphosphine (577 mg, 2.2 mmol) and phthalimide (323 mg, 2.2 mmol). The reaction mixture was cooled down to 0 °C using an ice bath. After 15 min, a solution of DIAD (444.4 mg, 2.2 mmol) in anhydrous THF (2.0 mL) was introduced dropwise to the reaction mixture over 30 min. Upon complete addition of the DIAD solution, the reaction mixture was left to warm up to rt and stirred for another two hours. The reaction progress was monitored by TLC or LCMS; when the reaction came to completion, the reaction mixture was treated with MeOH (5.0 mL), and the volatiles were removed under vacuum; the crude mixture was dissolved in 2 mL of DCM and loaded to a pre-backed RediSep column and purified using flash chromatography on an ISCO machine using gradient elution of EtOAc/hexane to collect the target phthalimide (114 mg, 85% yield as white solid). ^1^HNMR (300 MHz, CDCl_3_) *δ* 7.82 (m, 2H), 7.69 (m, 2H), 5.02 (m, 2H), 4.62 (dm, *J* = 48.5 Hz, 1H), 4.05 (m, 1H), 3.85 (m, 2H), 3.31 (m, 1H), 1.95 (m, 2H), 1.41 (m, 9H) 1.16 (m, 9H). ^13^CNMR (75 MHz, CDCl_3_) *δ* 168.6, 156.1, 155.7, 134.2, 132.2, 123.5, 93.4 (d, *J* = 174.8 Hz, CHF), 80.0, 79.6, 52.5 (d, *J* = 26.4 Hz), 37.5 (d, *J* = 26.4 Hz), 32.5 (d, *J* = 26.3 Hz), 28.6, 28.2.

di-*tert*-butyl ((4*S*)-5-amino-3-fluoropentane-1,4-diyl)dicarbamate **9** (Figure 12, step 7)

To a solution of the corresponding phthalimide derivative from the previous step (465 mg, 1.0 mmol) in EtOH (20 mL)) was added hydrazine (150 µL, 3.0 mmol). The reaction mixture was stirred at 90 °C for two hours. Then, the volatiles were removed under a vacuum, and the crude material was dissolved in a minimum amount of 10% DCM/MeOH solution and loaded into a RediSep column. The material was then purified using flash chromatography using an ISCO machine and gradient elution of MeOH/DCM. The desired fractions were collected and evaporated to dryness to give a white solid with a 69% yield (over two steps). A 10 mg sample was dissolved in deuterated MeOH and analyzed by NMR spectroscopy to show one diastereomer. ^1^HNMR (300 MHz, MeOH-*d_4_*) *δ* 4.60 (dm, *J* = 46.6 Hz, 1H), 3.77 (m, 1H), 3.34 (m, 2H), 3.02 (m, 1H), 2.82 (m, 1H), 1.91 (m, 2H), 1.61 (m, 9H), 1.58 (m, 9H). ^13^CNMR (75 MHz, MeOH-*d_4_*) *δ* 158.5, 158.3, 93.5 (d, *J* = 171.1 Hz, CHF), 80.5, 80.1, 57.1 (d, *J* = 23.6 Hz), 42.3, 37.9, 33.5 (d, *J* = 21 Hz), 29.0, 28.9. ^19^FNMR (282 MHz, MeOH-d4) *δ* −192.94. LRMS *m*/*z* calculated for C_15_H_31_FN_3_O_4_ [M + H] 336.2299 found 336.2308.

**Figure 13:**
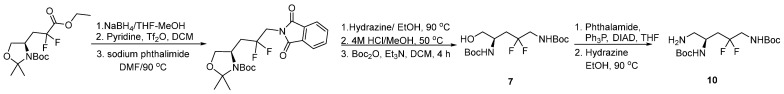
Synthetic protocols for the synthesis of difluoro triamine derivative **10**.

Synthetic protocols for the synthesis of the amin-difluoro derivative 10 (di-*tert*-butyl (5-amino-2,2-difluoropentane-1,4-diyl)(*R*)-dicarbamate)
*tert*-butyl (*R*)-4-(3-(1,3-dioxoisoindolin-2-yl)-2,2-difluoropropyl)-2,2-dimethyloxazolidine-3-carboxylate (Figure 13, step 1, via triflate intermediate)

To a solution of ester 4 *tert*-butyl (*R*)-4-(3-ethoxy-2,2-difluoro-3-oxopropyl)-2,2-dimethyloxazolidine-3-carboxylate (1.012 g, 3.0 mmol) in 5:1 solution of THF/MeOH (20 mL) was added at 0 °C NaBH_4_ (555 mg, 15 mmol). The reaction mixture was stirred at this temperature for 30 min and then warmed up gradually to rt and the stirring continued for another 3 h. Upon the complete conversion based on the TLC, the reaction mixture was treated with NH_4_Cl saturated solution at 0 °C and the reaction mixture was stirred for an additional hour; the solid precipitate was filtered off and the filtrate was transferred to a separatory funnel and extracted with EtOAc (25 mL × 3). The combined organic layer was washed with brine, dried over Na_2_SO_4_, filtered, and evaporated under vacuum. The resulting crude was passed through a short silica column with EtOAc elution. The excess EtOAc was evaporated, and the crude alcohol was used in the next step without purification. ^1^HNMR (300 MHz, C_6_D_6_) *d* 3.91 (m, 2H), 3.66 (m, 3H), 2.57–2.08 (m, 2H), 1.45 (s,3H), 1.41 (s, 3H), 1.27 (s, 9H).

To a solution of the corresponding alcohol from the previous step *tert*-butyl *(R)*-4-(2,2-difluoro-3-hydroxypropyl)-2,2-dimethyloxazolidine-3-carboxylate (500 mg, 1.67 mmol) in DCM 2.0 mL was added pyridine (157 µL, 1.99 mmol) at 0 °C, followed by dropwise addition of Tf_2_O solution (336 µL, 1.99 mmol) in 2.0 mL of DCM. The reaction mixture was stirred at 0 °C for 30 min, warmed up gradually to rt, and left stirring at this temperature for another 45 min. The reaction mixture was mixed with 10 mL DCM and 1M HCl (10 mL), and the organic layer was then washed with water and brine, dried over Na_2_SO_4_, filtered, and evaporated to dryness. The resulting triflate was used without further purification in the next step. To a solution of phthalimide (49 mg, 0.33 mmol) in anhydrous DMF (2 mL) was added sodium hydride, NaH (60% dispersed on mineral oil) (13.2 mg, 0.33 mmol); the reaction mixture was stirred under N_2_ gas at rt for 45 min. The formed sodium phthalimide was transferred using a cannula to the triflate derivative made in the previous step (119 mg, 0.27 mmol) in DMF (2.0 mL), and the reaction mixture was then heated to 125 °C under N_2_ gas for 24 h. Upon completion and consumption of all the triflate starting material based on the LCMS monitoring, the reaction mixture was cooled down to rt and mixed with water 10 mL and EtOAc (10 mL). The reaction mixture was transferred into a separatory funnel; the aqueous layer was extracted by EtOAc (10 mL × 3). The combined organic layer was washed with 1 M HCl (10 mL) and brine (10 mL), dried over Na_2_SO_4_, filtered, and evaporated under vacuum to give the crude product, which was subjected to FCC using an ISCO chromatography system and EtOAc/hexane mobile phase. The desired phthalimide product was collected as a white solid (41 mg, 35%) R*_f_* = 0.25 (20%EtOAC/hexane). NMR analysis showed that the compound exists as a mixture of rotamers; the listed spectroscopic values are for the major rotamer. ^1^HNMR (300 MHz, CDCl_3_) *δ* 7.87 (m, 2H), 7.74 (m, 2H), 4.24–3.96 (m, 3H), 3.91 (t, *J* = 9.0 Hz, 2H), 2.52–2.21 (m, 2H), 1.55 (m, 3H), 1.46 (m, 9H), 1.37 (m, 3H). ^13^CNMR (75 MHz, CDCl_3_) *δ* 167.6, 154.0, 152.0, 134.6, 132.0, 123.9, 121.4 (t, *J* = 242 Hz, *CF_2_*). 93.7, 80.7, 80.3, 68.0, 52.6, 42.6, 38.7, 28.6, 27.0, 23.3.

di-*tert*-butyl (2,2-difluoro-5-hydroxypentane-1,4-diyl)*(R)*-dicarbamate **7** (Figure 13, step 2)

To a solution of the *tert*-butyl (*R*)-4-(3-(1,3-dioxoisoindolin-2-yl)-2,2-difluoropropyl)-2,2-dimethyloxazolidine-3-carboxylate (240 mg, 0.54 mmol) in EtOH (10.0 mL) was added hydrazine hydrate (70 µL, 1.13 mmol). The reaction mixture was heated and stirred at 90 C for 2 h; the reaction progress was monitored by LCMS. Once the starting material was consumed entirely based on the LCMS analysis, the reaction was stopped by evaporating all volatiles under a vacuum. Then, the resulting free amine was treated with 4M HCl/MeOH (5.0 mL) to cleave the acid-labile-protecting groups. The reaction mixture was stirred at 50 °C for three hours. Then, the volatiles were evaporated entirely under a high vacuum for 2 h. The crude mixture was then suspended in DCM (20 mL) and treated with Boc_2_O (480 mg, 2.2 mmol) and Et_3_N (610 µL, 4.4 mmol). The reaction mixture was stirred at rt for 4h. Then, the reaction mixture was diluted with 20 mL of DCM and transferred into a separatory funnel; the organic layer was washed with 1 M NaOH solution (20 mL), saturated NH_4_Cl solution (20 mL), and brine (20 mL). The organic layer was dried over Na_2_SO_4_, filtered, and concentrated under a vacuum. The resulting crude material was loaded into a RediSep silica column and purified by flash chromatography using an ISCO machine and a gradient elution of EtOAc/hexane. The final compound was collected after evaporating the desired fractions as a thick, clear gummy material in a 50% yield over a three-step sequence. In some cases, the final compound solidifies on standing as white crystals. ^1^HNMR (300 MHz, CDCl_3_) *δ* 5.22 (m, 1H), 5.13 (m, 1H), 3.91 (m, 1H), 3.62 (m, 2H), 3.48 (m, 2H), 2.95 (m, 1H), 2.12 (m, 2H), 1.40 (m, 18H). ^13^CNMR (75 MHz, CDCl_3_) *δ* 156.1, 122.7 (t, *J =* 241.7 Hz*, CF_2_*), 80.5, 80.1, 65.1, 48.1, 45.2 (t, *J =* 31.3 Hz, CH_2_-CF_2_), 35.2 (t, *J* = 23.18 Hz, CH_2_-CF_2_), 28.54, 28.47. ^19^FNMR (282 MHz, CDCl_3_) *δ* −101.85 HRMS *m*/*z* calculated for C_15_H_29_F_2_N_2_O_5_ [M+H] 355.2045 found 355.2053.

*tert*-butyl *(R)*-4-(3-(1,3-dioxoisoindolin-2-yl)-2,2-difluoropropyl)-2,2-dimethyloxazolidine-3-carboxylate (Figure 13, step 3, via Mitsunobu reaction)

To a solution of the di-*tert*-butyl (2,2-difluoro-5-hydroxypentane-1,4-diyl)(*R*)-dicarbamate (100 mg, 0.28 mmol) in dry THF (5.0 mL) was added triphenylphosphine (148 mg, 0.565 mmol) and phthalimide (84 mg, 0.565 mmol). The reaction mixture was cooled down to 0 °C using an ice bath. After 15 min, a solution of DIAD (114.1 mg, 0.565 mmol) in anhydrous THF (2.0 mL) was introduced dropwise to the reaction mixture over 30 min. Upon complete addition of the DIAD solution, the reaction mixture was left to warm up to rt and stirred for another two hours. The reaction progress was monitored by TLC or LCMS; when the reaction came to completion, the reaction mixture was treated with MeOH (5.0 mL), and the volatiles were removed under vacuum. The crude mixture was dissolved in 2 mL of DCM and loaded to a pre-backed RediSep column and purified using flash chromatography on an ISCO machine using gradient elution of EtOAc/hexane to collect the target phthalimide (114 mg, 85% yield as white solid). ^1^HNMR (300 MHz, CDCl_3_) *δ* 5.7.81 (m, 2H), 7.68 (m, 2H), 5.15 (m, 1H), 5.06 (m, 1H), 4.24 (m,1H), 3.79 (m, 2H), 3.53 (m, 2H), 2.13 (m, 2H), 1.39 (m, 9H), 1.23 (m, 9H). ^13^CNMR (75 MHz, CDCl_3_) *δ* 168.5, 156.6, 155.6, 134.2, 132.3, 123.4, 122.4 (t, *J* = 240 Hz*, CF_2_*), 80.5, 79.8, 45.7, 45.3 (t, *J* = 30 Hz, CH_2_-CF_2_), 42.2, 36.7 (t, *J =* 24.8 Hz, CH_2_-CF_2_), 28.5, 28.3.

di-*tert*-butyl (5-amino-2,2-difluoropentane-1,4-diyl)(*R*)-dicarbamate) **10** (Figure 13, step 4)

To a solution of the di-*tert*-butyl (5-(1,3-dioxoisoindolin-2-yl)-2,2-difluoropentane-1,4-diyl) (*R*)-dicarbamate (100 mg, 0.2 mmol) in EtOH (10 mL)) was added hydrazine (30 µL, 0.6 mmol). The reaction mixture was stirred at 90 °C for two hours. Then, the volatiles were removed under a vacuum, and the crude material was dissolved in a minimum amount of 10% DCM/MeOH solution and loaded into a RediSep column. The material was then purified using flash chromatography using an ISCO machine and gradient elution of MeOH/DCM. The desired fractions were collected and evaporated to dryness to give a white solid with a 95% yield. A 10 mg sample was dissolved in deuterated MeOH and analyzed by NMR spectroscopy. ^1^HNMR (300 MHz, MeOH-*d_4_*) *δ* 3.86 (m, 1H), 3.49 (m, 2H), 2.66 (m, 2H), 2.04 (m, 2H), 1.46 (m, 18H). ^13^CNMR (75 MHz, MeOH-*d_4_*) *δ*, 158.4, 158.1, 124.0 (t, *J* = 241.5 Hz, CF_2_), 80.7, 80.3, 49.5, 47.0, 45.9 (t, *J* = 30.8 Hz), 37.5 (t, *J* = 24.8 Hz), 28.9, 28.8. ^19^FNMR (282 MHz, CDCl_3_) *δ* −103.42. HRMS *m*/*z* calculated for C_15_H_30_F_2_N_3_O_4_ [M+H] 354.2204 found 354.2212.

2. Synthesis of Intermediates **11**

**Figure 14:**
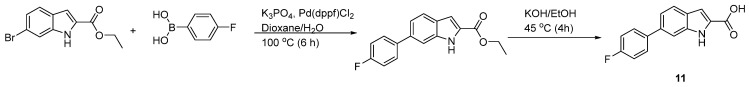
Synthetic route for the carboxylic acid derivative **11**.

Synthetic protocols for the carboxylic acid **11** (6-(4-fluorophenyl)-1*H*-indole-2-carboxylic acid)
ethyl 6-(4-fluorophenyl)-1*H*-indole-2-carboxylate (Figure 14, step 1)

In a clean 250 mL sealed tube charged with stir bar was added Ethyl 6-bromo-1*H*-indole-2-carboxylate (804.3 mg, 3.0 mmol), dioxane (20 mL), (4-fluorophenylboronic acid) (840 mg, 6.0 mmol), K_3_PO_4_ (1908 mg, 9.0 mmol), and water (10.0 mL). The reaction mixture was degassed for 30 min by passing N_2_ gas through the solution. After that, Pd(dppf)Cl_2_ (122 mg, 0.15 mmol) was added and the reaction vessel was sealed and refluxed at 100 °C for 6 h. Then, the reaction mixture was cooled down to rt and the volatiles were concentrated under vacuum. The resulting crude was redissolved with 50% EtOAc/hexane. And the mixture was then filtered through a short pad of silica. The organic layer was concentrated and the crude mixture was purified by flash chromatography using gradient elution of EtOAc/hexane. The titled compound was collected as yellow solid with a 68.5% yield (*R_f_* = 0.43, 10% EtOAc/hexane). ^1^HNMR (300 MHz, CDCl_3_) *δ* 9.21 (s, 1H), 7.72 (m, 1H), 7.56 (m, 3H), 7.34 (m, 1H), 7.24 (m, 1H), 7.13 (m, 2H), 4.43 (m, 2H), 1.43 (t, *J* = 7.11 Hz, 3H). ^13^CNMR (75 MHz, CDCl_3_) *δ* 162.6 (d, *J* = 245 Hz*, Sp2-C-F*), 162.2, 138.0, 137.6, 129.1 (d, *J =* 8.0 Hz), 128.3, 128.5, 127.0, 123.1, 121.0, 115.8 (d, *J =* 21 Hz), 110.2, 108.8, 61.3, 14.6.

6-(4-fluorophenyl)-1*H*-indole-2-carboxylic acid 11(Figure 14, step 2)

To a solution of the ethyl 6-(4-fluorophenyl)-1*H*-indole-2-carboxylate (283 mg, 1.0 mmol) in ethanol (8.0 mL) and water (4.0 mL) was added KOH (112.2 mg, 2.0 mmol); the reaction mixture was stirred at 45 °C for 4 h, at which time all starting material disappeared. The reaction mixture was then treated with a saturated solution of NaHSO_4_ to adjust the pH of the solution to pH = 7.0. The formed precipitate was collected by vacuum filtration, washed with cold water and the collected solids were dried further inside an oven at 55 °C. The free acid was collected as white solid in 82%. ^1^HNMR (300 MHz, MeOH-*d_4_*) *δ* 7.67 (m, 4H), 7.33 (dd, *J* = 8.4, 1.5 Hz, 1H), 7.18 (m, 3H). ^13^CNMR (75 MHz, MeOH-*d_4_*) *δ* 165.2, 163.8 (d, *J* = 243 Hz*, Sp2-C-F*), 139.7, 139 (d, *J* = 87 Hz), 130.1 (d, *J* = 7.8 Hz), 128.2, 123.7, 121.2, 116.6 (d, *J* = 21.8 Hz), 111.4, 109.2. ^19^FNMR (282 MHz, MeOH-*d_4_*) *δ* −118.62.

3. Synthesis and Characterization Data of TXA Compounds

Synthetic protocols for amide TXA11114: (*N-((2R*)-2,5-diamino-4-fluoropentyl)-6-(4-fluorophenyl)-1*H*-indole-2-carboxamide)

To a solution of 6-(4-fluorophenyl)-1*H*-indole-2-carboxylic acid (420 mg, 1.64 mmol) in DMF (10 mL) was added DIPEA (0.53 mL, 3.00 mmol), HOBt (120 mg, 0.89 mmol), and EDC (342 mg, 1.80 mmol). The reaction mixture was stirred at room temperature, then di-*tert*-butyl ((4*R*)-5-amino-2-fluoropentane-1,4-diyl)dicarbamate (500 mg, 1.50 mmol) was added and the reaction was continued to stir at room temperature overnight. The reaction mixture was diluted with EtOAc. The combined organic layer was washed with water and brine, then dried over anhydrous sodium sulfate and filtered. The filtrate was then concentrated and purified by column chromatography on silica gel using 20–30% ethyl acetate in hexanes to give the product di-*tert*-butyl ((4*R*)-2-fluoro-5-(6-(4-fluorophenyl)-1*H*-indole-2-carboxamido)pentane-1,4-diyl)dicarbamate (690 mg, 81% yield) as a white solid. ^1^H NMR (300 MHz, CDCl_3_) *δ* 9.21 (bs, 1H), 7.67 (d, *J* = 8.4 Hz, 1H), 7.59-7.53 (m, 3H), 7.32 (dd, *J* = 8.4, 1.6 Hz, 1H), 7.12 (t, *J* = 8.7 Hz, 1H), 6.89 (m, 1H), 5.12 (d, *J* = 8.5 Hz, 1H, NH), 4.89 (m, 1H, NH), 4.75 (dm, *J* = 52.2 Hz, 1H, CHF), 4.09 (m, 1H), 3.543 (m, 2H), 3.28 (m, 2H), 1.87 (m, 2H), 1.42 (s, 9H), 1.39 (s, 9H). ^13^C NMR (300 MHz, CDCl_3_) *δ* 162.6 (d, *J* = 244 Hz, Sp2-CF), 162.2, 157.1, 156.3, 138.2, 137.3, 137.0, 131.4, 129.1 (d, *J* = 8.3 Hz), 127.3, 122.6, 120.8, 115.8 (d, *J* = 21.5 Hz), 110.2, 102.8, 90.9 (d, *J* = 168.8 Hz, CHF), 80.5, 80.1, 48.0, 44.8 (d, *J* = 16.7 Hz) 35.1 (d, *J* = 22.4 Hz), 28.5.

**Figure 15:**
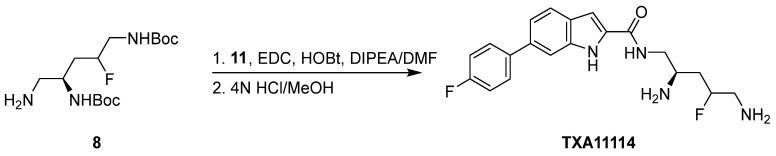
Synthesis of TXA11114.

To a solution of di-*tert*-butyl ((4*R*)-2-fluoro-5-(6-(4-fluorophenyl)-1*H*-indole-2-carboxamido)pentane-1,4-diyl)dicarbamate (670 mg, 1.19 mmol) in MeOH (10 mL) was added HCl solution (4 M in dioxane, 1.19 mL). It was stirred at rt overnight and the solvent was removed under vacuo. The residue was triturated with EtOAc and the product *N-((2R*)-2,5-diamino-4-fluoropentyl)-6-(4-fluorophenyl)-1*H*-indole-2-carboxamide: TXA11114 as an HCl salt was collected as a white powder (495 mg, 95% yield, HPLC purity: 98%). ^1^H NMR (300 MHz, CD_3_OD) *δ* 7.73–7.67 (m, 4H), 7.37 (dd, *J* = 8.4, 1.5 Hz, 1H), 7.22–7.16 (m, 3H), 5.20 (dm, *J* = 51 Hz, 1H, CHF), 3.85–3.65 (m, 3H), 3.44–3.26 (m, 2H), 2.27–2.06 (m, 2H). ^13^CNMR (75 MHz, Me3OD) *δ* 165.4, 163.9 (d, *J* = 143.2 Hz, sp^2^ CF), 139.8, 139.2, 138.2, 132.3, 130.1 (d, *J* = 8 Hz), 128.4, 123.5, 121.3, 116.6 (d, *J* = 21.5 Hz), 111.3, 105.4, 89.2 (d, *J* = 170 Hz, Sp3CF), 50.6, 44.2 (d, *J* = 20.3 Hz),42.9, 34.3 (d, *J* = 19.6 Hz). MS (ESI+): 396.25 [M + H]^+^ for C_22_H_26_FN_5_O (Figure 15).

Synthetic protocols for the amide TXA11164: (*N*-((2*S*)-2,5-diamino-3-fluoropentyl)-6-(4-fluorophenyl)-1*H*-indole-2-carboxamide)

**Figure 16:**
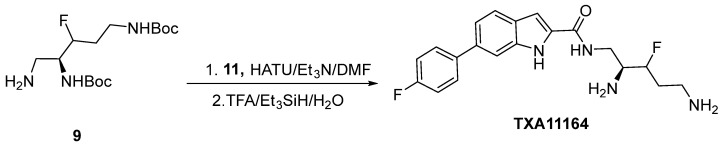
Synthesis of TXA11164.

To a solution of 6-(4-fluorophenyl)-1*H*-indole-2-carboxylic acid 11 (45 mg, 0.18 mmol) in DMF (2.0 mL) was added a coupling agent HATU (137 mg, 0.0.36 mmol) and di-*tert*-butyl ((4*S*)-5-amino-3-fluoropentane-1,4-diyl) dicarbamate (60 mg, 0.18 mmol). The reaction mixture was cooled down to 0 °C, and was then treated with Et_3_N (100 µL, 0.74 mmol). The reaction was stirred at 0 °C for 15 min, then allowed to warm up gradually to rt and stirred at ambient temperature for another 30 min. The reaction mixture was diluted with a saturated solution of NH_4_Cl (5.0 mL); the aqueous layer was extracted with EtOAc (10.0 mL × 3). The combined organic layer was washed with brine (5.0 mL), dried over Na_2_SO_4_, filtered, and evaporated under vacuum. The crude material was purified using an ISCO flash chromatography system and the use of gradient elution of hexane/EtOAc. The evaporation of the desired fractions gave the di Boc protected amide. The desired amide di-*tert*-butyl ((4*S*)-3-fluoro-5-(6-(4-fluorophenyl)-1*H*-indole-2-carboxamido)pentane-1,4-diyl)dicarbamate was collected as white solid in 78% yield (80 mg), (Figure 16). ^1^HNMR (300 MHz, DMSO*d_6_*) *δ* 11.73 (s, 1H), 8.53 (m, 1H), 7.71 (m, 3H), 7.65 (s, 1H), 7.35 (m, 3H), 7.16 (s, 1H), 6.94 (m, 2H), 4.55 (dm, *J* = 46.6 Hz, 1H, CHF), 3.87 (m, 1H), 3.49 (m, 2H), 3.09 (m, 2H), 1.74 (m, 2H), 1.40 (m, 9H) 1.37 (m, 9H). ^13^CNMR (75 MHz, DMSO*d_6_*) *δ* 161.5 (d, *J* = 242.2 Hz, Sp2-CF), 161.3, 155.5, 155.4, 137.7, 137.0, 134.6, 132.4, 128.6 (d, *J* = 8.0 Hz), 126.5, 121.9, 119.2, 115.7 (d, *J* = 21.0 Hz), 110.0, 102.5, 91.9 (d, *J* = 170.2 Hz, CHF), 78.1, 77.5, 53.0 (d, *J* = 23.1 Hz), 36.3, 31.8 (d, *J* = 19.3 Hz), 28.2, 28.0.

The resulting amide from the previous step (80 mg, 0.136 mmol) was treated with 95% TFA/2.5% Et_3_SiH, 2.5% H_2_O solution (1.5 mL). The reaction mixture was stirred at rt for 45 min, then the volatiles were removed under vacuum to give the product *N-((2S)*-2,5-diamino-3-fluoropentyl)-6-(4-fluorophenyl)-1*H*-indole-2-carboxamide: TXA11164 as a TFA salt in 82% yield (HPLC purity: 93%). ^1^HNMR (300 MHz, MeOH-*d_4_*) *δ* 7.78 (m, 4H), 7.44 (d, *J* = 8.9 Hz, 1H), 7.26 (m, 3H), 5.18 (dm, *J* = 48.3 HZ, CHF), 4.01-3.78 (m, 3H), 3.39 (m, 2H), 2.36 (m, 2H). ^13^CNMR (75 MHz, MeOH-*d_4_*) *δ* 165.3, 163.8 (d, *J* = 243.2 Hz, Sp2-CF), 139.7 (d, *J* = 3.2 Hz), 138.2, 132.2, 130.0 (d, *J* = 7.6 Hz), 128.4, 123.4, 121.3, 116.5 (d, *J* = 21.0 Hz), 111.3, 105.5, 91.7 (d, *J* = 173.0, CHF), 55.4 (d, *J* = 20.1 Hz), 38.5, 37.6, 29.9 (d, *J* = 20.7 Hz). ^19^FNMR (282 MHz, MeOH-*d_4_*) *δ* −118.83, −197.73. HRMS *m*/*z* calculated for C_20_H_23_F_2_N_4_O [M + H] 373.1840 found 373.1835, (Figure 16).

Synthetic protocols for amide TXA12027 (*R*)-*N*-(2,5-diamino-4,4-difluoropentyl)-6-(4-fluorophenyl)-1*H*-indole-2-carboxamide

**Figure 17:**
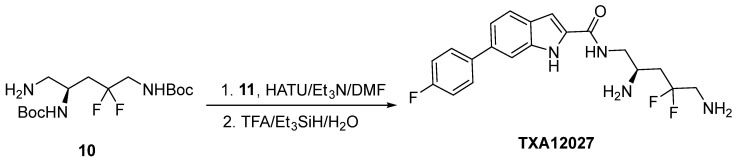
Synthesis of TXA12027.

To a solution of 6-(4-fluorophenyl)-1*H*-indole-2-carboxylic acid 11 (15 mg, 0.06 mmol) in DMF (1.0 mL) was added a coupling agent HATU (22.5 mg, 0.06 mmol) and di-*tert*-butyl (5-amino-2,2-difluoropentane-1,4-diyl) (*R*)-dicarbamate (19 mg, 0.05 mmol). The reaction mixture was cooled down to 0 °C, and was then treated with Et_3_N (15 µL, 0.11 mmol). The reaction was stirred at 0 °C for 15 min, then allowed to warm up gradually to rt and stirred at ambient temperature for another 30 min. The reaction mixture was diluted with a saturated solution of NH_4_Cl (2.0 mL); the aqueous layer was extracted with EtOAc (5.0 mL × 3). The combined organic layer was washed with brine (2.0 mL), dried over Na_2_SO_4_, filtered, and evaporated under vacuum. The crude material was purified using an ISCO flash chromatography system and the use of gradient elution of hexane/EtOAc. The evaporation of the desired fractions gave the di Boc protected amide. The desired amide di-*tert*-butyl (2,2-difluoro-5-(6-(4-fluorophenyl)-1*H*-indole-2-carboxamido)pentane-1,4-diyl)(*R*)-dicarbamate was collected as white solid in 85% yield (25 mg), (Figure 17). ^1^HNMR (300 MHz, CDCl_3_) *δ* 9.87 (m, 1H), 7.52 (m, 5H), 7.29 (d, *J* = 8.3 Hz, 1H), 7.08 (t, *J* = 8.7, 2H), 6.95 (m, 1H), 5.66 (m, 1H), 5.06 (m, 1H), 4.18 (m, 1H), 3.54 (m, 4H), 2.12 (m, 2H), 1.40 (m, 9H) 1.35 (m, 9H). ^13^CNMR (75 MHz, CDCl_3_) *δ* 162.6, 162.5 (d, *J* = 244.5 Hz, Sp2-CF), 156.9, 156.2, 138.2 (d, *J* = 3.2 Hz), 137.2 (d, *J* = 9.9 Hz), 131.3, 129.0 (d, *J* = 7.9 Hz), 127.2, 122.5, 121.6 (t, *J* = 240.0 Hz), 120.7, 115.8 (d, *J* = 18.5 Hz), 110.4, 103.2, 80.7, 80.4, 46.6, 45.4, 45.3, 36.3 (t, *J* = 23.3 Hz), 28.5, 28.4.

The resulting amide from the previous step (25 mg, 0.045 mmol) was treated with 95% TFA/2.5% Et_3_SiH, 2.5% H_2_O solution (0.5 mL). The reaction mixture was stirred at rt for 45 min, then the volatiles were removed under vacuum to give the product *(R)-N-*(2,5-diamino-4,4-difluoropentyl)-6-(4-fluorophenyl)-1*H*-indole-2-carboxamide TXA12027 as a TFA salt (HPLC purity: 89%). ^1^HNMR (300 MHz, MeOH-*d_4_*) *δ* 7.67 (m, 4H), 7.33 (dd, *J* = 1.3, 8.3 Hz, 1H), 7.28 (s, 1H), 7.16 (t, *J* = 8.8, 2H), 3.96 (m, 1H), 3.76 (m, 4H), 2.64 (m, 2H). ^13^CNMR (75 MHz, MeOH-*d_4_*) *δ* 165.5, 163.8 (d, *J* = 243 Hz, Sp2-CF), 139.7 (d, *J* = 3.2 Hz), 139.2, 138.2, 132.2, 130.0 (d, *J* = 7.7 Hz), 128.4, 122.5, 123.5, 122.0 (t, *J* = 243.5 Hz), 116.5 (d, *J* = 20.7 Hz), 111.3, 105.7, 48.2, 44.7 (t, *J* = 24.2 Hz), 43.2, 36.5 (t, *J* = 25.9 Hz). ^19^FNMR (282 MHz, MeOH-*d_4_*) *δ* −91.09, −77.42. HRMS *m*/*z* calculated for C_20_H_22_F_3_N_4_O [M + H] 391.1746 found 391.1747.

## 4. Conclusions

In pursuit of a safe and effective EPI to establish this adjuvant strategy as a viable therapeutic approach for treating *P. aeruginosa* infections, we designed, synthesized, and evaluated fluorine-substituted diamine-linked 2-carboxamide indole analogs. Among the three fluorinated derivatives prepared, only TXA11114 exhibited significant potentiation of levofloxacin activity. The difluoro analog was inactive, likely due to its substantially reduced amine pKa, while TXA11167 displayed a comparable pKa to TXA11114 but unexpectedly lacked potentiation activity, suggesting that factors beyond basicity influence EPI performance.

TXA11114 demonstrated strong potentiation against multiple MDR clinical isolates, rapid bactericidal activity in combination with levofloxacin, and an undetectable frequency-of-resistance emergence. Accumulation and genetic studies were consistent with an efflux-related mechanism without evidence of membrane perturbation. In addition, TXA11114 exhibited physicochemical and tolerability properties supportive of further development and showed a complementary pharmacokinetic profile with levofloxacin, resulting in robust *in vivo* efficacy in murine thigh and lung infection models.

Ongoing studies are evaluating pure diastereomeric analogs with variations in amine and fluorine stereochemistry to further optimize this scaffold and define its therapeutic potential as an adjunctive antibacterial agent.

## 5. Patents

Patent application WO2021243273 resulting from the work reported in this manuscript has been filed.

## Figures and Tables

**Table 1:** Comparison of potentiation abilities of fluorinated EPI analogs in combination with levofloxacin against *P. aeruginosa* ATCC 27853.

	TXA01182	TXA09155	TXA11114	TXA11164	TXA12027
EPI MIC (µg/mL)	100	50	100	>200	100
LVX * MIC (+EPI)	0.125	0.063	0.125	1	1
Fold MIC reduction [LVX/EPI-LVX]	8	16	8	1	1

* LVX: levofloxacin; EPIs tested at 6.25 µg/mL.

**Table 2:** Comparative potentiation of antimicrobial agents in *P. aeruginosa* by TAXIS EPIs TXA11114, TXA09155, and TXA01182.

Antibiotics	MIC (μg/mL), (Fold Difference)			
	No EPI	TXA11114 *	TXA09155 *	TXA01182 *
Aztreonam	8	4, (2)	2, (4)	2, (4)
Cefepime	2	0.5, (4)	0.5, (4)	1, (2)
Cefpirome	8	2, (4)	1, (8)	4, (2)
Ceftazidime	2	0.5, (4)	0.125, (16)	1, (2)
Ceftobiprole	8	2, (4)	1, (8)	8, (1)
Gentamicin	2	2, (1)	2, (1)	2, (1)
Imipenem ^#^	4	4, (1)	4, (1)	4, (1)
Levofloxacin	1	0.125, (8)	0.063, (16)	0.125, (8)
Cotrimoxazole	256	32, (8)	8, (32)	16, (>16)
Doxycycline	32	4, (8)	1, (32)	2, (16)
Minocycline	32	2, (16)	0.5, (64)	1, (32)
Chloramphenicol	>256	64, (>4)	16, (>16)	32, (>8)

^#^ Not a substrate for efflux pumps in *P. aeruginosa*; * EPIs tested at 6.25 µg/mL.

**Table 3:** Comparative levofloxacin potentiation study of TXA11114 and other known EPIs against multidrug-resistant *P. aeruginosa* clinical isolates from the CDC-FDA.

Strain	Levofloxacin MIC (μg/mL), (Fold Difference)					Resistance Mechanisms
	No EPI	+TXA11114 (6.25 μg/mL)	+TXA09155 (6.25 μg/mL)	+MC-04,124 (50 μg/mL)	+PAβN (50 μg/mL)	
AR-0229	64	2, (32)	2, (32)	64, (1)	64, (1)	*gyrA-T83I, nalC-G71E, mexR-V126Q, OXA-50, PAO*
AR-0232	8	0.5, (16)	0.5, (16)	8, (1)	0.5, (16)	*gyrA-T83I, nalC-G71E, mexR-V126Q, aadA6, OXA-50, PAO, strA, strB, sul1, tet(C)*
AR-0233	4	0.5, (8)	0.125, (32)	4, (1)	4, (1)	*catB7, OXA-50, PAO*
AR-0234	8	0.25, (32)	0.5, (16)	8, (1)	0.5, (16)	*gyrA-T83I, nalC-G71E, mexR-V126Q, aadA6, OXA-50, PAO, strA, strB, tet(C)*
AR-0239	32	8, (4)	4, (16)	64, (1)	8, (8)	*gyrA-T83I, nalC-G71E, mexR-V126Q, aac(6′)-IIa, aadB, aph(3′)-Ic, cmlA1, dfrB5, GES-1, OXA-10, OXA-50, strA, strB, tet(G), VIM-11*
AR-0242	8	2, (4)	1, (16)	4, (2)	0.5, (16)	*aac(3)-Id, aadA2, cmlA1, dfrB5, OXA-4, OXA-50, PAO, VIM-2*
AR-0244	64	8, (8)	2, (64)	64, (1)	64, (1)	*gyrA-T133H, nalC-G71E, mexR-V126Q, OXA-50*
AR-0246	64	8, (8)	8, (8)	64, (1)	64, (1)	*gyrA-T83I, nalC-G71E, mexR-V126Q, aadB, NDM-1, OXA-10, OXA-50, PAO, rmtD2, tet(G), VEB-1*
AR-0248	16	2, (8)	0.5, (32)	16, (1)	16, (1)	*aac(3)-Id, aadA2, cmlA1, dfrB5, OXA-4, OXA-50, PAO, tet(G), VIM-2*
AR-0249	64	4, (16)	2, (32)	64, (1)	8, (8)	*gyrA-T83I, nalC-G71E, aac(3)-Id, aadA2, cmlA1, dfrB5, OXA-4, OXA-50, PAO, tet(G), VIM-2*
AR-0252	32	4, (8)	0.5, (64)	32, (1)	1, (32)	*aadA1, aadA6, OXA-2, OXA-50, PAO, sul1*
AR-0253	2	0.125, (16)	0.031, (64)	2, (1)	0.031, (64)	*catB7, OXA-50, PAO*
AR-0264	64	4, (16)	1, (32)	64, (1)	64, (1)	*gyrA-D87Y, nalC-G71E, OXA-50, PAO*

Resistance mechanisms listed for each isolate were inferred from analysis of publicly available whole-genome sequences of the corresponding clinical isolates deposited in the CDC/FDA Antimicrobial Resistance Isolate Bank. Mutations and resistance genes were identified by the authors through inspection of the published genomic data rather than experimentally determined in the present study.

**Table 4:** Levofloxacin potentiation by TXA11114 against multidrug-resistant *P. aeruginosa* clinical isolates from Walter Reed Army Hospital in USA.

Strains	Levofloxacin MIC (μg/mL), (Fold Difference)		Fluoroquinolones and Efflux Resistance Mechanisms
	No EPI	+TXA11114 (6.25 μg/mL)	
MRSN #8130	16	2, (8)	*nalC-G71E, gyrA-D87N*
MRSN #994	16	2, (8)	*gyrA-T83I, mexR-V126E*
MRSN #1344	8	0.25, (16)	*mexR-V126E, nalC-G71E*
MRSN #3587	8	1, (8)	*nalC-LOF*
MRSN #3705	8	0.25, (32)	*nalC-LOF, parS-A115D*
MRSN #5498	32	4, (8)	*mexR-V126E, nalC-G71E*
MRSN #5519	64	4, (16)	*gyrA-T83I, mexR-V126E, parC-S87L*
MRSN #5539	16	8, (2)	*gyrA-T83I*
MRSN #6220	128	8, (16)	*gyrA-T83I, parC-S87L*
MRSN #6241	8	2, (4)	*mexR-V126E*
MRSN #6678	32	4, (8)	*gyrA-T83I, parC-S87L*
MRSN # 6695	8	0.5, (16)	*mexR-V126E, nalC-G71E, gyrA-T83I*
MRSN #8139	8	1, (8)	*nalC-G71E, gyrA-T83I*
MRSN #8912	16	2, (8)	*gyrA-T83I, nalC-A145V*
MRSN #8914	16	2, (8)	*gyrA-T83I, parE-S457G*
MRSN #8915	128	64, (2)	*gyrA-T83I, gyrA-D87N, parC-S87L, parS-L137P*
MRSN #14981	16	1, (16)	*mexR-V126E, nalC-G71E*
MRSN #15566	8	0.25, (32)	*mexR-V126E, nalC-G71E*
MRSN #16344	8	0.5, (16)	*mexR-V126E, nalC-G71E, gyrA-D87N*
MRSN #369569	32	4, (8)	*mexR-V126E, nalC-G71E, gyrA-T133M*

**Table 5:** Comparative study of antibiotic MIC potentiation by TXA11114 in efflux mutants.

Antibiotics	MIC Ratio of Antibiotics Alone/(Antibiotics + 6.25 µg/mL TXA11114)		
	K767 (WT)	K1455 (↑*mexAB-oprM*)	K1119 (∆*mexAB-oprM*)
Levofloxacin	8	16	2
Doxycycline	8	32	2
Chloramphenicol	4	16	2
Cefepime	8	16	2
Ceftazidime	4	8	2
Imipenem *	1	1	1

* Not a substrate for efflux pumps in *P. aeruginosa*. ↑ indicates overexpression of the MexAB–OprM efflux pump. ∆ indicates deletion of the indicated gene.

**Table 6:** Frequency of resistance to TXA11114 and levofloxacin.

*P. aeruginosa*	Levofloxacin 4× MIC (μg/mL)	TXA11114 (μg/mL)	Frequency of Resistance
ATCC 27853	4	0	3.80 × 10^−8^
		6.25	<1.04 × 10^−10^
CDC AR-0232	32	0	1.68 × 10^−8^
		6.25	<1.00 × 10^−10^
CDC AR-0248	64	0	8.64 × 10^−8^
		6.25	<1.47 × 10^−10^
CDC AR-0249	256	0	2.15 × 10^−8^
		6.25	<8.77 × 10^−11^

**Table 7:** Mutations involved in TXA11114 resistance in *P. aeruginosa*.

Resistance to	Parent Strain	FoR ^#^	Strain Name	Mutation	Gene Role
TXA11114 alone (4X-MIC)	*P. aeruginosa* ATCC 27853	<3.09 × 10^−9^	NA	NA	NA
TXA11114 alone (1X-MIC)		1.80 × 10^−6^	EPIR32	*ompH*-E63X *	Skp-like periplasmic chaperone
			EPIR36	*ompH*-Q127X *	
			EPIR38	*ompH*-Q70X *	
TXA11114 (¼X-MIC) + LVX (1X-MIC)	*P. aeruginosa* DA7232	<1.46 × 10^−9^	NA	NA	NA

^#^ FoR, frequency of resistance; * Termination codon.

**Table 8:** TXA11114 exhibits favorable physicochemical and ADME properties.

				Human	Dog		Rat	Mouse	
Protein Binding (%)				99.0	98.9		98.5	98.9	
Plasma stability t_1/2_ (min)				>120	>120		>120	>120	
Microsome Stability t_1/2_ (min)				>60	ND		>60	ND	
Compound	MW	Aqueous solubility (µM) at pH 7.4	cLogP	PSA	CYP Inhibition IC_50_ (µM)				
					1A2	2C9	2C19	2D6	3A4
TXA09155	366.19	155 ± 3	2.61	ND	>100	>100	>100	67.2	28.5
TXA11114	372.42	145.9 ± 6.1	2.34	93.17	>100	>100	>100	>100	63

**Table 9:** Cardiotoxicity monitoring of TXA11114 and TXA09155.

Compound	Concentration	Ion Channel	% Inhibition (n = 2)
TXA09155	10 µM	Nav1.5	0.1
		hERG	9.2
		Cav1.2	12.7
TXA11114		Nav1.5	−3.7
		hERG	10.9
		Cav1.2	0.2

**Table 10:** Initial nephrotoxicity screen for TXA11114.

	Blood Urea Nitrogen (mg/dL)	Creatine (mg/dL)
TXA11114	15.3	0.22
Normal Male Rat Mean	16.0	0.28

## Data Availability

The original contributions presented in this study are included in the article/Supplementary Material. Further inquiries can be directed to the corresponding author.

## Supplementary Materials

The following supporting information can be downloaded at: https://www.mdpi.com/article/10.3390/antibiotics15040346/s1, Table S1. Susceptibility of *P. aeruginosa* mutants resistant to TXA11114 combination to various antimicrobials; Table S2. Levofloxacin potentiation by TXA11114 in the parent strain and *ompH* mutant derivatives of *P. aeruginosa* ATCC 27853; Table S3. Mean bacterial thigh counts of *P. aeruginosa* ATCC 27853 following SC administration of levofloxacin w/wo IV dosing of TXA11114. Table S4. Mean bacterial lung counts of *P. aeruginosa* ATCC 27853 following SC administration of levofloxacin w/wo IV dosing of TXA11114. Table S5. Effect of higher *P. aeruginosa* inoculum on the levofloxacin potentiation by TXA11114. Supporting File S1: HPLC Spectrum and NMR Spectrum.

## Abbreviations

The following abbreviations are used in this manuscript:

MDR, multidrug-resistant; EPI, efflux pump inhibitor; MIC, minimal inhibitory concentration; FoR, frequency of resistance; MoA, mechanism of action; PK, pharmacokinetic; PD, pharmacodynamic; BALF, bronchoalveolar lavage fluid; TLC, thin-layer chromatography; LVX, levofloxacin; DXC, doxycycline; CAZ, ceftazidime; TGC, tigecycline; PMB, polymyxin B; AMK, amikacin; MEM, meropenem; AZM, azithromycin; AUC, area under the curve; ADME, absorption, distribution, metabolism, and excretion; MTD, maximum tolerated dose; CFUs, colony-forming units.
