# Supplementary material for: TXA11114: Discovery of an In Vivo Efficacious Efflux Pump Inhibitor (EPI) That Potentiates Levofloxacin Against Pseudomonas aeruginosa
Source: Antibiotics (Basel). 2026 Mar 27;15(4):346. doi: 10.3390/antibiotics15040346 (PMC13114161; doi:10.3390/antibiotics15040346)
Supplement: Supplementary file 1 [file antibiotics-15-00346-s001.zip › antibiotics-4207869-supplementary.pdf]

TXA11114: Discovery of an *in vivo* efficacious efflux pump inhibitor (EPI) that potentiates levofloxacin against *Pseudomonas aeruginosa*

Jesus D. Rosado-Lugo[‡], Pratik Datta[‡], Ahmad Altiti[‡], Yongzheng Zhang, Jun Lu, Yi Yuan and Ajit K. Parhi[*]

TAXIS Pharmaceuticals Inc., Monmouth Junction, NJ 08852, USA

[‡] These authors contributed equally to this work. * Correspondence:

aparhi@taxispharma.com

Supporting Information includes: susceptibility profiles of *P. aeruginosa* TXA11114-resistant mutants (Table S1); levofloxacin potentiation by TXA11114 in parent and *ompH* mutant strains (Table S2); mean bacterial thigh counts following levofloxacin with or without TXA11114 treatment (Table S3); mean bacterial lung counts following levofloxacin with or without TXA11114 treatment (Table S4); effect of bacterial inoculum on levofloxacin potentiation by TXA11114 (Table S5).

Supporting Information

Table S1. Susceptibility of *P. aeruginosa* mutants resistant to TXA11114 combination to various antimicrobials.

Strain	MIC (µg/mL)								
	TXA	LVX	DXC	CAZ	TGC	PMB	AMK	MEM	AZM
ATCC 27853[#]	100	1	32	2	8	2	8	1	64
EPIR32	200	0.25	2	0.25	2	4	2	0.125	32
EPIR36	200	0.125	1	0.125	2	4	1	0.125	16
EPIR38	200	0.5	8	2	8	4	4	0.5	64

[#] Parent strain, TXA: TXA11114; LVX: levofloxacin; DXC: doxycycline; CAZ: ceftazidime; TGC: tigecycline, PMB: polymyxin B; AMK: amikacin; MEM: meropenem AZM: azithromycin

Table S2. Levofloxacin potentiation by TXA11114 in the parent strain and *ompH* mutant derivatives of *P. aeruginosa* ATCC 27853

	ATCC 27853 (Parent Strain)	EPIR32	EPI36	EPI38
EPI MIC (µg/mL)	100	>200	>200	ND
LVX MIC (+ EPI 6.25 µg/mL)	0.125	0.25	0.125	ND
Fold MIC reduction [LVX/EPI-LVX]	8	1	1	ND

Table S3. Mean bacterial thigh counts of *P. aeruginosa* ATCC 27853 following SC administration of levofloxacin w/wo IV dosing of TXA11114.

Test Article	Treatment				Time post dose (hr)	Mean Log_{10} CFU	SD	Mean Log_{10} CFU Change vs			
	mg/kg / dose	mg/kg/day	Route	Regimen				+2 hr Control	+24 hr Control	LVX alone	TXA alone
TXA	30	120	IV	QID (q6hr)	+ 24 hr	8.95	0.09	2.87	-0.13		

LVX	10	40	SC			7.41	0.66	1.33	-1.41		
	15	60				5.97	0.72	-0.11	-2.85		
	20	80				4.47	0.82	-1.61	-4.35		
TXA + LVX	30 + 10	120 + 40	IV + SC			6.57	0.89	0.49	-2.25	-0.84	-2.38
	30 + 15	120 + 60				4.86	0.32	-1.22	-3.96	-1.11	-4.09
	30 + 20	120 + 80				3.27	0.38	-2.81	-5.55	-1.20	-5.68
Untreated Controls	NA	NA	NA	NA		8.82	0.2	2.74	0.00		
	NA	NA	NA	NA	+ 2 hr	6.08	0.09	0.00	-2.74		

TXA: TXA11114; LVX: levofloxacin; n = 5

Table S4. Mean bacterial lung counts of *P. aeruginosa* ATCC 27853 following SC administration of levofloxacin w/wo IV dosing of TXA11114.

Test Article	Treatment				Time post dose (hr)	Mean Log_{10} CFU	SD	Mean Log_{10} CFU Change vs	
	mg/kg/dose	mg/kg/day	Route	Regimen				+2 hr Control	+24 hr Control
TXA11114	30	120	IV	QID (q6hr)	+ 24 hr	8.75	0.19	4.16	-0.14
Levofloxacin	30	120	SC			3.98	0.59	-0.61	-4.91
	15	60				5.86	0.56	1.27	-3.03
TXA11114 + Levofloxacin	30 + 30	120 + 120	IV + SC			2.68	0.43	-1.91	-6.21
	30 + 25	120 + 100				4.02	0.97	-0.57	-4.87
	30 + 20	120 + 80				4.42	1.09	-0.17	-4.47
	30 + 15	120 + 60				5.68	1.22	1.09	-3.21
Untreated Controls	NA	NA	NA	NA		8.89	0.18	4.3	0
	NA	NA	NA	NA	+ 2 hr	4.59	1.26	0	-4.3

TXA: TXA11114; LVX: levofloxacin; n = 5

Table S5. Effect of higher *P. aeruginosa* inoculum on the levofloxacin potentiation by TXA11114.

	LVX MIC (μg/mL), (fold difference)	
P. aeruginosa OD_{600}	0.001*	0.025
TXA11114 (μg/mL)		

50	0.008, (128)	0.031, (64)
25	0.031, (32)	0.063, (32)
12.5	0.063, (16)	0.25, (8)
6.25	0.125, (8)	1, (2)
0	1	2

* Final OD_{600} needed to reach 1-5 x 10^5 colony forming units (CFU) per mL in accordance with CLSI methods [24].

Supporting File S1

HPLC Spectrum of TXA11114

==== Shimadzu LabSolutions Analysis Report ====

Acquired by: System Administrator<br>**Sample ID**: **TXA11114**<br>Tray#: 1<br>Vial#: 31<br>Injection Volume: 3 µL	Data File: C:\LabSolutions\Data\TXA\TXA11114.lcd<br>Method File: C:\LabSolutions\Data\Project1\LC Only Method 1.lcm<br>Report Format File: C:\LabSolutions\Data\Project1\Standard<br>Time: 10/24/2025 3:57:13 PM

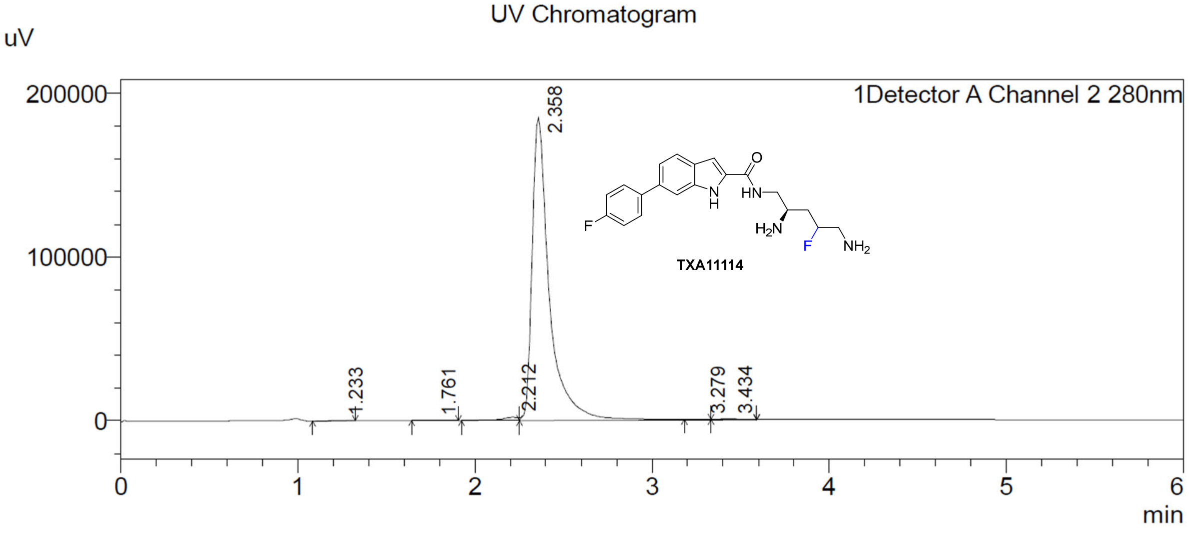

UV Peak Table
Detector A Channel 1 254nm

Peak#	Ret. Time	Area	Area%
1	0.983	26024	0.644
2	1.230	26208	0.649
3	1.595	4558	0.113
4	2.357	3984147	98.595
Total		4040938	100.000

UV Peak Table
Detector A Channel 2 280nm

Peak#	Ret. Time	Area	Area%
1	1.233	2472	0.193
2	1.761	897	0.070
3	2.212	15070	1.179
4	2.358	1255657	98.247
5	3.279	1582	0.124
6	3.434	2390	0.187
Total		1278067	100.000

HPLC Spectrum of TXA11164

10/27/2025 11:28:30 AM

==== Shimadzu LabSolutions Analysis Report ====

Acquired by	: System Administrato	Data File	: C:\LabSolutions\Data\AA\TXA11164.lcd
Sample ID	: TXA11164	Method File	: C:\LabSolutions\Data\Project1\LC Only Method 1.lcm
Tray#	: 1	Report Format File	: C:\LabSolutions\Data\Project1\Sta
Vial#	: 03		
Injection Volume	: 3		

UV Chromatogram

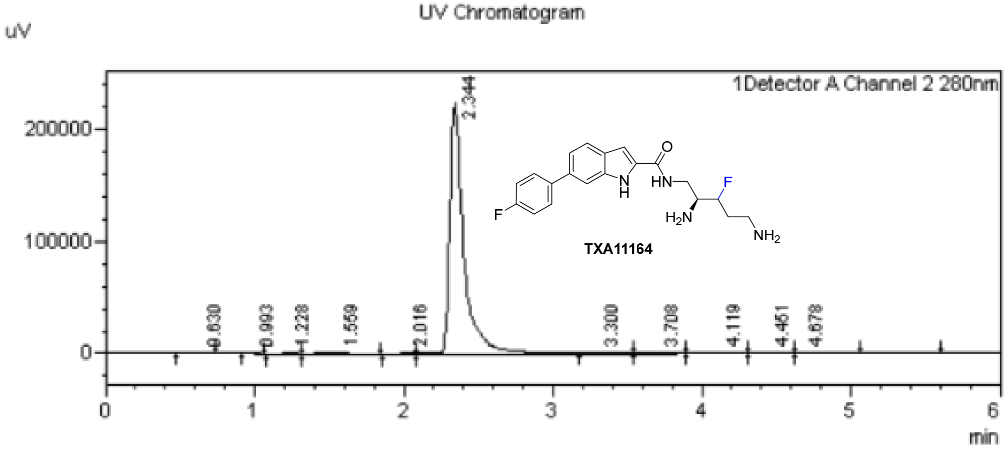

10/27/2025 11:28:30 AM

UV Peak Table
Detector A Channel 1 254nm

Peak#	Ret. Time	Area	Area%
1	0.625	1756	0.032
2	1.003	10521	0.193
3	1.223	19753	0.361
4	1.494	34408	0.630
5	2.023	16808	0.308
6	2.342	5063689	92.664
7	3.779	94220	1.724
8	4.135	62452	1.143
9	4.785	99230	1.816
10	4.961	20226	0.370
11	5.537	41529	0.760
Total		5464591	100.000

UV Peak Table
Detector A Channel 2 280nm

Peak#	Ret. Time	Area	Area%
1	0.630	1126	0.072
2	0.993	1953	0.125
3	1.228	2626	0.168
4	1.559	5723	0.365
5	2.016	2428	0.155
6	2.344	1516631	96.859
7	3.300	4846	0.310
8	3.708	5952	0.380
9	4.119	12918	0.825
10	4.451	8049	0.514
11	4.678	3560	0.227
Total		1565813	100.000

MASS Peak Table TIC(Event)

Event 1 = Pos
Event 2 = Neg

HPLC Spectrum of TXA12027

10/27/2025 11:32:44 A

==== Shimadzu LabSolutions Analysis Report ====

Acquired by	: System Administra	Data File	: C:\LabSolutions\Data\AA\TXA12027.lcd
Sample ID	: TXA12027	Method File	: C:\LabSolutions\Data\Project1\LC Only Method 1
Tray#	: 1	Report Format File	: C:\LabSolutions\Data\Project1\
Vial#	: 04		
Injection Volume	: 5		

UV Chromatogram

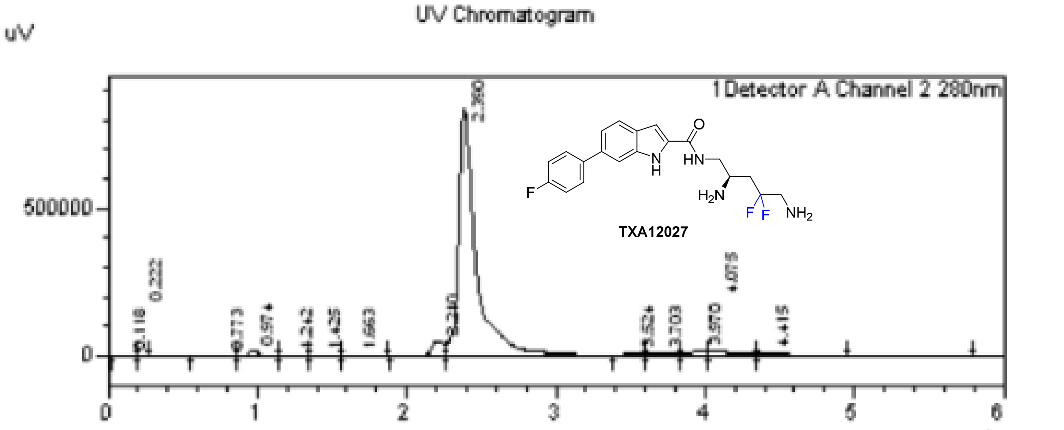

10/27/2025 11:32:44 AM

UV Peak Table
Detector A Channel 1 254nm

Peak#	Ret. Time	Area	Area%
1	0.109	196300	1.037
2	0.729	4563	0.024
3	0.974	90820	0.480
4	1.236	26382	0.139
5	1.450	12390	0.065
6	1.648	24953	0.132
7	2.390	#######	90.490
8	2.853	16106	0.085
9	3.527	33083	0.175
10	3.723	201105	1.063
11	3.972	281202	1.486
12	4.081	580206	3.066
13	4.416	304727	1.610
14	4.925	3169	0.017
15	5.087	2586	0.014
16	5.534	15157	0.080
17	5.675	6858	0.036
Total		#######	100.000

UV Peak Table
Detector A Channel 2 280nm

Peak#	Ret. Time	Area	Area%
1	0.118	3417	0.042
2	0.222	1537	0.019
3	0.773	10590	0.131
4	0.974	93856	1.159
5	1.242	2974	0.037
6	1.425	2328	0.029
7	1.663	3545	0.044
8	2.210	278928	3.444
9	2.390	#######	88.842
10	3.524	35651	0.440
11	3.703	72114	0.890
12	3.970	110124	1.360
13	4.075	192126	2.372
14	4.415	96453	1.191
Total		#######	100.000

MASS Peak Table TIC(Event)

Event 1 = Pos
Event 2 = Neg

^{1}H-NMR Spectrum of TXA11114 in MeOH-d4, 300 MHz NMR

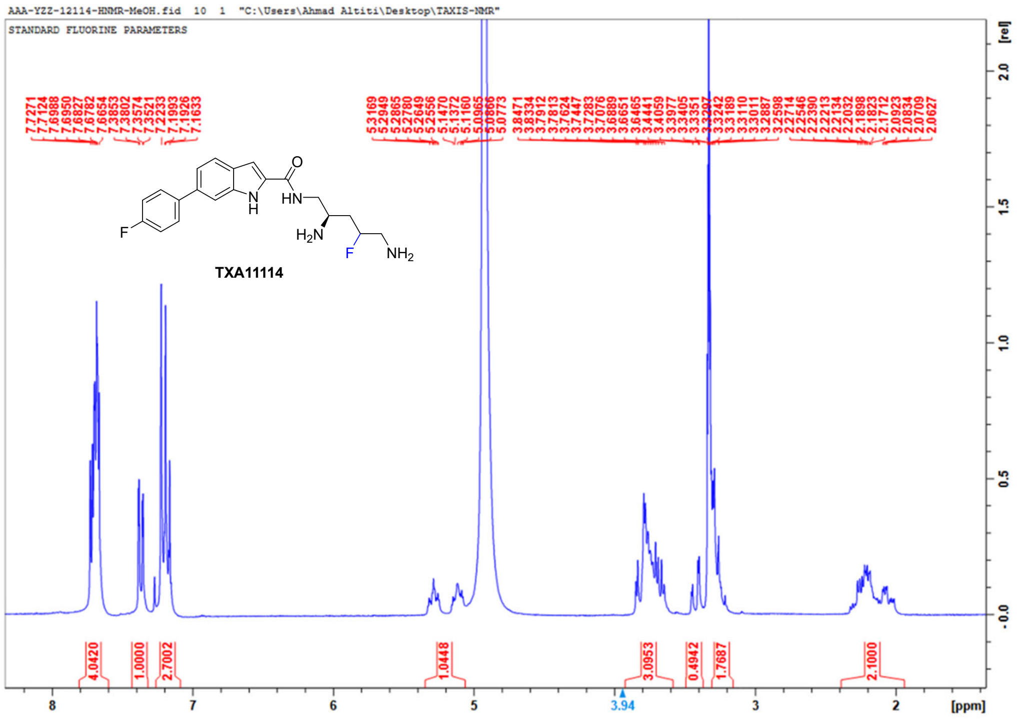

^{13}C-NMR Spectrum of TXA11114 in MeOH-d4, 75 MHz NMR

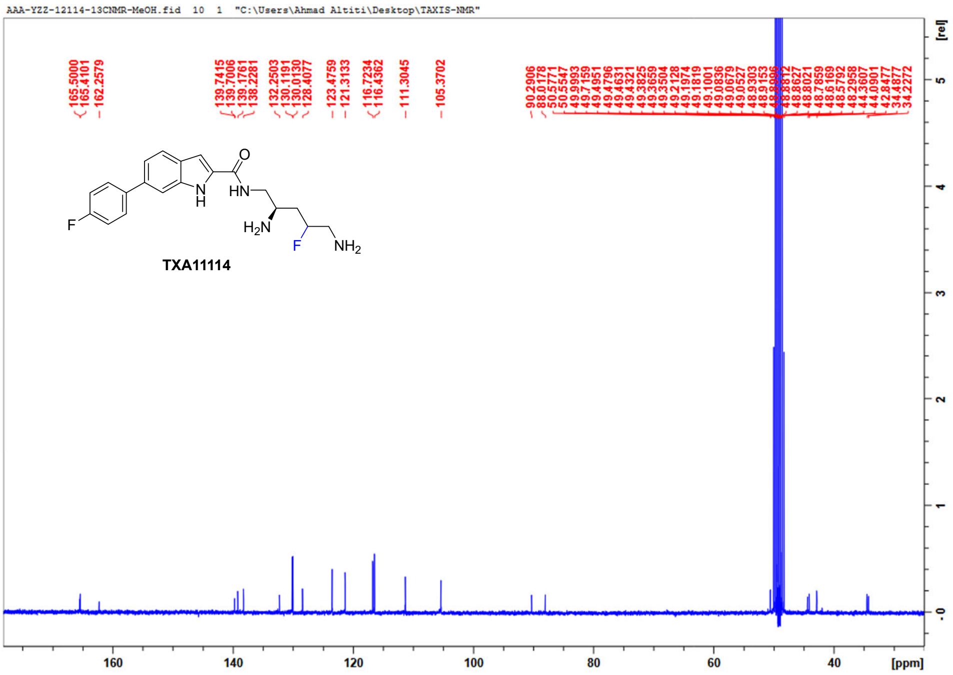

^{1}H-NMR Spectrum of TXA11164 in MeOH-d4, 300 MHz NMR

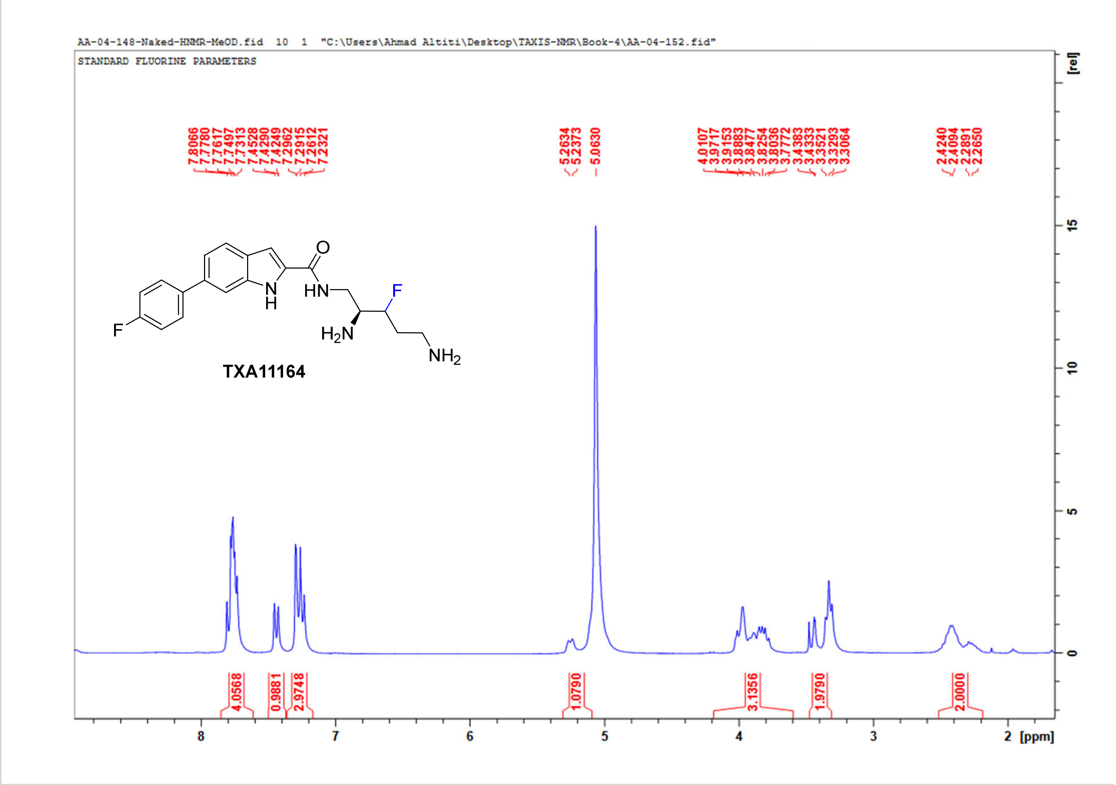

^{13}C-NMR Spectrum of TXA11164 in MeOH-d4, 75 MHz NMR

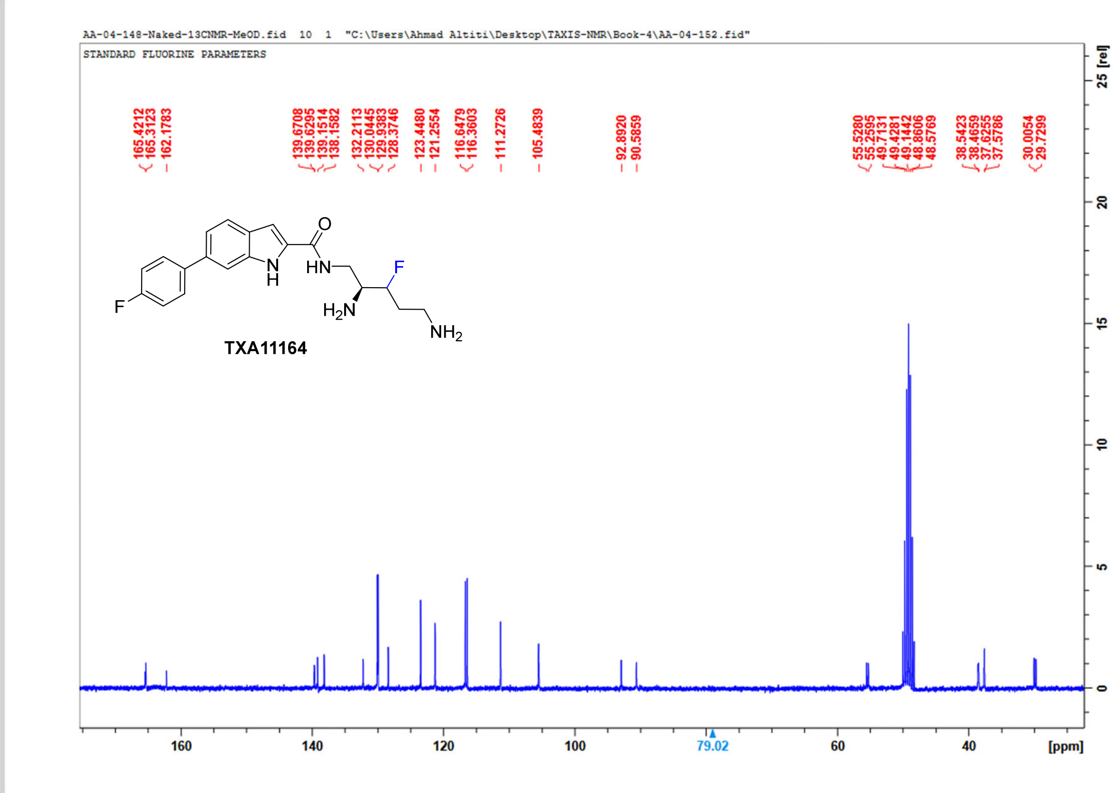

^{19}F-NMR Spectrum of TXA11164 in MeOH-d4, 282 MHz NMR

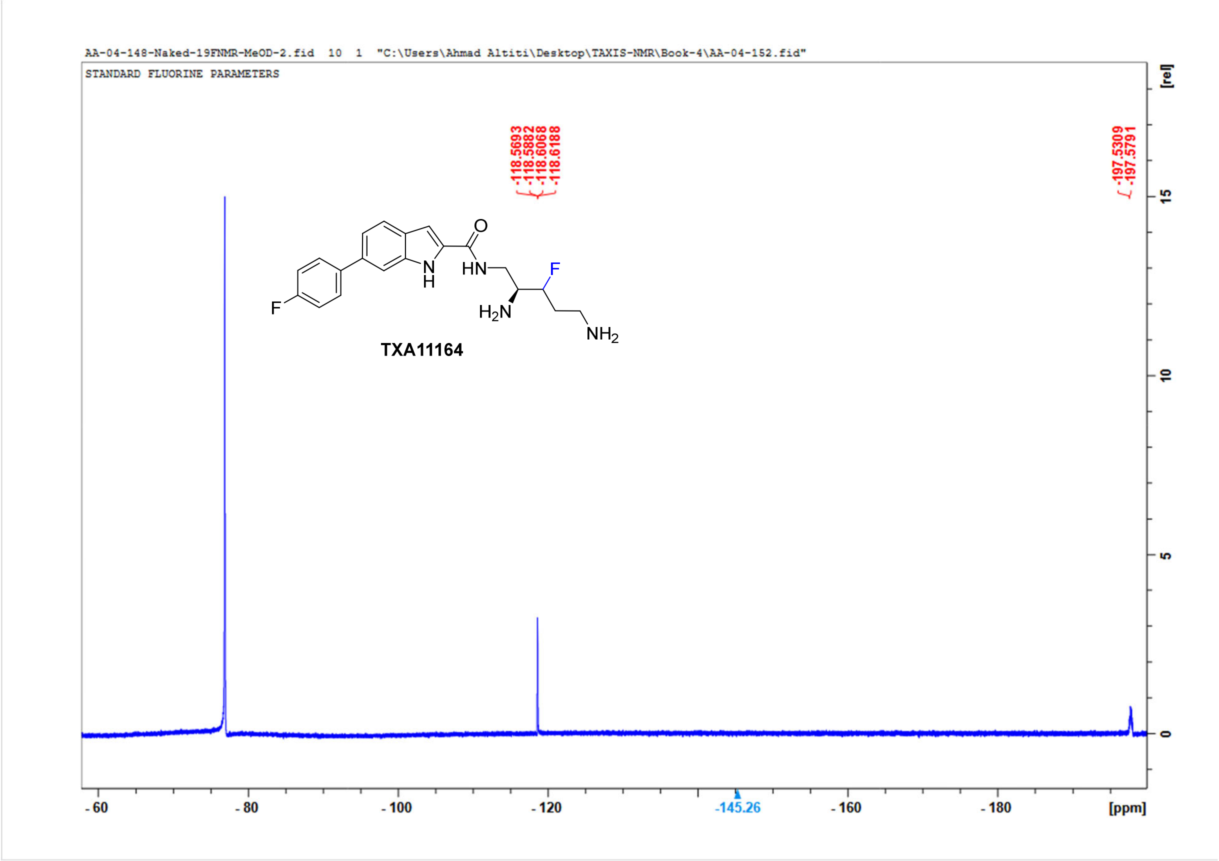

^{1}H-NMR Spectrum of TXA12027 in MeOH-d4, 300 MHz NMR

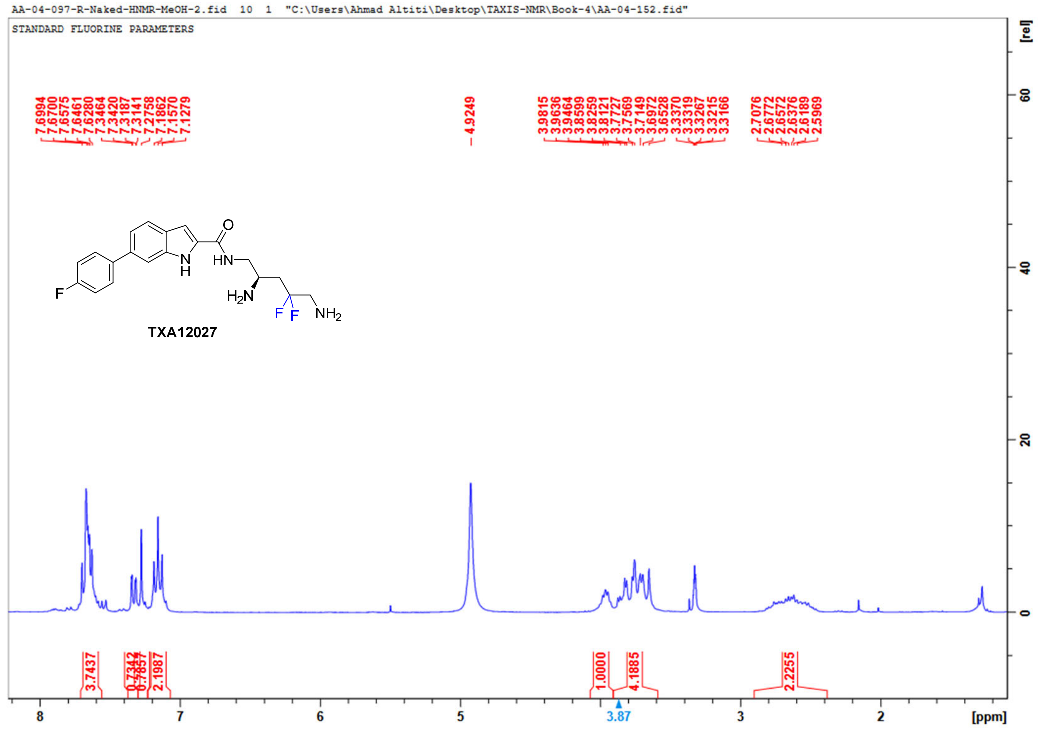

^{13}C-NMR Spectrum of TXA12027 in MeOH-d4, 75 MHz NMR

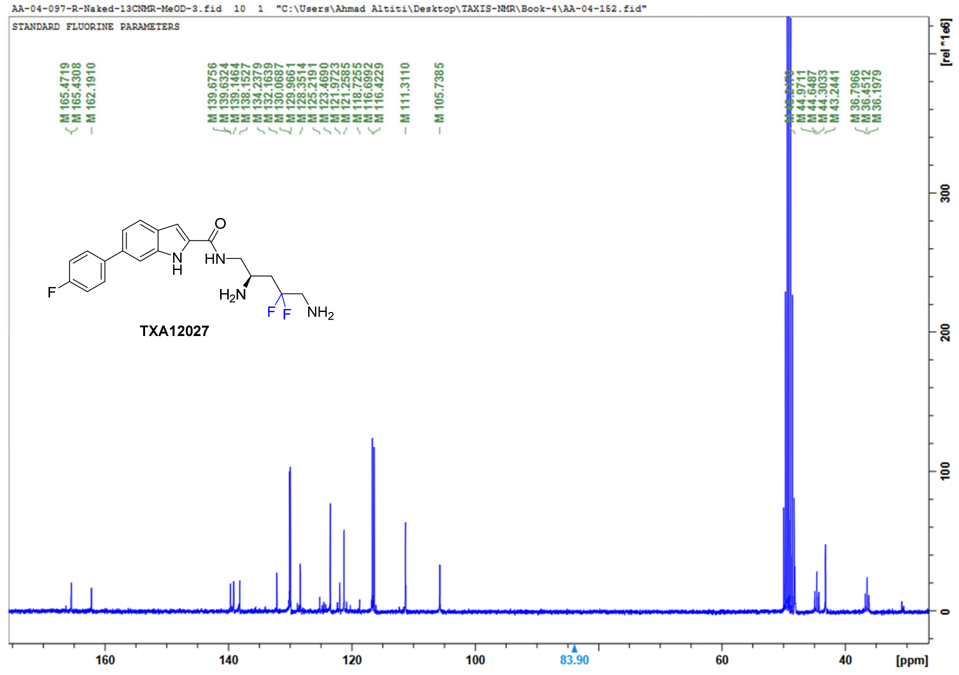

^{19}F-NMR Spectrum of TXA12027 in MeOH-d4, 282 MHz NMR

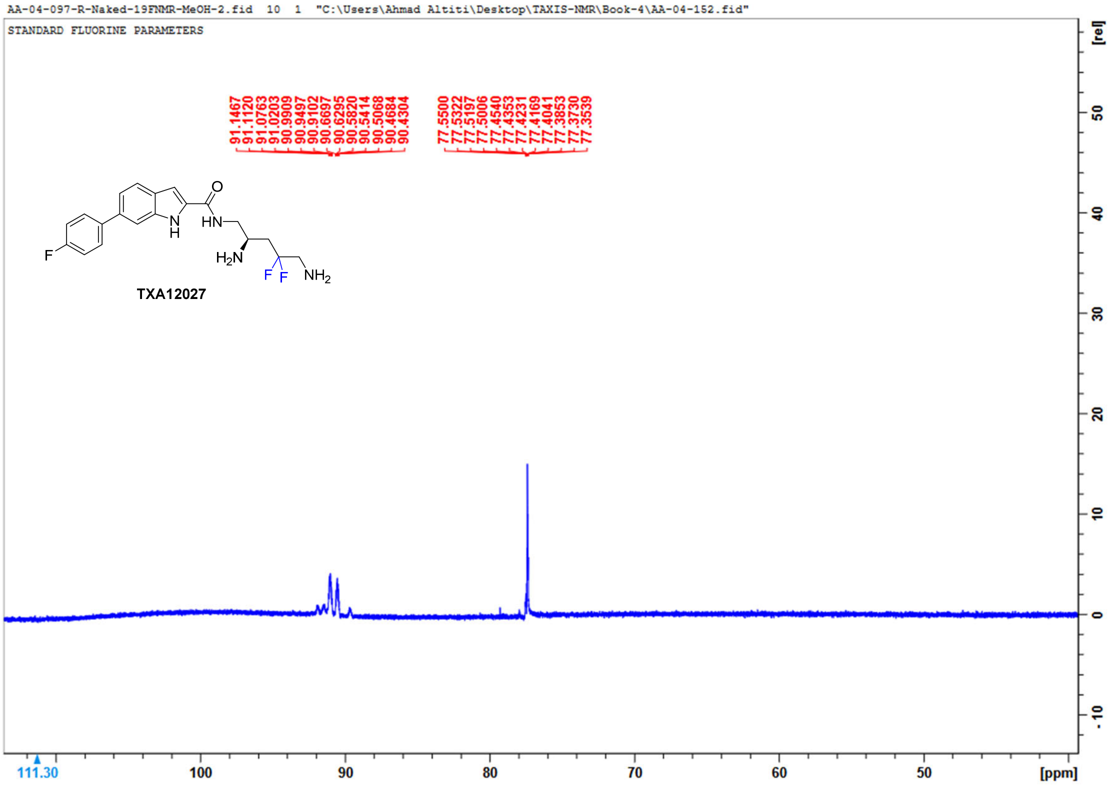

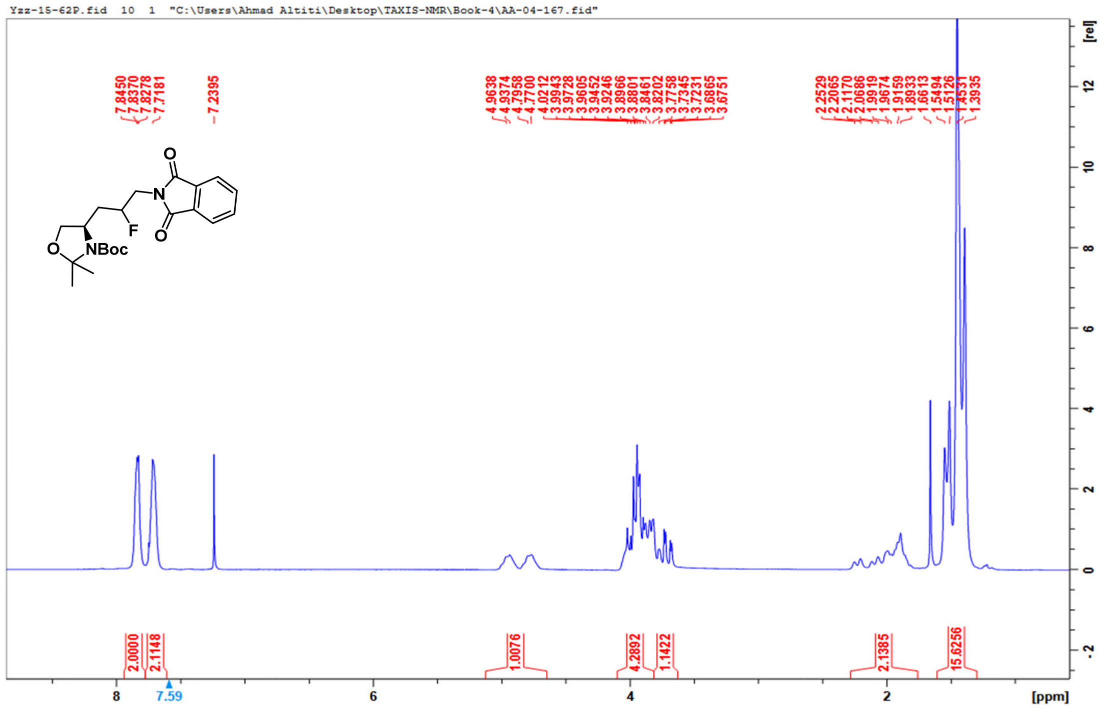

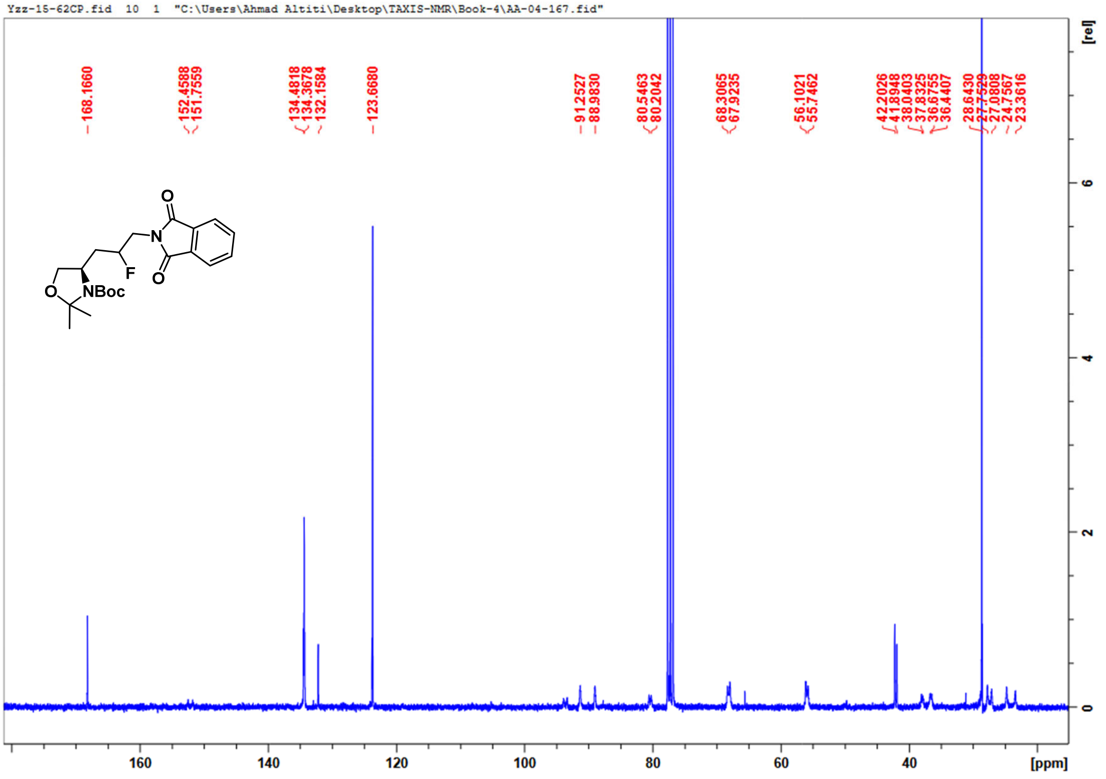

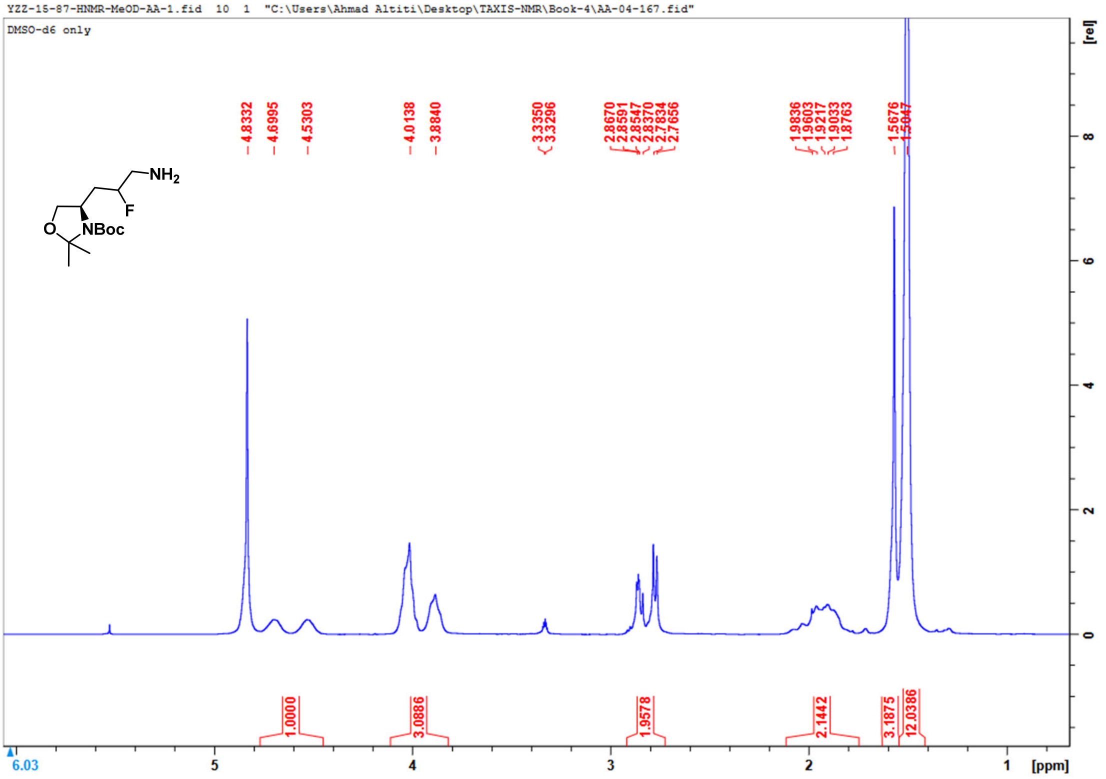
YZZ-15-87-HNMR-MeOD-AA-1.fid 10 1 "C:\Users\Ahmad Altiti\Desktop\TAXIS-NMR\Book-4\AA-04-167.fid"
DMSO-d6 only
NH2
F
O
NBoc
[rel]
[ppm]

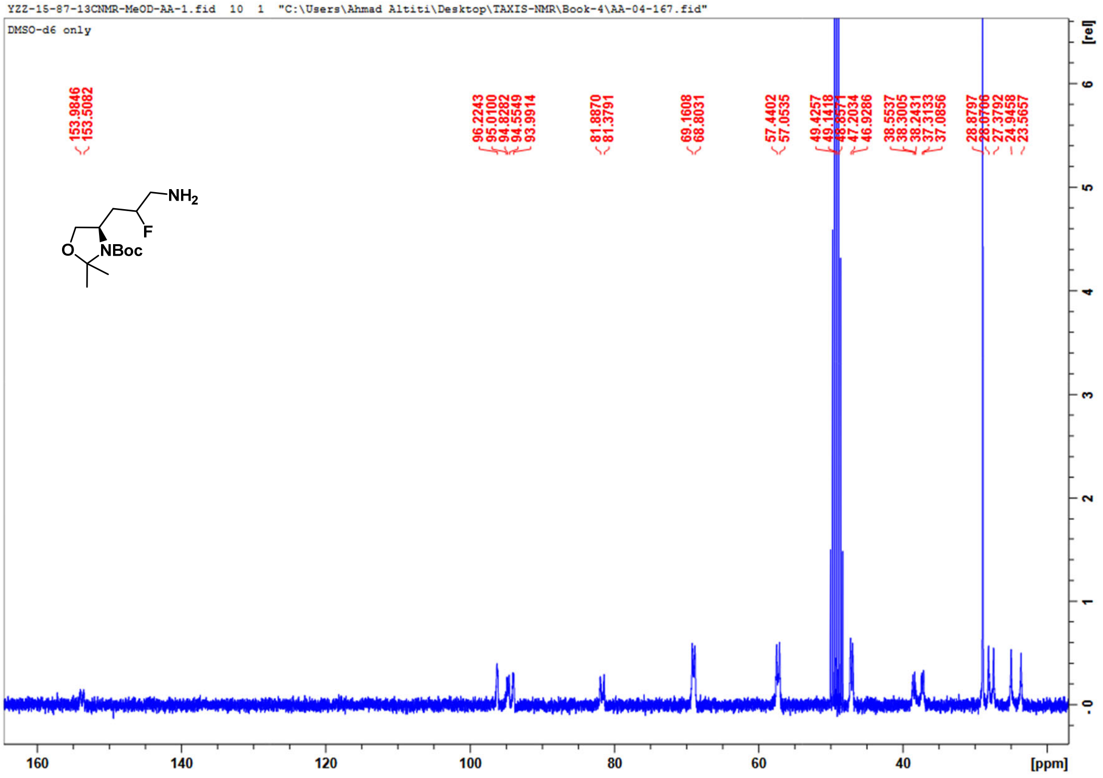
YZZ-15-87-13CNMR-MeOD-AA-1.fid 10 1 "C:\Users\Ahmad Altiti\Desktop\TAXIS-NMR\Book-4\AA-04-167.fid"
DMSO-d6 only
NH2
F
O
NBoc
[rel]
[ppm]

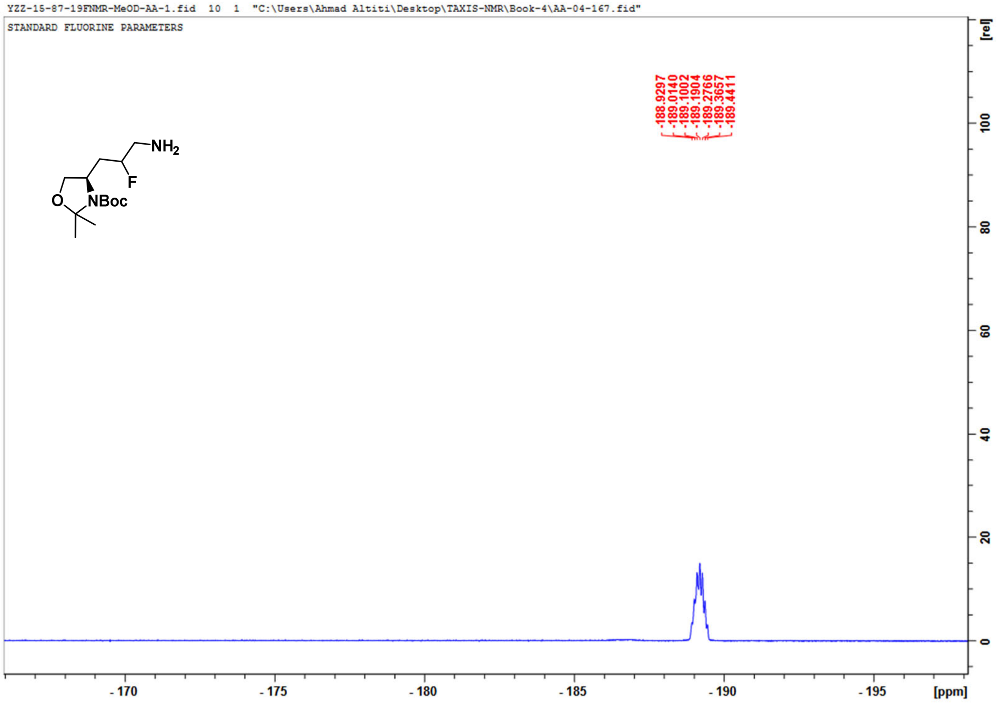
YZZ-15-87-19FNMR-MeOD-AA-1.fid 10 1 "C:\Users\Ahmad Altiti\Desktop\TAXIS-NMR\Book-4\AA-04-167.fid"
STANDARD FLUORINE PARAMETERS
NH2
F
O
NBoc
-188.9297
-189.0140
-189.1002
-189.1904
-189.2766
-189.3657
-189.4411
[rel]
[ppm]

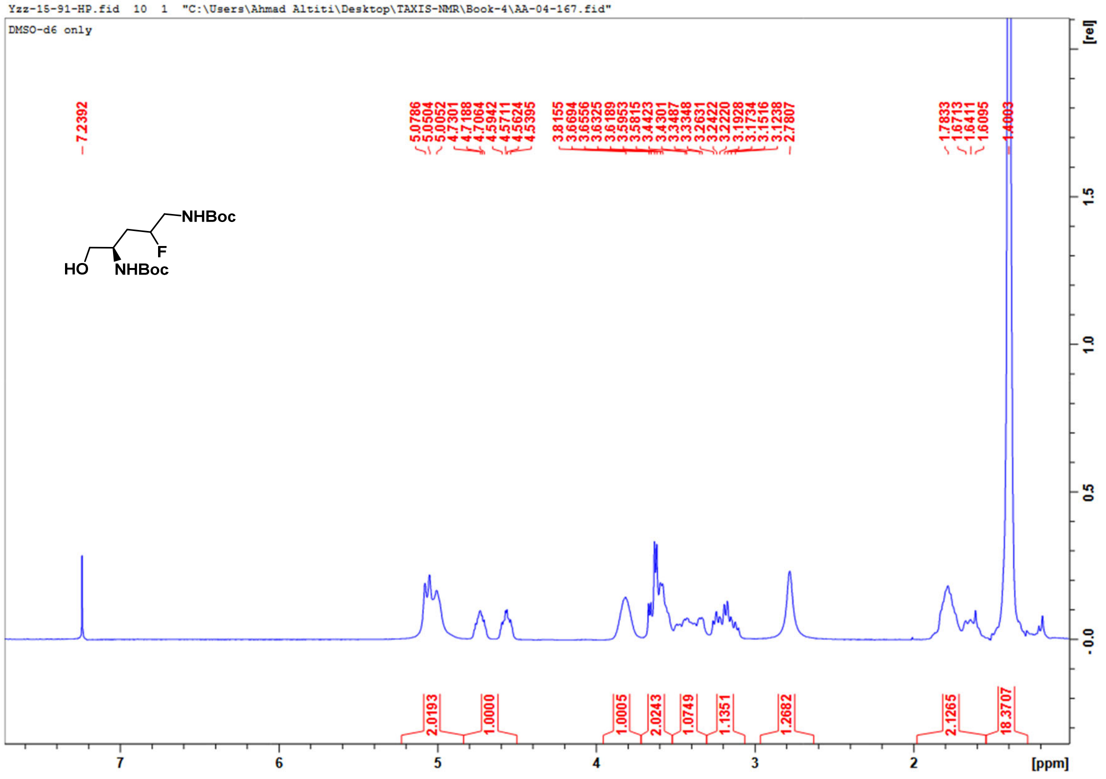
Yzz-15-91-HP.fid 10 1 "C:\Users\Ahmad Altiti\Desktop\TAXIS-NMR\Book-4\AA-04-167.fid"
DMSO-d6 only
NHBoc
F
HO
NHBoc
7.2392
5.0786
5.0504
5.0052
4.7301
4.7188
4.7064
4.5942
4.5711
4.5624
4.5395
3.8155
3.6694
3.6556
3.6325
3.6189
3.5953
3.5815
3.4423
3.4301
3.3487
3.3348
3.2631
3.2422
3.2220
3.1928
3.1734
3.1516
3.1238
2.7807
1.7833
1.6713
1.6411
1.6095
1.4003
2.0193
1.0000
1.0005
2.0243
1.0749
1.1351
1.2682
2.1265
18.3707
[rel]
[ppm]

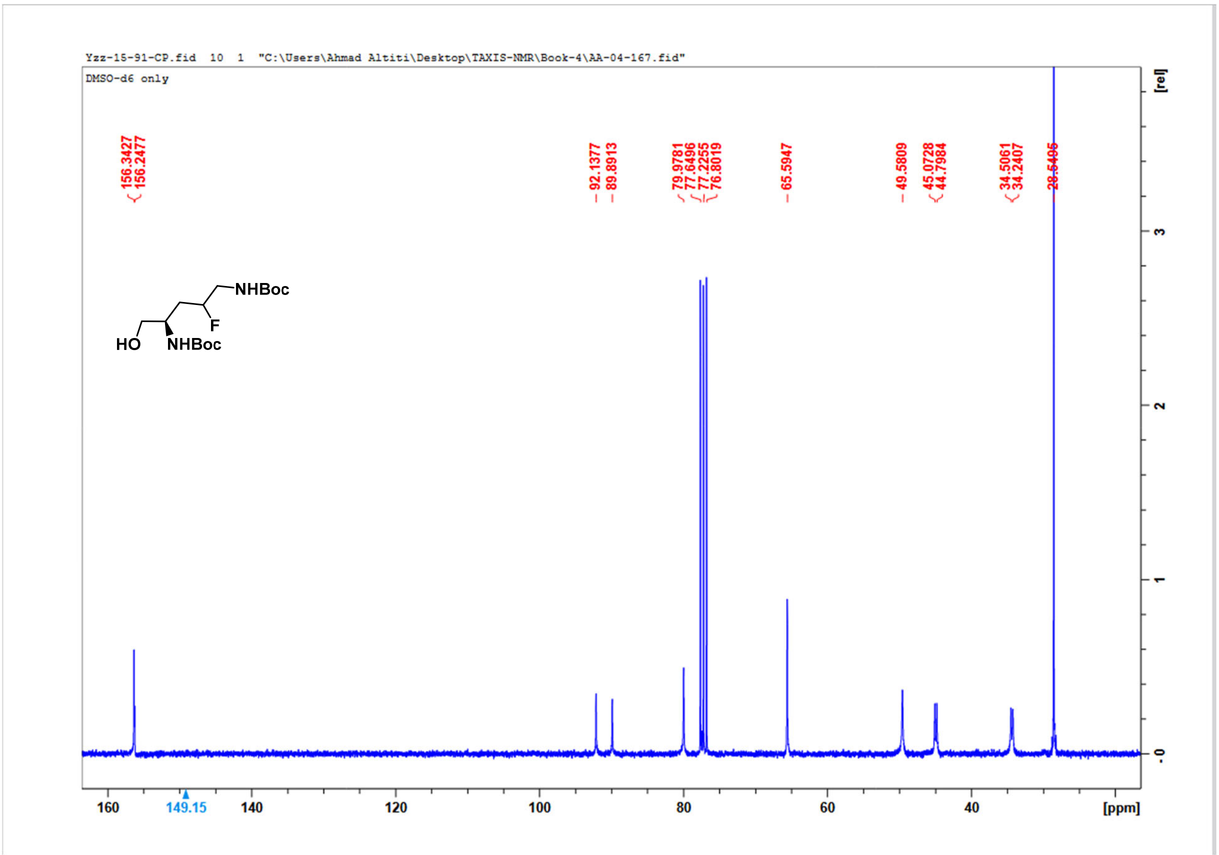

Yzz-15-91-CP.fid 10 1 "C:\Users\Ahmad Altiti\Desktop\TAXIS-NMR\Book-4\AA-04-167.fid"
DMSO-d6 only
156.3427
156.2477
92.1377
89.8913
79.9781
77.6496
77.2255
76.8019
65.5947
49.5809
45.0728
44.7984
34.5061
34.2407
28.5495
NHBoc
F
HO
NHBoc
[rel]
149.15
[ppm]

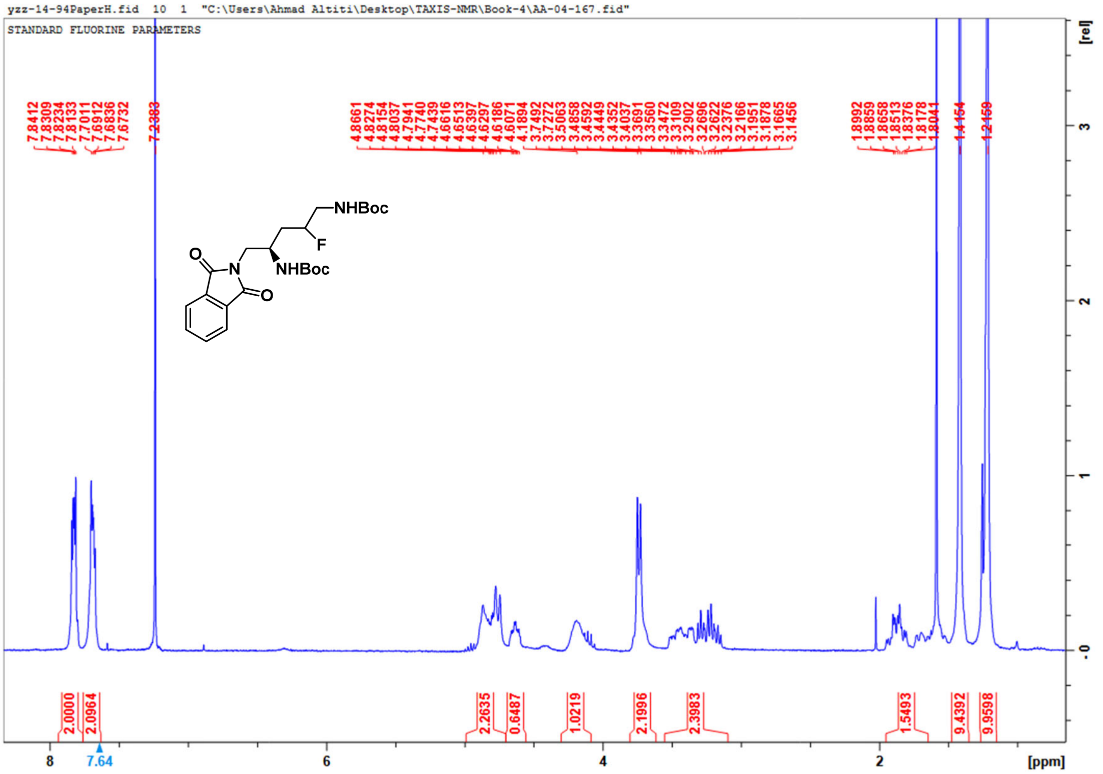

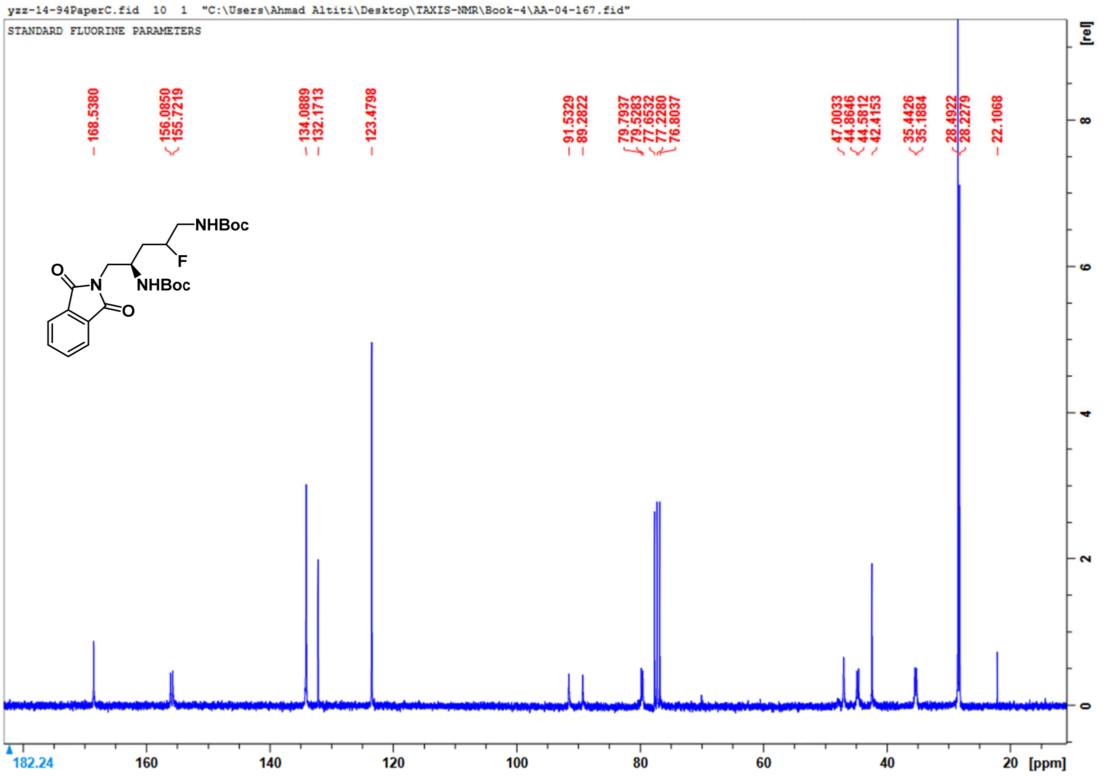

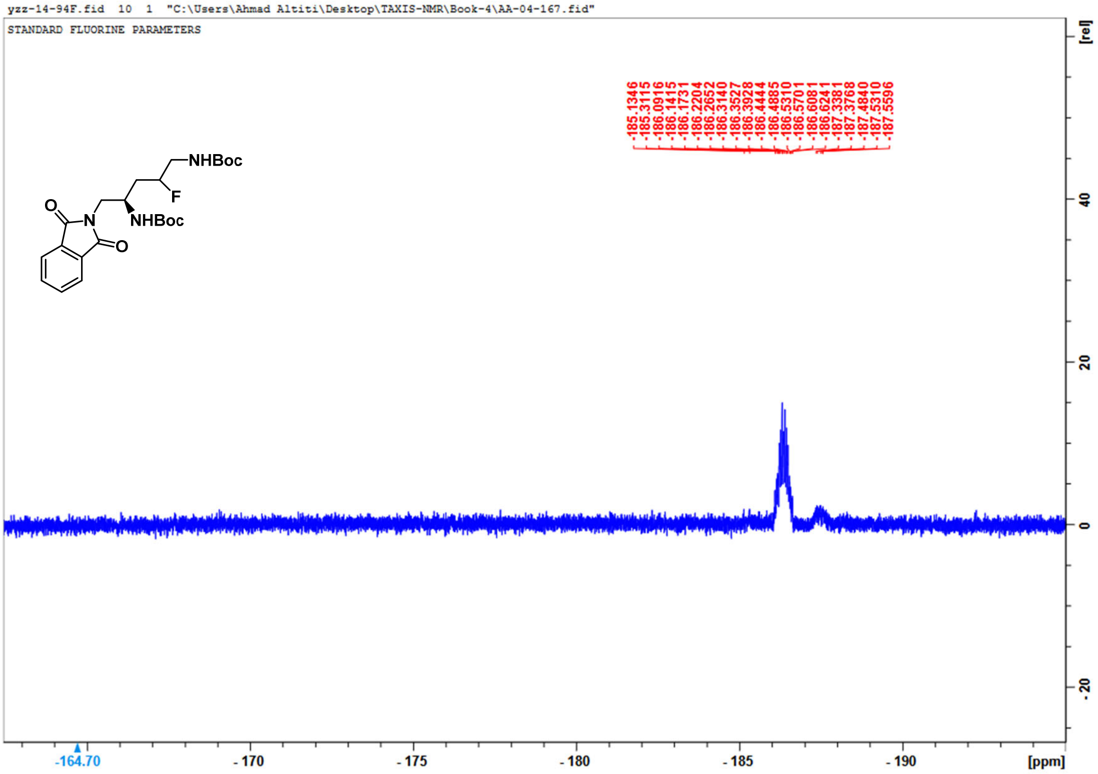

yzz-14-94F.fid 10 1 "C:\Users\Ahmad Altiti\Desktop\TAXIS-NMR\Book-4\AA-04-167.fid"
STANDARD FLUORINE PARAMETERS
NHBoc
F
NHBoc
O
N
O
-185.1346
-185.3115
-186.0916
-186.1415
-186.1731
-186.2204
-186.2652
-186.3140
-186.3527
-186.3928
-186.4444
-186.4885
-186.5310
-186.5701
-186.6081
-186.6241
-187.3381
-187.3768
-187.4840
-187.5310
-187.5596
[rel]
40
20
0
-20
-164.70
-170
-175
-180
-185
-190
[ppm]

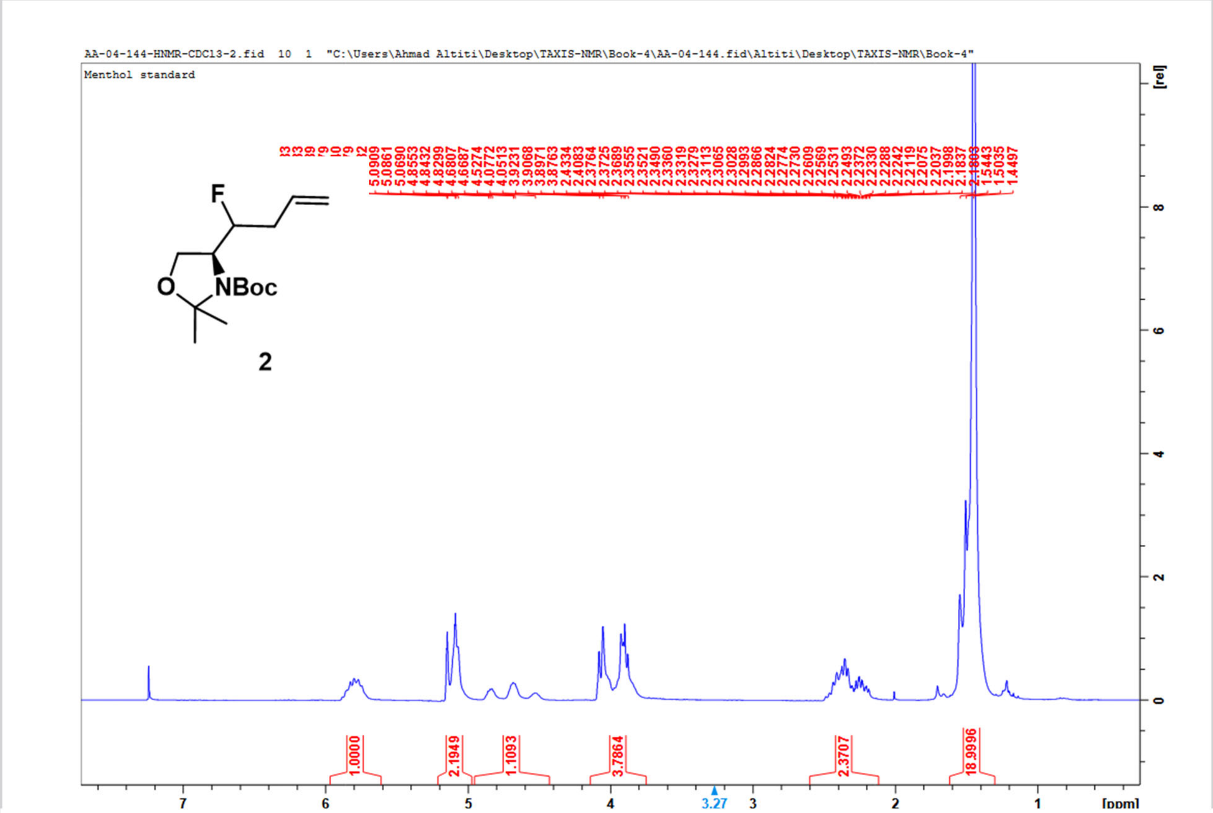
AA-04-144-HNMR-CDCl3-2.fid 10 1 "C:\Users\Ahmad Altiti\Desktop\TAXIS-NMR\Book-4\AA-04-144.fid\Altiti\Desktop\TAXIS-NMR\Book-4"
Menthol standard
F
O
NBoc
2
5.0909 5.0861 5.0690 4.8553 4.8432 4.8299 4.6807 4.6687 4.5274 4.0772 4.0513 3.9231 3.9068 3.8971 3.8763 2.4334 2.4083 2.3764 2.3725 2.3689 2.3555 2.3521 2.3490 2.3360 2.3319 2.3279 2.3113 2.3065 2.3028 2.2993 2.2886 2.2824 2.2774 2.2730 2.2609 2.2569 2.2531 2.2493 2.2372 2.2330 2.2288 2.2242 2.2119 2.2075 2.2037 2.1998 2.1837 1.5443 1.5035 1.4497
1.0000
2.1949
1.1093
3.7864
2.3707
18.9996
3.27
[ppm]
[rel]

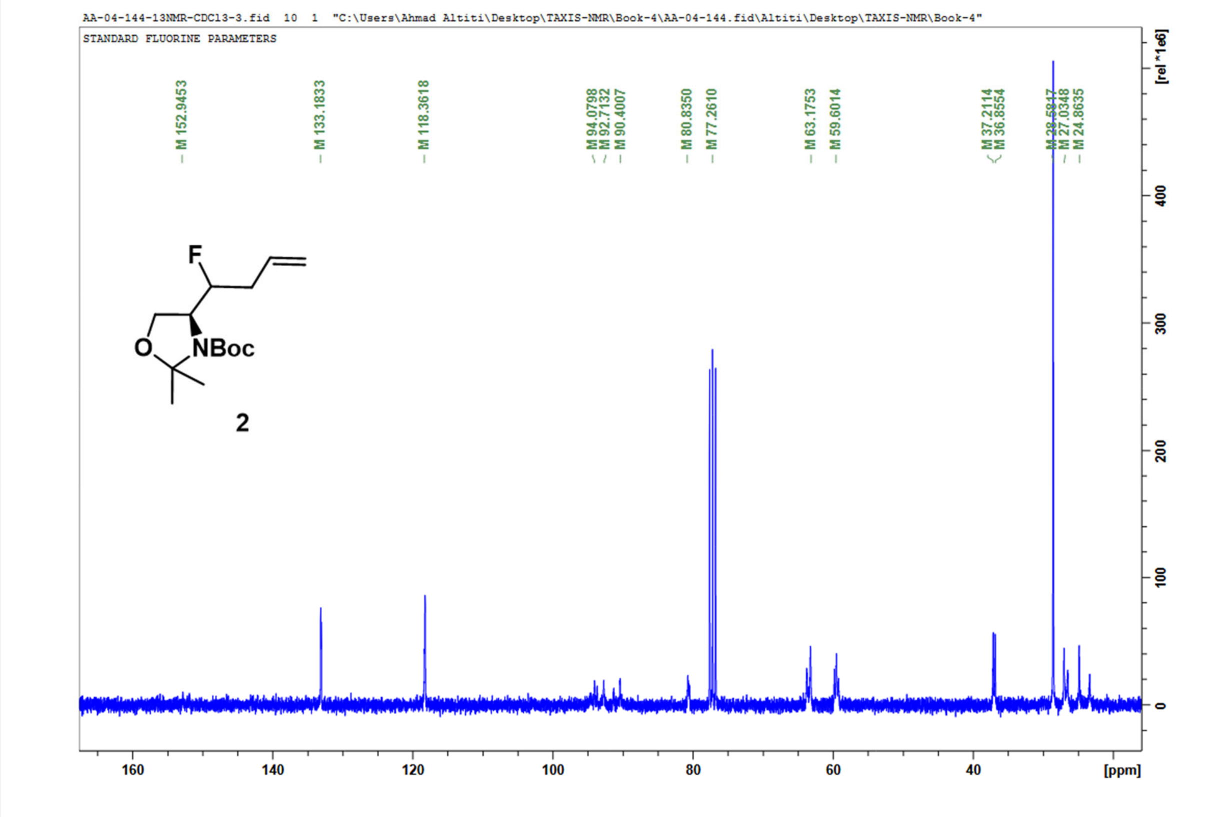
AA-04-144-13NMR-CDCl3-3.fid 10 1 "C:\Users\Ahmad Altiti\Desktop\TAXIS-NMR\Book-4\AA-04-144.fid\Altiti\Desktop\TAXIS-NMR\Book-4"
STANDARD FLUORINE PARAMETERS
M 152.9453
M 133.1833
M 118.3618
M 94.0798
M 92.7132
M 90.4007
M 80.8350
M 77.2610
M 63.1753
M 59.6014
M 37.2114
M 36.8554
M 27.0348
M 24.8635
F
O
NBoc
2
[ppm]
[rel*1e6]

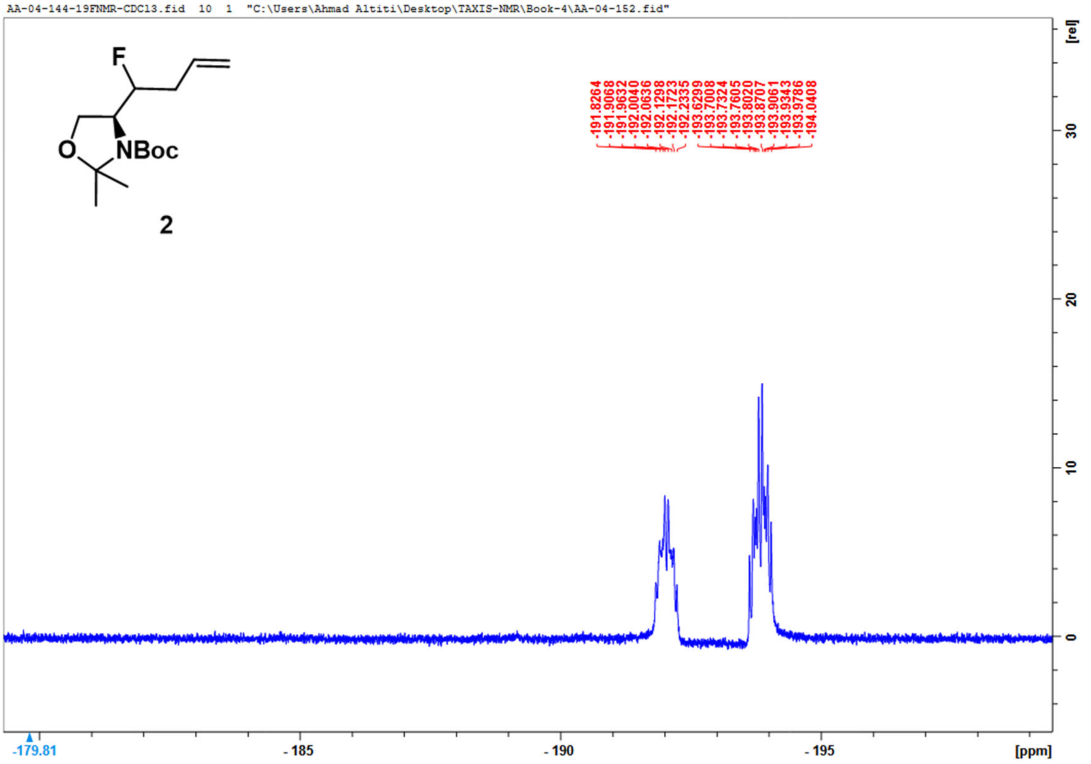
AA-04-144-19FNMR-CDCl3.fid 10 1 "C:\Users\Ahmad Altiti\Desktop\TAXIS-NMR\Book-4\AA-04-152.fid"
F
NBoc
O
2
-191.8264
-191.9068
-191.9632
-192.0040
-192.0636
-192.1298
-192.1723
-192.2335
-193.6299
-193.7008
-193.7324
-193.7605
-193.8020
-193.8707
-193.9061
-193.9343
-193.9786
-194.0408
-179.81
-185
-190
-195
[ppm]
[rel]

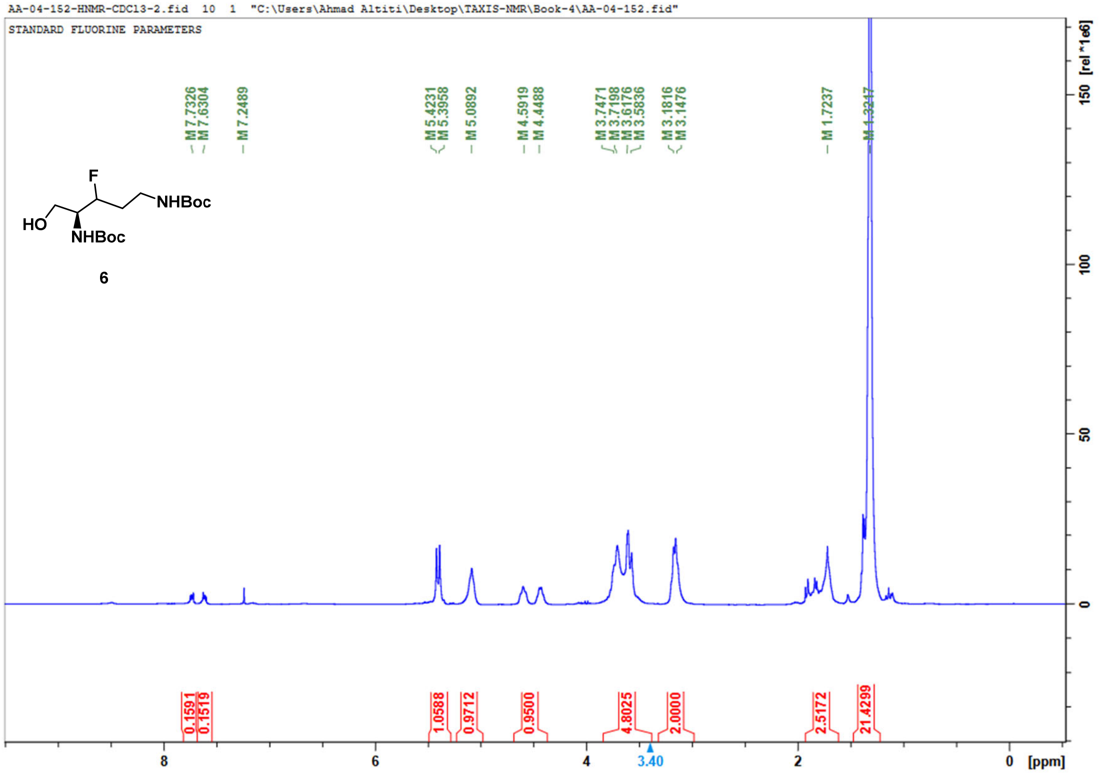
AA-04-152-HNMR-CDCl3-2.fid 10 1 "C:\Users\Ahmad Altiti\Desktop\TAXIS-NMR\Book-4\AA-04-152.fid"
STANDARD FLUORINE PARAMETERS
M 7.7326
M 7.6304
M 7.2489
M 5.4231
M 5.3958
M 5.0892
M 4.5919
M 4.4488
M 3.7471
M 3.7198
M 3.6176
M 3.5836
M 3.1816
M 3.1476
M 1.7237
F
HO
NHBoc
NHBoc
6
0.1591
0.1519
1.0588
0.9712
0.9500
4.8025
2.0000
2.5172
21.4299
3.40
[ppm]
[rel*1e6]

AA-04-152-13CNMR-CDCl3-1.fid 10 1 "C:\Users\Ahmad Altiti\Desktop\TAXIS-NMR\Book-4\AA-04-152.fid"

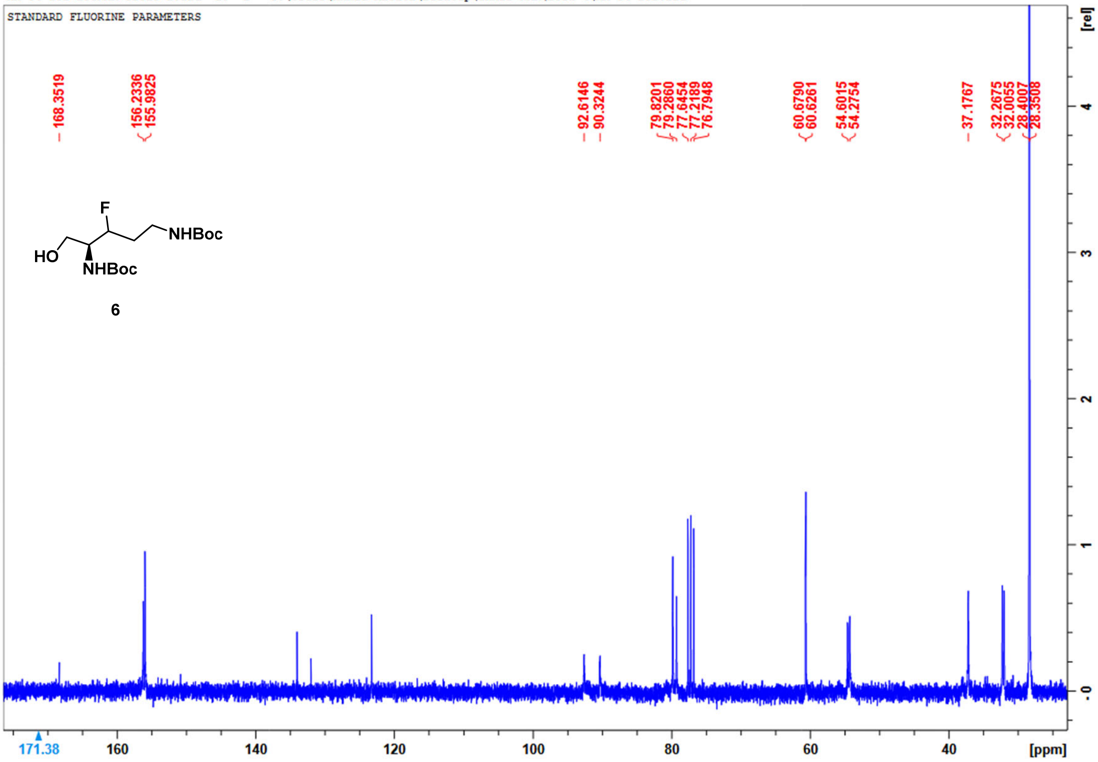

AA-04-152-19FNMR-CDCl3-1.fid 10 1 "C:\Users\Ahmad Altiti\Desktop\TAXIS-NMR\Book-4\AA-04-152.fid"

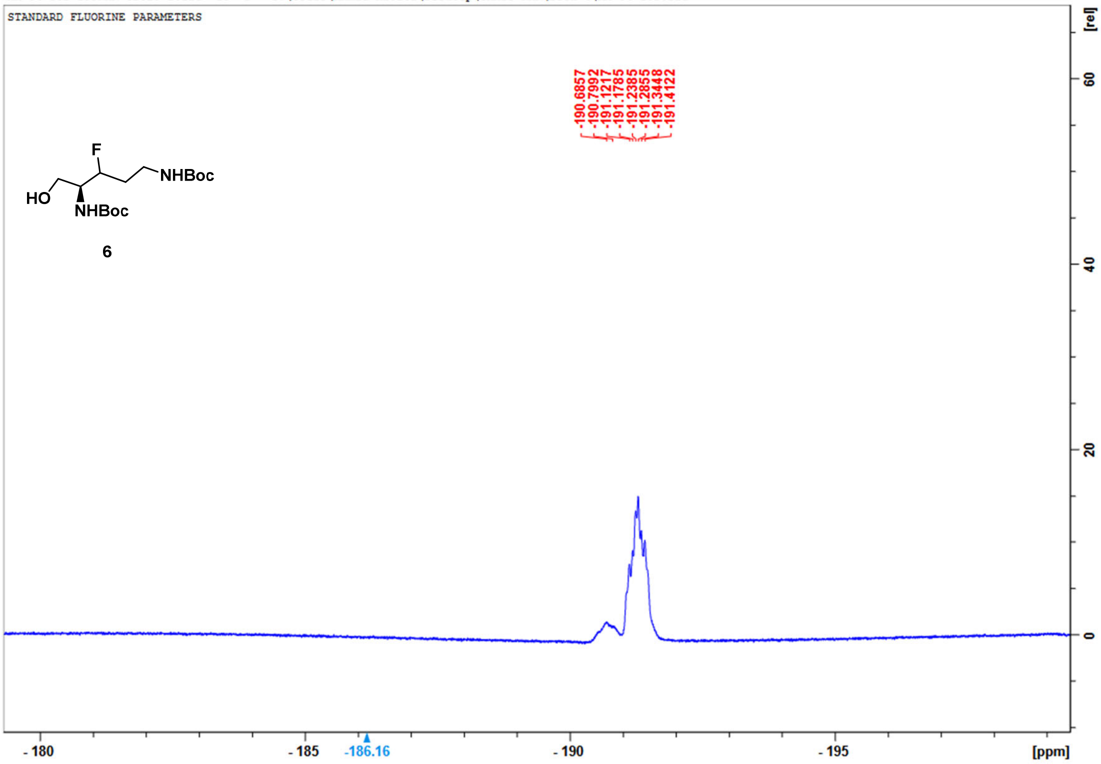

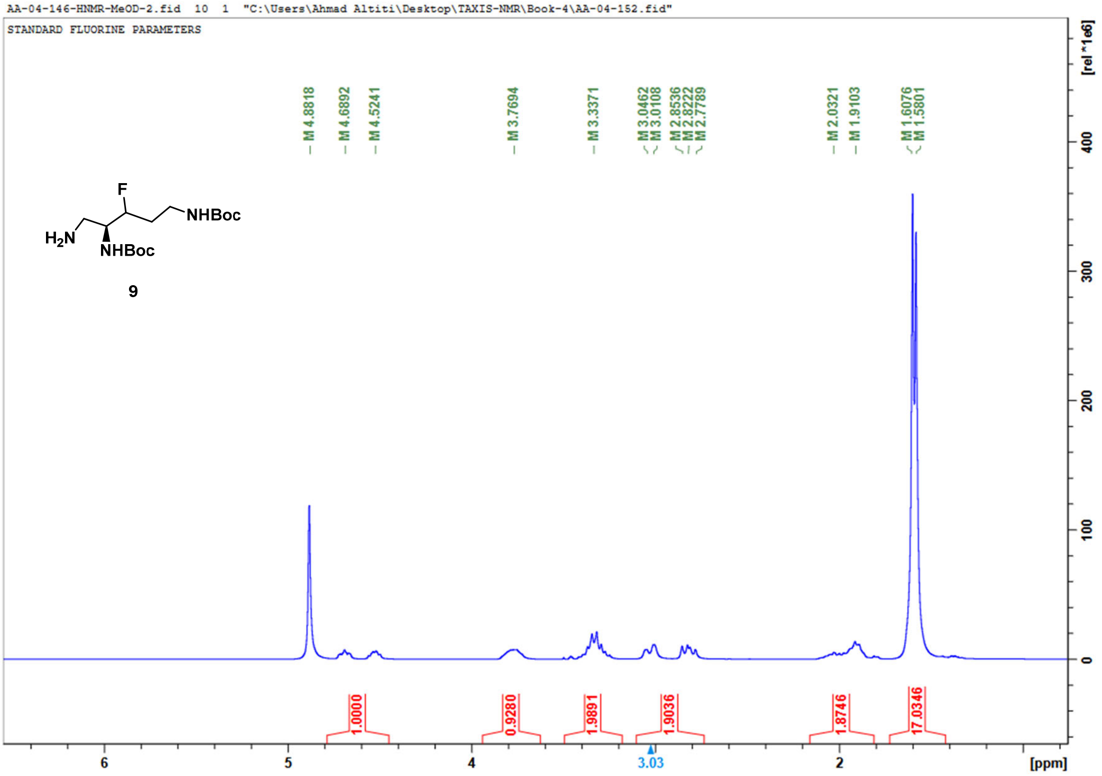
AA-04-146-HNMR-MeOD-2.fid 10 1 "C:\Users\Ahmad Altiti\Desktop\TAXIS-NMR\Book-4\AA-04-152.fid"
STANDARD FLUORINE PARAMETERS
M 4.8818
M 4.6892
M 4.5241
M 3.7694
M 3.3371
M 3.0462
M 3.0108
M 2.8536
M 2.8222
M 2.7789
M 2.0321
M 1.9103
M 1.6076
M 1.5801
F
NHBoc
H2N
NHBoc
9
1.0000
0.9280
1.9891
1.9036
1.8746
17.0346
3.03
[ppm]
[rel*1e6]

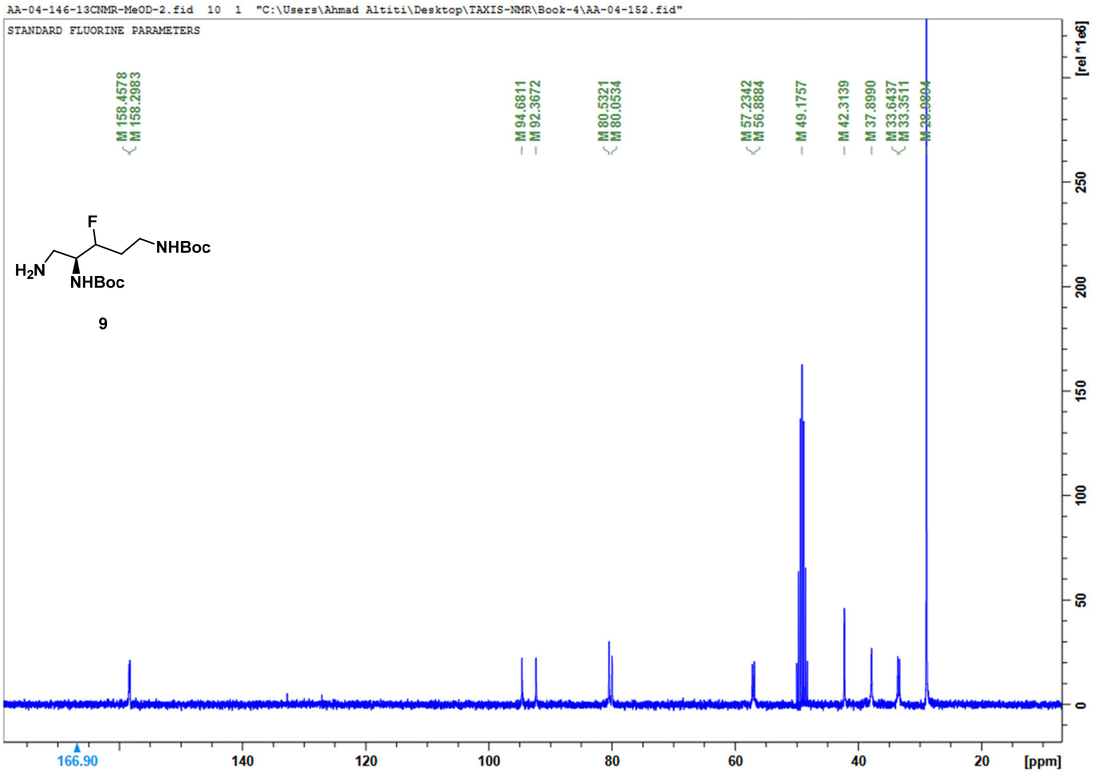
AA-04-146-13CNMR-MeOD-2.fid 10 1 "C:\Users\Ahmad Altiti\Desktop\TAXIS-NMR\Book-4\AA-04-152.fid"
STANDARD FLUORINE PARAMETERS
M 158.4578
M 158.2983
M 94.6811
M 92.3672
M 80.5321
M 80.0534
M 57.2342
M 56.8884
M 49.1757
M 42.3139
M 37.8990
M 33.6437
M 33.3511
F
NHBoc
H2N
NHBoc
9
166.90
[ppm]
[rel*1e6]

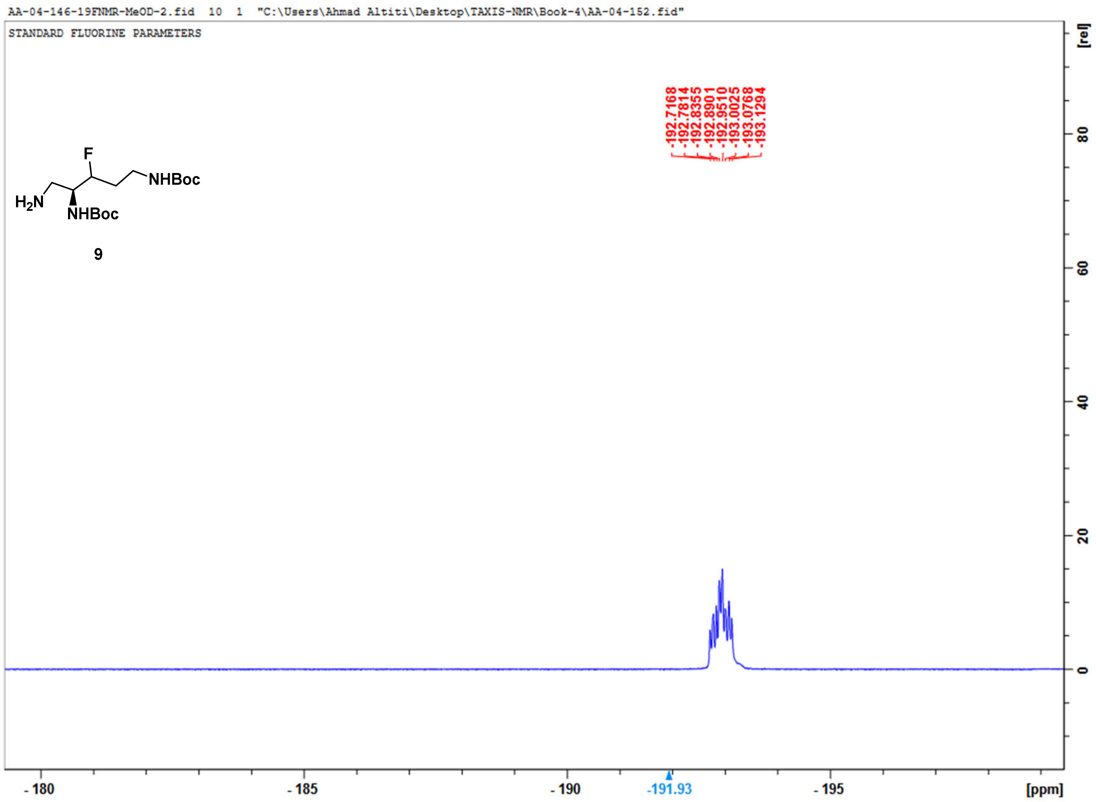
AA-04-146-19FNMR-MeOD-2.fid 10 1 "C:\Users\Ahmad Altiti\Desktop\TAXIS-NMR\Book-4\AA-04-152.fid"
STANDARD FLUORINE PARAMETERS
F
NHBoc
H2N
NHBoc
9
-192.7168
-192.7814
-192.8355
-192.8901
-192.9510
-193.0025
-193.0768
-193.1294
[rel]
80
60
40
20
0
- 180
- 185
- 190
-191.93
- 195
[ppm]

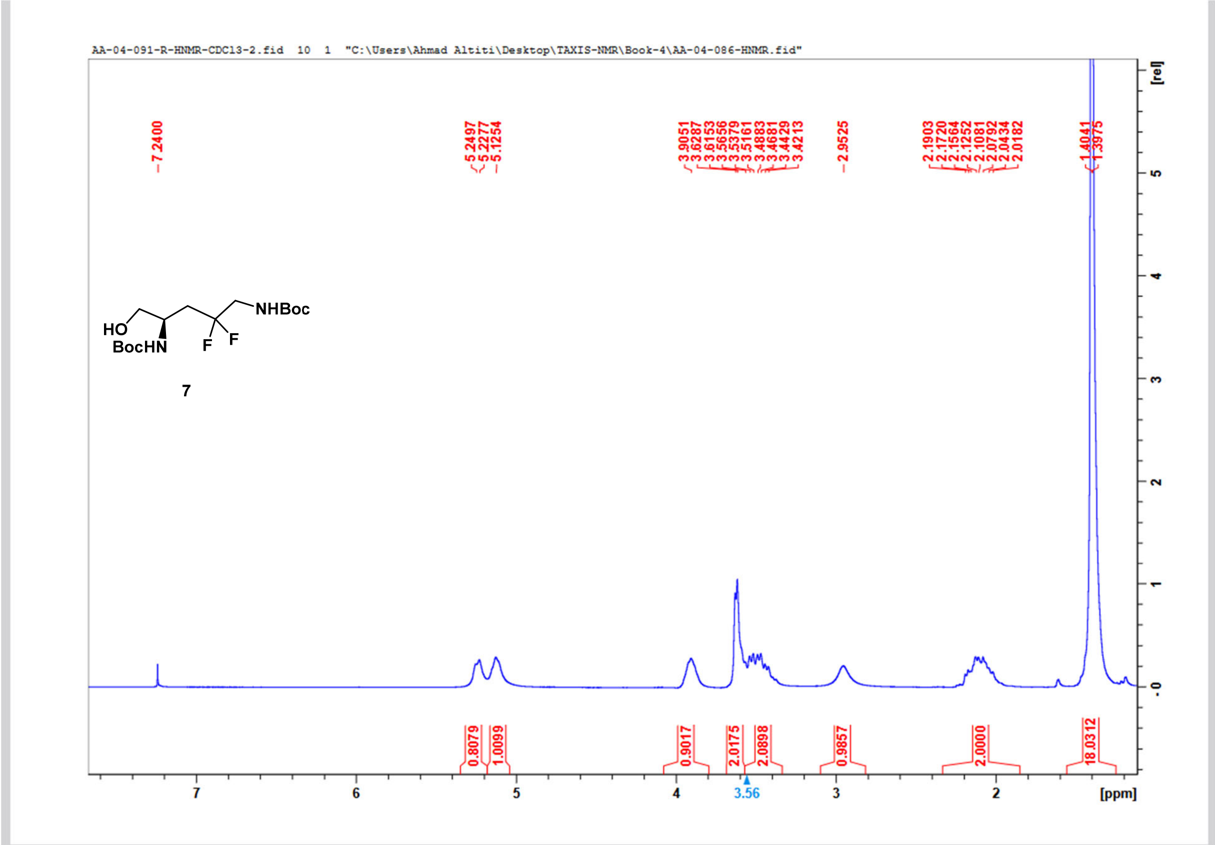
AA-04-091-R-HNMR-CDCl3-2.fid 10 1 "C:\Users\Ahmad Altiti\Desktop\TAXIS-NMR\Book-4\AA-04-086-HNMR.fid"
NHBoc
HO
BocHN
F F
7
[ppm]
[rel]

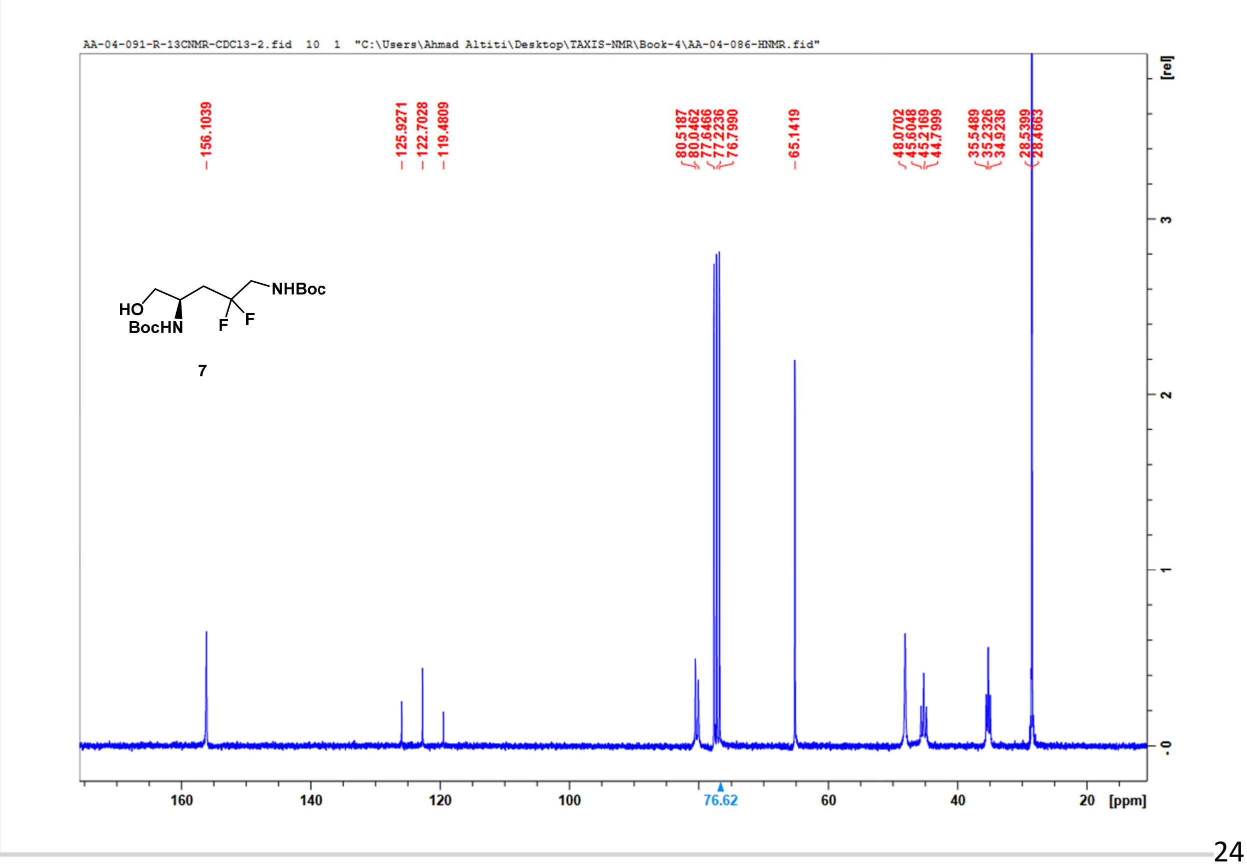
AA-04-091-R-13CNMR-CDCl3-2.fid 10 1 "C:\Users\Ahmad Altiti\Desktop\TAXIS-NMR\Book-4\AA-04-086-HNMR.fid"
NHBoc
HO
BocHN
F F
7
[ppm]
[rel]

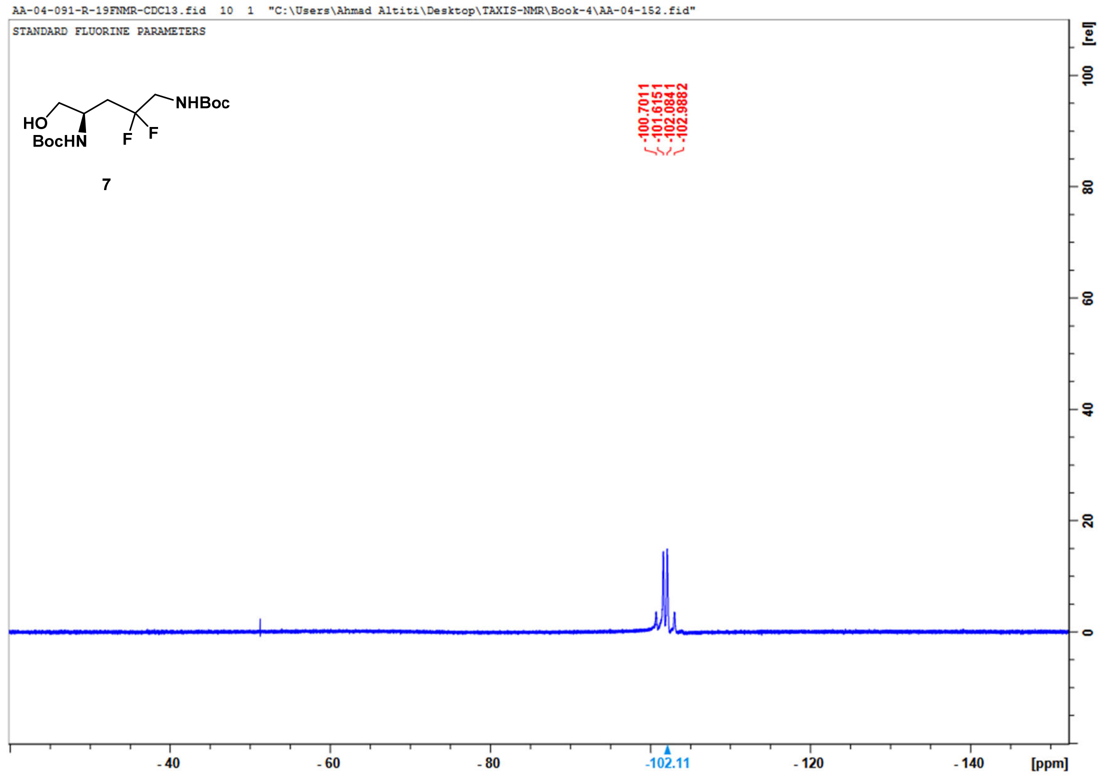
AA-04-091-R-19FNMR-CDCl3.fid 10 1 "C:\Users\Ahmad Altiti\Desktop\TAXIS-NMR\Book-4\AA-04-152.fid"
STANDARD FLUORINE PARAMETERS
HO
BocHN
NHBoc
F F
7
-100.7011
-101.6151
-102.0841
-102.9882
-102.11
[ppm]
[rel]

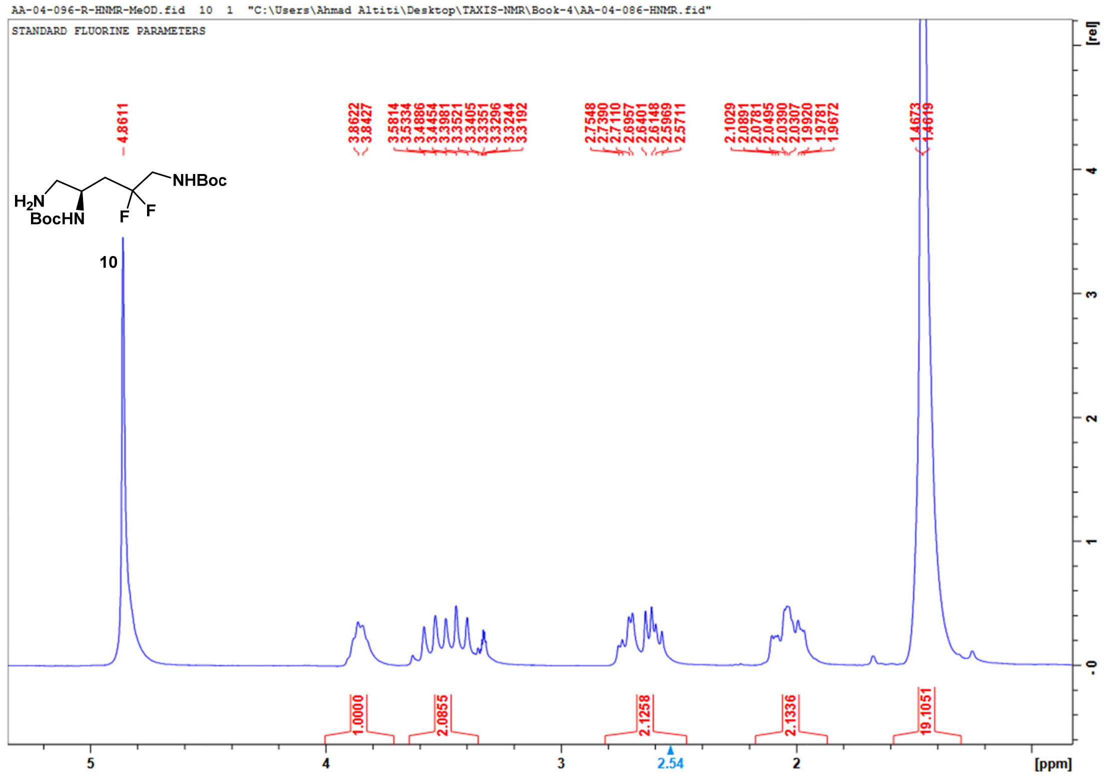
AA-04-096-R-HNMR-MeOD.fid 10 1 "C:\Users\Ahmad Altiti\Desktop\TAXIS-NMR\Book-4\AA-04-086-HNMR.fid"
STANDARD FLUORINE PARAMETERS
H2N
BocHN
NHBoc
F F
10
4.8611
1.0000
2.0855
2.1258
2.1336
19.1051
2.54
[ppm]
[rel]

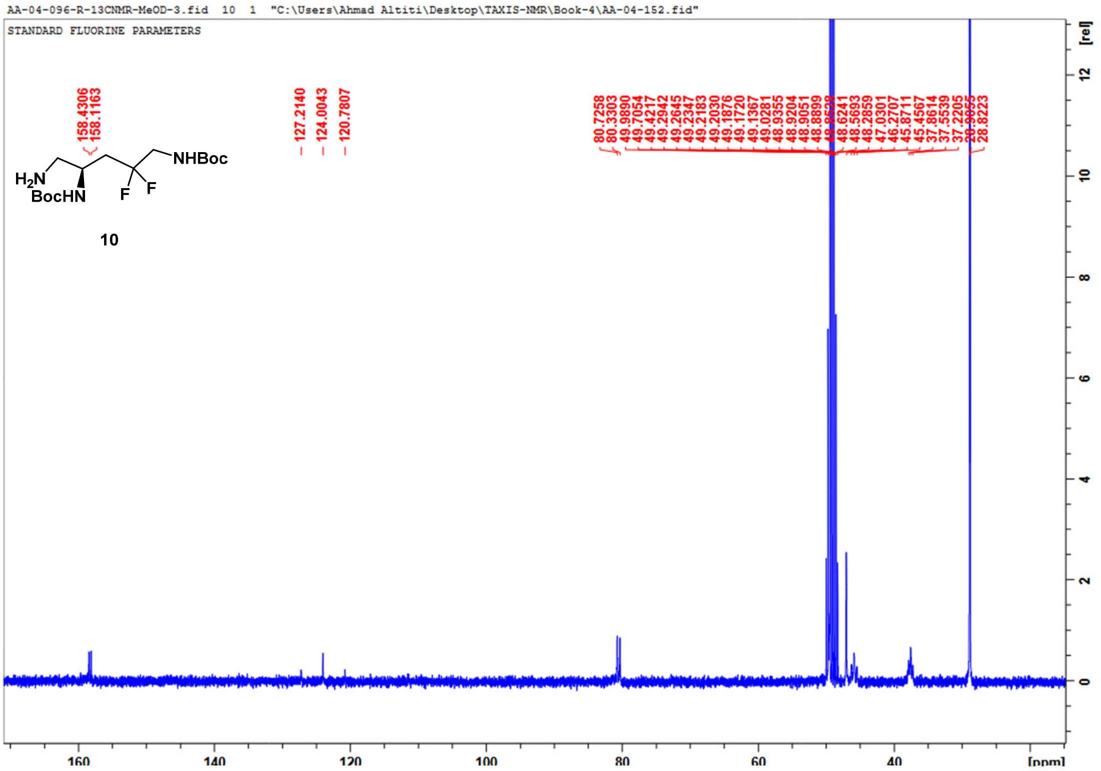

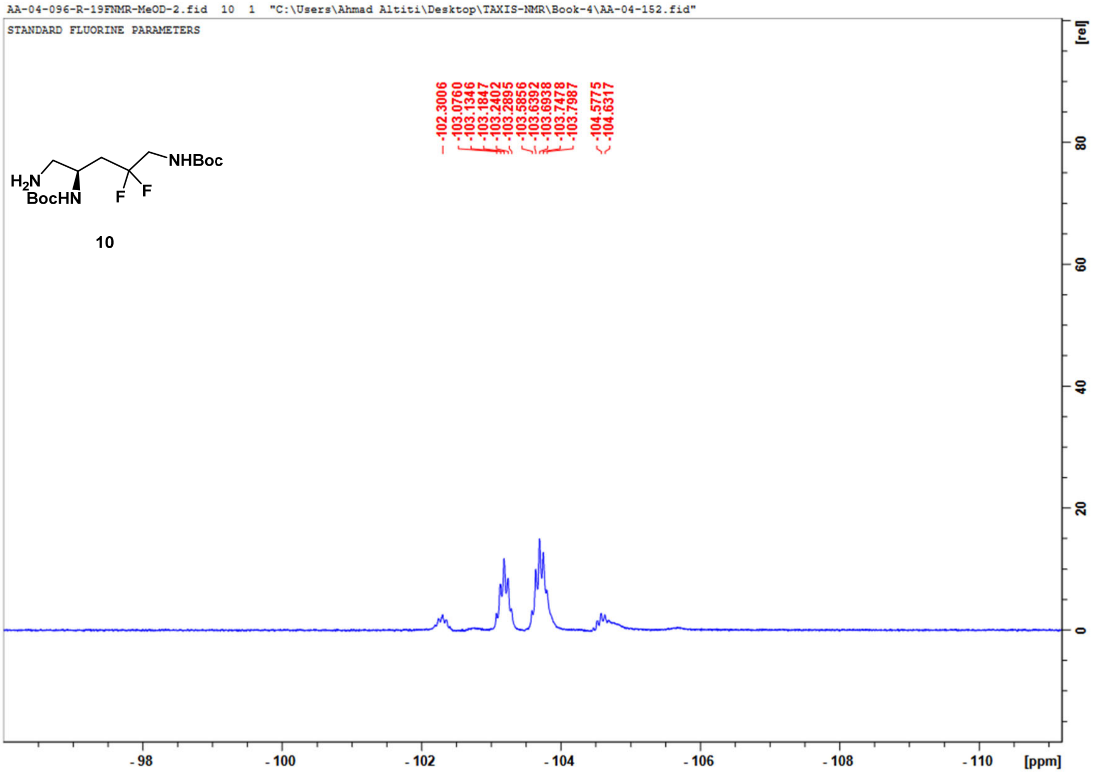

Yzz-15-62P.fid 10 1 "C:\Users\Ahmad Altiti\Desktop\TAXIS-NMR\Book-4\AA-04-167.fid"

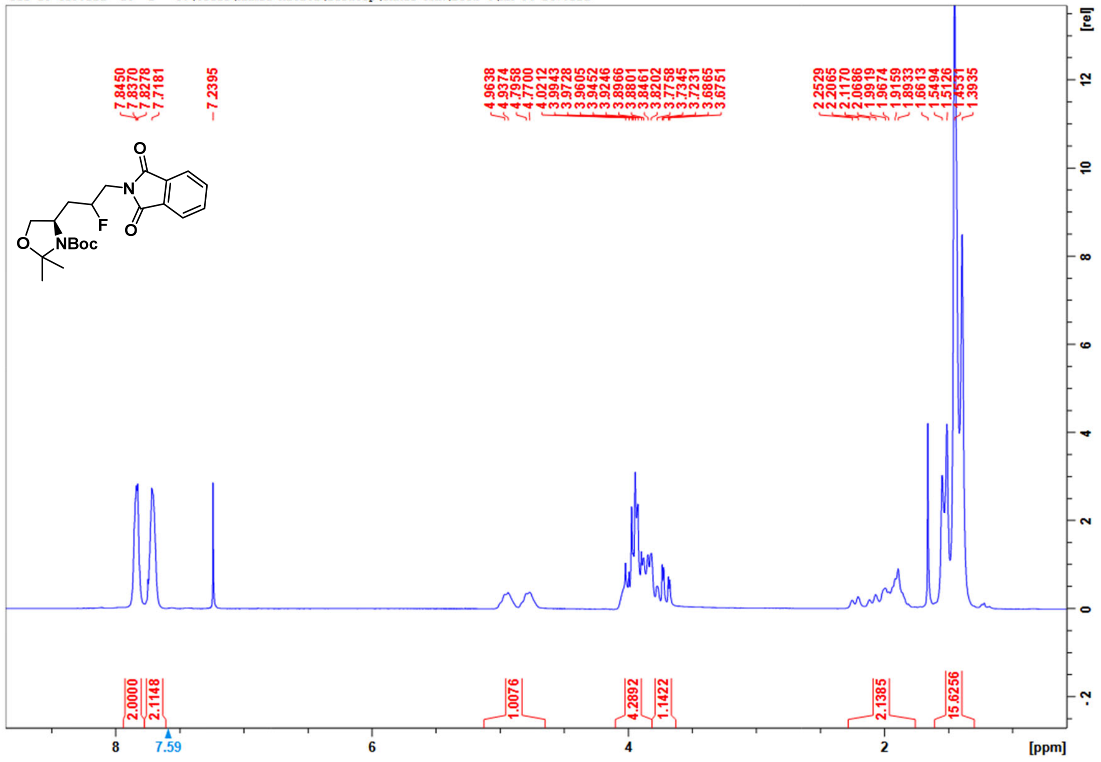

Yzz-15-62CP.fid 10 1 "C:\Users\Ahmad Altiti\Desktop\TAXIS-NMR\Book-4\AA-04-167.fid"

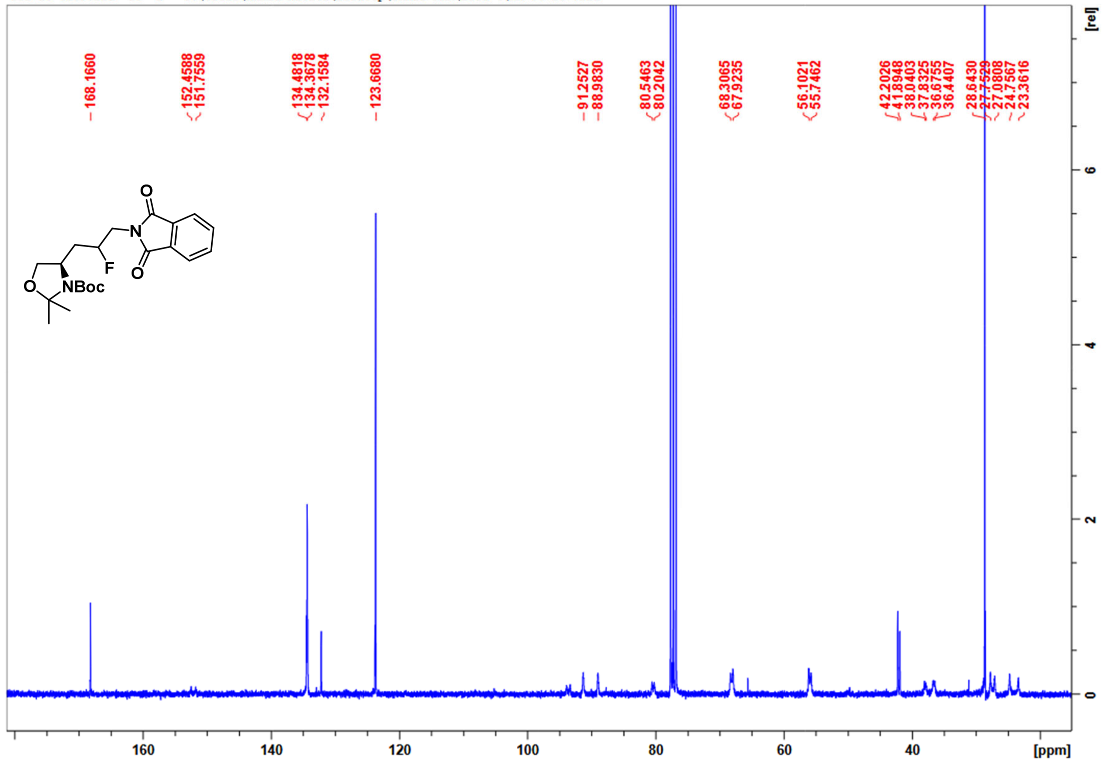

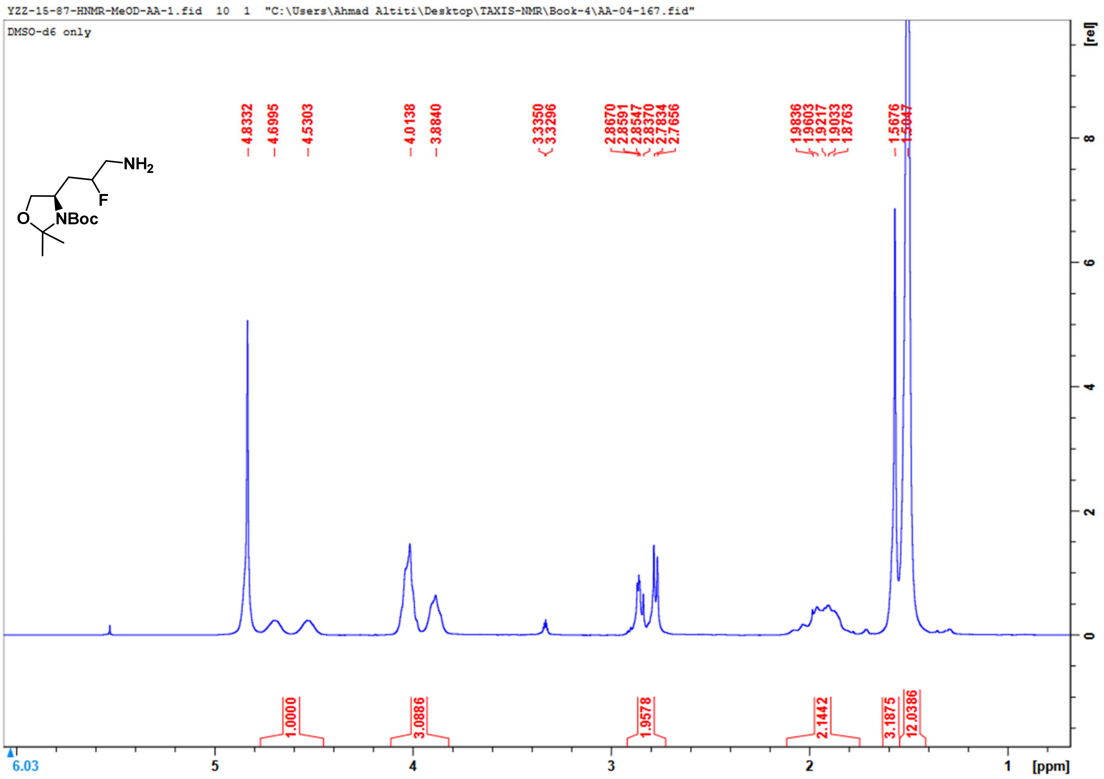
YZZ-15-87-HNMR-MeOD-AA-1.fid 10 1 "C:\Users\Ahmad Altiti\Desktop\TAXIS-NMR\Book-4\AA-04-167.fid"
DMSO-d6 only
NH2
F
O
NBoc
4.8332
4.6995
4.5303
4.0138
3.8840
3.3350
3.3296
2.8670
2.8591
2.8547
2.8370
2.7834
2.7656
1.9836
1.9603
1.9217
1.9033
1.8763
1.5676
1.0000
3.0886
1.9578
2.1442
3.1875
12.0386
6.03
[ppm]
[rel]

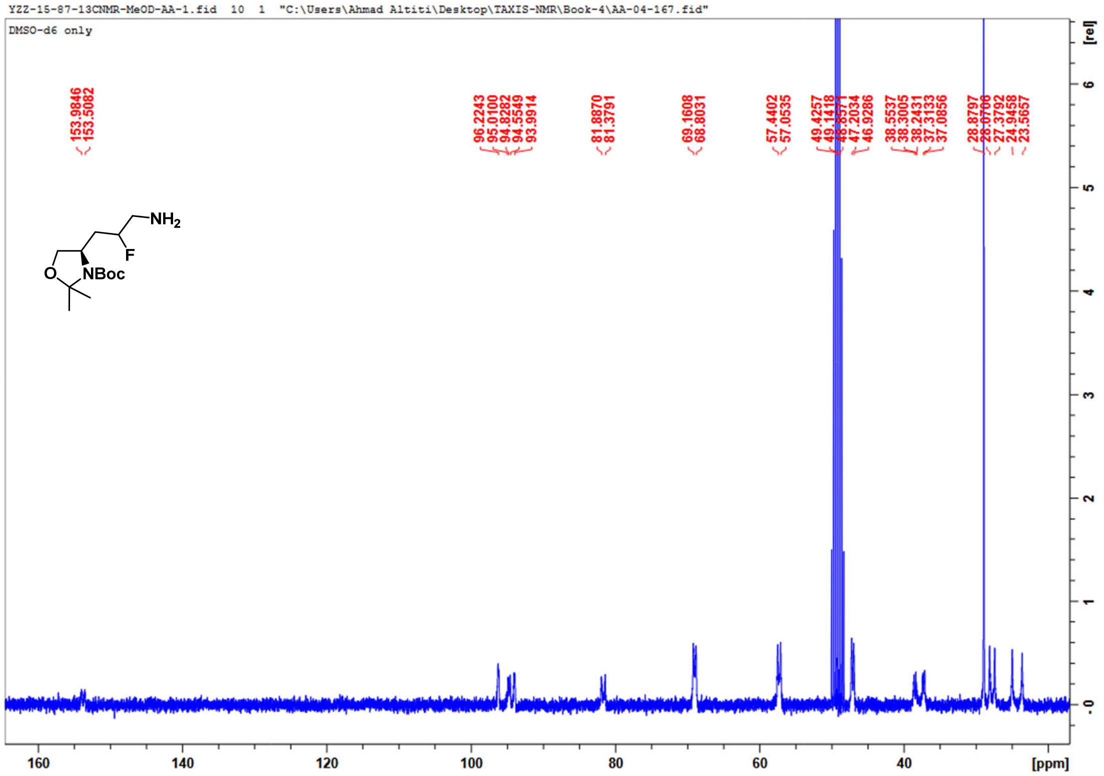
YZZ-15-87-13CNMR-MeOD-AA-1.fid 10 1 "C:\Users\Ahmad Altiti\Desktop\TAXIS-NMR\Book-4\AA-04-167.fid"
DMSO-d6 only
153.9846
153.5082
96.2243
95.0100
94.8262
94.5549
93.9914
81.8870
81.3791
69.1608
68.8031
57.4402
57.0535
49.4257
47.2034
46.9286
38.5537
38.3005
38.2431
37.3133
37.0856
28.8797
27.3792
24.9458
23.5657
NH2
F
O
NBoc
[ppm]
[rel]

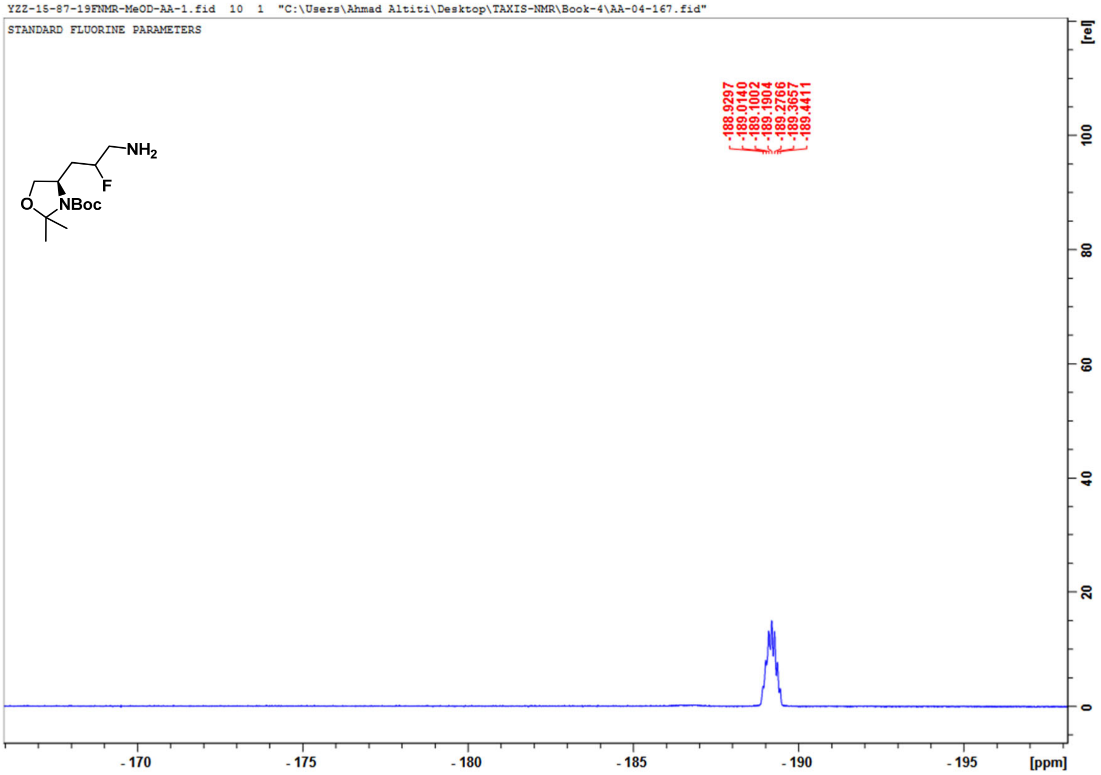
YZZ-15-87-19FNMR-MeOD-AA-1.fid 10 1 "C:\Users\Ahmad Altiti\Desktop\TAXIS-NMR\Book-4\AA-04-167.fid"
STANDARD FLUORINE PARAMETERS
-188.9297
-189.0140
-189.1002
-189.1904
-189.2766
-189.3657
-189.4411
NH2
F
O
NBoc
[rel]
[ppm]

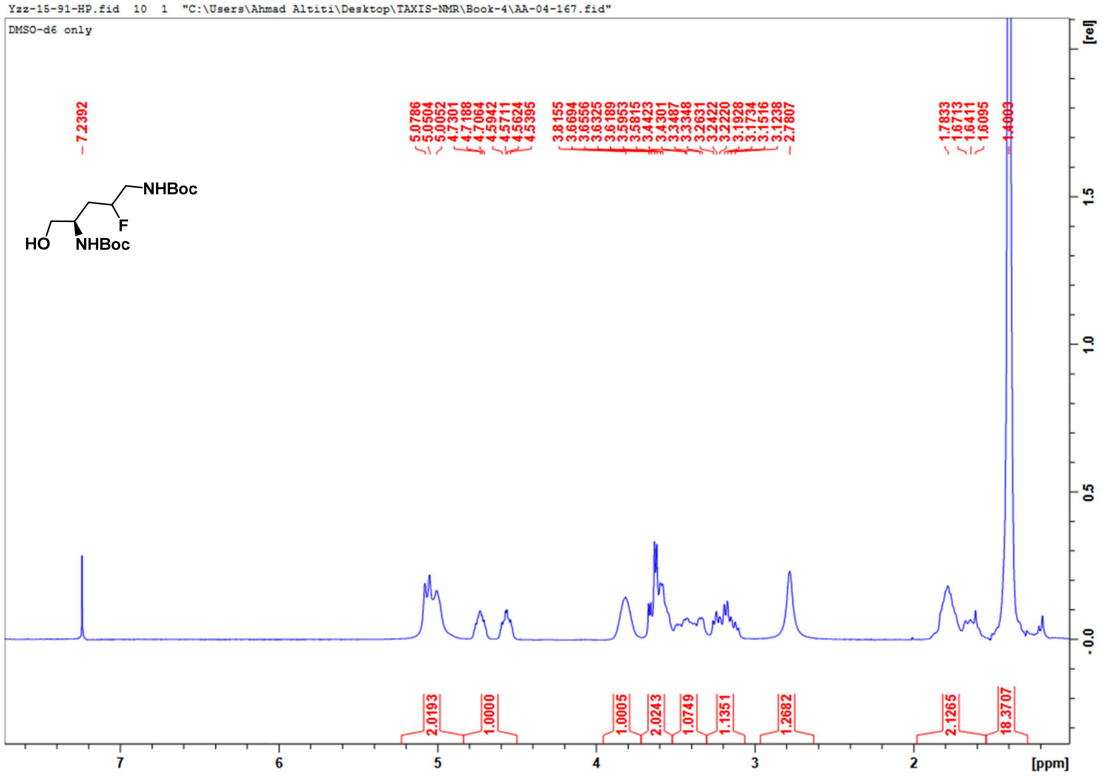
Yzz-15-91-HP.fid 10 1 "C:\Users\Ahmad Altiti\Desktop\TAXIS-NMR\Book-4\AA-04-167.fid"
DMSO-d6 only
7.2392
5.0786
5.0504
5.0052
4.7301
4.7188
4.7064
4.5942
4.5711
4.5624
4.5395
3.8155
3.6694
3.6556
3.6325
3.6189
3.5953
3.5815
3.4423
3.4301
3.3487
3.3348
3.2631
3.2422
3.2220
3.1928
3.1734
3.1516
3.1238
2.7807
1.7833
1.6713
1.6411
1.6095
1.4003
NHBoc
F
HO
NHBoc
2.0193
1.0000
1.0005
2.0243
1.0749
1.1351
1.2682
2.1265
18.3707
[rel]
[ppm]

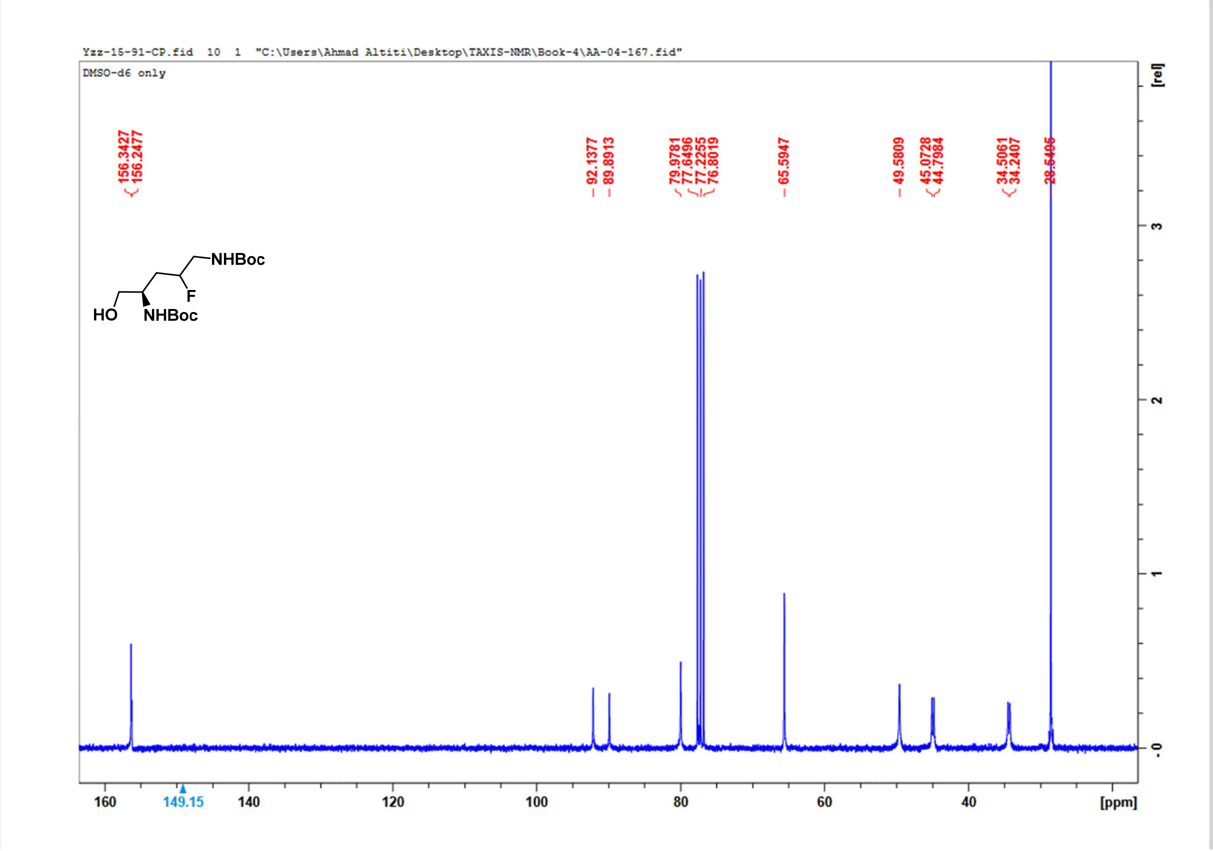
Yzz-15-91-CP.fid 10 1 "C:\Users\Ahmad Altiti\Desktop\TAXIS-NMR\Book-4\AA-04-167.fid"
DMSO-d6 only
156.3427
156.2477
92.1377
89.8913
79.9781
77.6496
77.2255
76.8019
65.5947
49.5809
45.0728
44.7984
34.5061
34.2407
28.5496
NHBoc
F
HO
NHBoc
160
149.15
140
120
100
80
60
40
[ppm]
[rel]

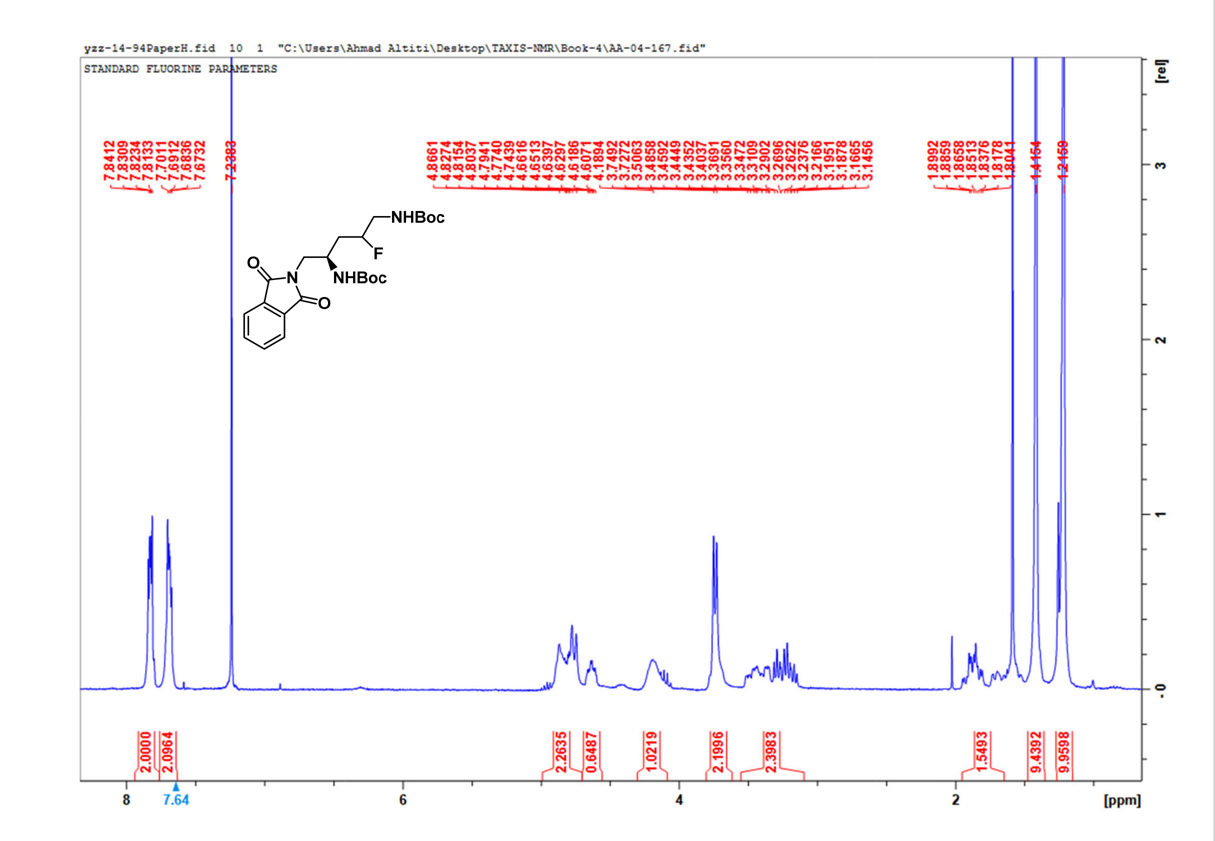
yzz-14-94PaperH.fid 10 1 "C:\Users\Ahmad Altiti\Desktop\TAXIS-NMR\Book-4\AA-04-167.fid"
STANDARD FLUORINE PARAMETERS
7.8412
7.8309
7.8234
7.8133
7.7011
7.6912
7.6836
7.6732
7.2383
NHBoc
F
O
N
NHBoc
O
2.0000
2.0964
2.2635
0.6487
1.0219
2.1996
2.3983
1.5493
9.4392
9.9598
8
7.64
6
4
2
[ppm]
[rel]

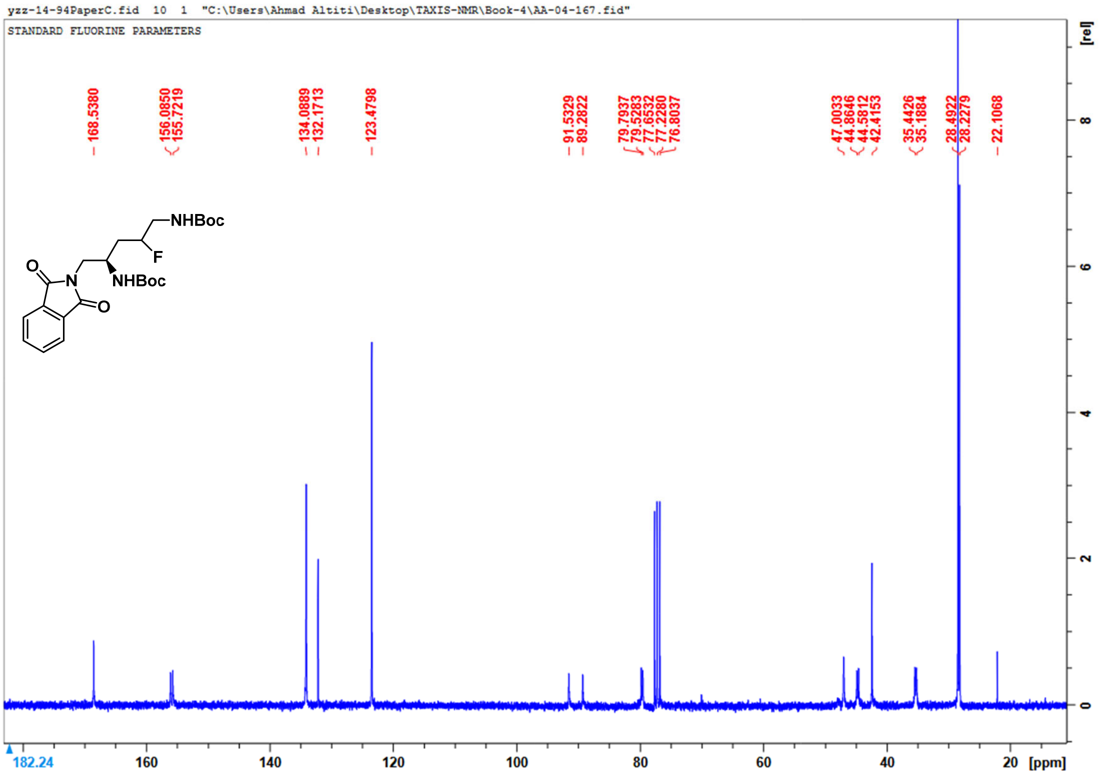
yzz-14-94PaperC.fid 10 1 "C:\Users\Ahmad Altiti\Desktop\TAXIS-NMR\Book-4\AA-04-167.fid"
STANDARD FLUORINE PARAMETERS
NHBoc
F
NHBoc
N
O
O

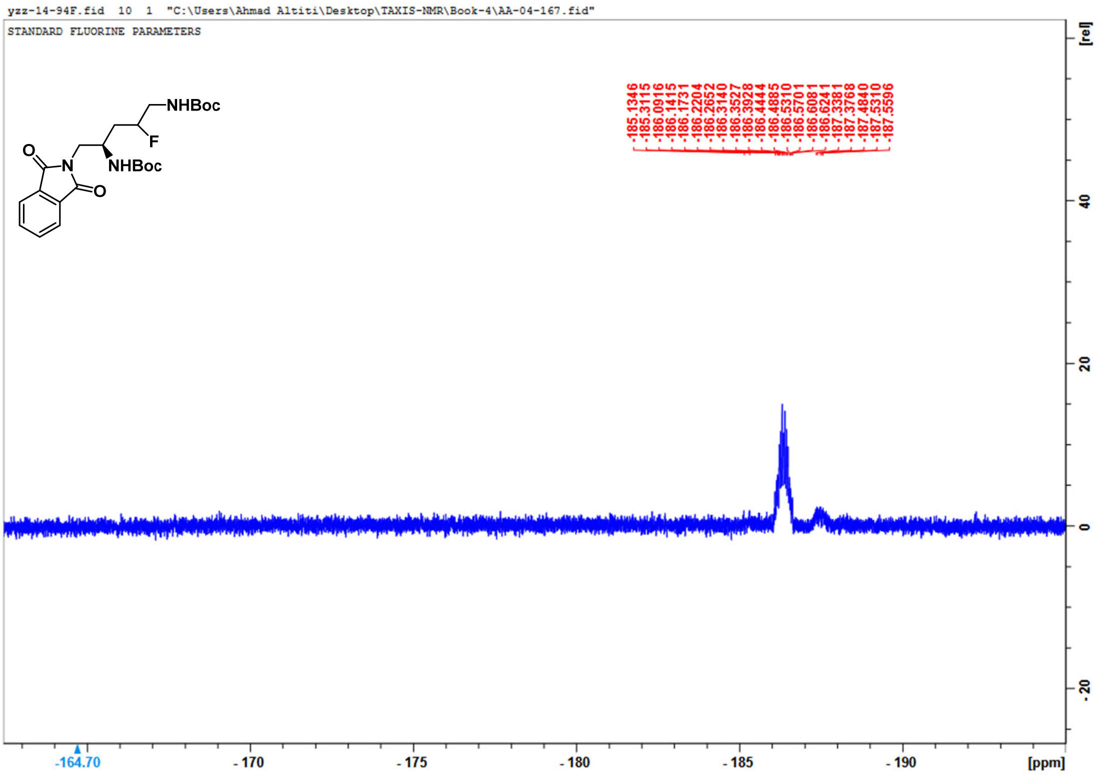
yzz-14-94F.fid 10 1 "C:\Users\Ahmad Altiti\Desktop\TAXIS-NMR\Book-4\AA-04-167.fid"
STANDARD FLUORINE PARAMETERS
NHBoc
F
NHBoc
N
O
O
